# Hit-Identification to Novel Antileishmanial Agents from a β‑Pinene Scaffold: from Synthesis to *In Vitro* Evaluation and *In Silico* SAR/ADMET Profiling

**DOI:** 10.1021/acsomega.5c08971

**Published:** 2026-01-20

**Authors:** Gustavo dos S. Martins, Bruno M da S Santos, Yago S. S. Emiliano, João Pedro A. Santos, Gérzia M. Machado, Mariana S. de Carvalho, Kamila Marques Sette, Igor de A. Rodrigues, Alessandra M. T de Souza, Eduardo Caio Torres-Santos, Fernanda G Finelli, Ivana Correa Ramos Leal

**Affiliations:** † Laboratory of Natural Products and Biological Assays (LaProNEB), Department of Natural Products and Food, Faculty of Pharmacy, 28125 Federal University of Rio de Janeiro, UFRJ, Rio de Janeiro 21941-902, Brazil; ‡ Laboratory of Organic Synthesis, Instituto de Pesquisa de Produtos Naturais, Federal University of Rio de Janeiro, UFRJ, Rio de Janeiro 21941-902, Brazil; § Laboratory of Trypanosomatid Biochemistry, 37903 Instituto Oswaldo Cruz, FIOCRUZ, Rio de Janeiro 21040-360, Brazil; ∥ Laboratory of Molecular Modelling QSAR, Faculty of Pharmacy, Federal University of Rio de Janeiro, UFRJ, Rio de Janeiro 21941-170, Brazil; ⊥ Laboratory of Investigation of Bioactive Substances, Department of Natural Products and Food, Faculty of Pharmacy, Federal University of Rio de Janeiro, UFRJ, Rio de Janeiro 21941-170, Brazil

## Abstract

β-Pinene, a low-cost natural product derived from agricultural waste, has shown *in vitro* activity against *Leishmania amazonensis*, but its use is hindered by unfavorable pharmacokinetic properties. Herein, we report a straightforward two-step synthesis of β-pinene-derived hydroxysulfides followed by an *in vitro* evaluation of their antileishmanial activity, cytotoxicity profile in mammalian cells, and *in silico* studies of structure–activity relationship (SAR) and ADMET properties. Initially, β-pinene was converted into its epoxide, the key intermediate of the series, through both chemoenzymatic and nonchemoenzymatic approaches. Then, we studied the thiolysis reaction by screening a series of bases and solvents. The use of NaOMe in methanol afforded the β-hydroxysulfide in 81% yield. This strategy afforded 16 novel derivatives bearing alkyl and (hetero)­aryl substituents, with isolated yields ranging from 19 to 91%. The antileishmanial activity with promastigote cells showed that 11 compounds reduced parasite viability to <10% in a fixed-concentration assay (100 μM), and six displayed IC_50_ values below 30 μM. Four derivatives were further evaluated against intracellular amastigote cells, with the *para*-fluoroaryl analogue emerging as a hit compound (IC_50_ = 6.3 μM; SI > 15.9). SAR analysis revealed key physicochemical features associated with activity, highlighting the importance of lipophilicity, polar surface area, and cLogP in promoting parasite membrane penetration. Meanwhile, *in silico* ADMET supported their drug-likeness since no mutagenic, cardiotoxic, or hepatotoxic potential was predicted, encouraging further *in vivo* and mechanistic studies.

## Graphical abstract

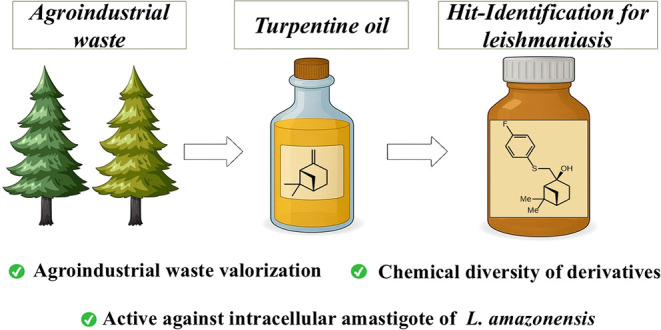

## Introduction

Cutaneous leishmaniasis is a neglected tropical disease caused by parasites of the genus *Leishmania*, such as *Leishmania amazonensis* and *Leishmania infantum*. It is transmitted to humans and other mammals by the bite of infected phlebotomine sandflies.^1,2^ According to the World Health Organization (WHO), approximately one million new cases and 30,000 deaths occur annually, with the highest burden in countries such as Brazil, Afghanistan, Peru, and Colombia. The incidence has been rising, driven by coinfections (e.g., HIV/AIDS) and environmental changes, such as deforestation and urbanization.^2,3^

Current chemotherapies for the treatment of leishmaniasis remain far from ideal due to the high toxicity, adverse side effects, drug resistance, elevated costs, and poor accessibility in resource-limited regions. These challenges highlight the urgent need for safer, more effective, and widely available antileishmanial therapeutic alternatives.^4,5^

The development of new antileishmanial agents requires rational target selection and mechanistic understanding. The trypanothione/trypanothione reductase (TR) system represents a validated parasite-specific drug target. Unlike mammalian cells that rely on glutathione and glutathione reductase for redox homeostasis, Leishmania parasites depend exclusively on trypanothione (a bis-glutathionyl-spermidine conjugate) and TR for maintaining intracellular redox balance and detoxifying reactive oxygen species.^6,7^ This unique metabolic pathway is essential for parasite survival and has no direct mammalian homologue, making it an attractive target for selective chemotherapy.^8^ Notably, sulfur-containing compounds, particularly diaryl sulfides and related scaffolds, have emerged as promising TR inhibitors with demonstrated antileishmanial activity both *in vitro* and *in vivo*.^9,10^ These compounds typically act by binding to the TR active site, disrupting trypanothione reduction and leading to oxidative stress-mediated parasite death.^11^

Natural products constitute a valuable source of bioactive molecules. Terpenes represent the most diverse class of secondary metabolites, with more than 40,000 compounds identified to date, and display remarkable biological potential against various parasites,^12–14^ particularly *Leishmania* species.^15,16^ Pentacyclic type-triterpenes such as lupeol and morolic acid have shown promising antileishmanial effects, while enantiomers of the monoterpene β-pinene exhibit distinct activities against *L. amazonensis* ^16^ (Scheme 1). In particular, (+)-β-pinene displays higher potency than (−)-β-pinene;^17^ nonetheless, both therapeutic potentials are hindered by poor aqueous solubility and rapid hepatic metabolism by CYPs.^18^ Conversely, (−)-β-pinene is the major component of turpentine oil (*Pinus spp*.), an agro-industrial byproduct of the paper and citrus juice industries, produced on a scale of approximately 330,000 tons per year and available at low cost (∼USD 200 per ton).^19,20^ It has been widely used as a versatile building block in synthetic chemistry,^19^ capable of yielding derivatives with diverse and potential pharmacological activities.^21–24^

**Scheme 1:**
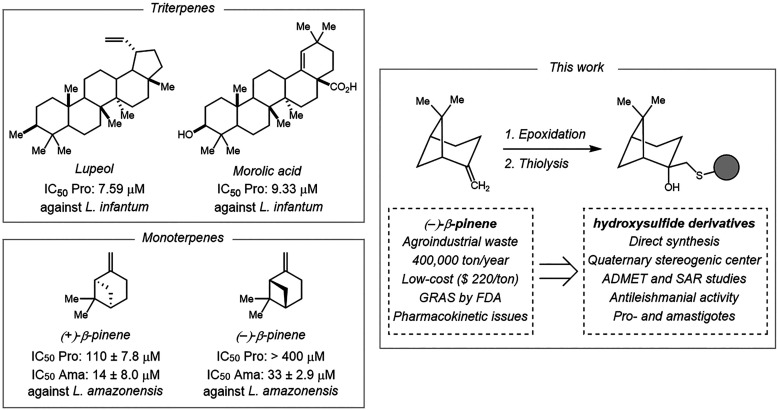
Pentacyclic Type-Triterpenes Skeletons and β-Pinene Monoterpene Scaffold Proposed for This Work as Potent Antileishmanials

Several sulfur-containing derivatives have already shown activity against different *Leishmania* species. For example, 2-aminothiophene,^25^ 2-mercaptobenzimidazole, and sulfonamide derivatives were active against *L. braziliensis*, *L. major*, *L. infantum*, and *L. mexicana*, and their activity was associated with membrane disruption and inhibition of key parasite enzymes.^26,27^ Thus, we hypothesized that hydroxysulfide derivatives of (−)-β-pinene could represent a promising new scaffold for more attractive antileishmanial molecules. Herein, we report the synthesis of a series of novel β-hydroxysulfides from the natural β-pinene through an efficient process involving epoxidation, by chemoenzymatic and nonchemoenzymatic approaches, followed by thiolysis reactions (Scheme 1). The derivatives were evaluated against the promastigote and amastigote forms of *L. amazonensis* *in vitro* as well as for their cytotoxicity toward mammalian cells (RAW 264.7 and VERO cell lines) to determine the selectivity index. Furthermore, their ADMET profiles and structure–activity relationships (SAR) were investigated *in silico*.

## Results and Discussion

### Synthesis

The synthetic route to the series of β-hydroxysulfide derivatives was based on the epoxidation of (−)-β-pinene (**1**), followed by thiolysis. Our investigation began with the optimization of the epoxidation step using both chemoenzymatic (Lipase Novozyme 435; entries 4–8, Supporting Information, Section 2, Table S1) and nonchemoenzymatic approaches (entries 1–3, Supporting Information, Section 2, Table S1).^28–30^ The reactions afforded the desired epoxide **2** in 39–70% yield, with the chemoenzymatic route furnishing the highest one and diastereoselectivities (dr) ranging from 3:1 to >95:5 (see Supporting Information, Section 2, Table S1). After optimization, epoxide **2** was prepared on a gram scale by reacting β-pinene (1 equiv) with Oxone (1 equiv) and NaHCO_3_ (1 equiv) in a 3:2 H_2_O/acetone mixture at room temperature for 30 min, yielding 44% (−)-β-pinene with a 12:1 dr.

Thiolysis was then evaluated under both basic and acid conditions. Under acid conditions, the generation of a carbocationic character within the β-pinene ring led to rapid decomposition, yielding isomerization products such as fenchane, bornane, and perillyl alcohol derivatives.^31^ This outcome was observed even when employing more compatible Lewis acids (e.g., Si, Fe, and Al salts) or Brønsted acids such as ammonium chloride (data not shown). In contrast, the desired transformation was achieved under milder basic reaction conditions, with the best result (81% isolated yield) obtained using NaOMe in methanol at 65 °C for 6 h (see Supporting Information, Section 3, Table S2, entry 11).

This approach afforded a library of β-hydroxysulfides **3** derivatives using commercially available thiols as nucleophiles bearing diverse alkyl and (hetero)­aryl substituents. In total, 16 compounds (**3a**–**3p**) were synthesized and isolated in yields ranging from 19 to 91% (Scheme 2). The methodology enabled the synthesis of β-hydroxysulfides bearing a wide range of substituents, including alkyl chains (**3b** and **3j**), benzylic groups (**3d**), electron-donating groups (**3c**, **3f**, **3g**, **3i**, **3k**, **3m**), and heteroaryl moieties (**3h**, **3l**, **3o**, and **3p**) (See Supporting Information, Section 4). The presence of functional handles such as Cl and NH_2_ offers opportunities for further functionalization via cross-coupling reactions, enabling the preparation of novel and several derivatives. Additionally, various heteroaromatic rings (e.g., pyridine, pyrimidine, and benzimidazole) were incorporated, which are considered privileged scaffolds in medicinal chemistry due to their broad spectrum of pharmacological activities.^27^

**Scheme 2:**
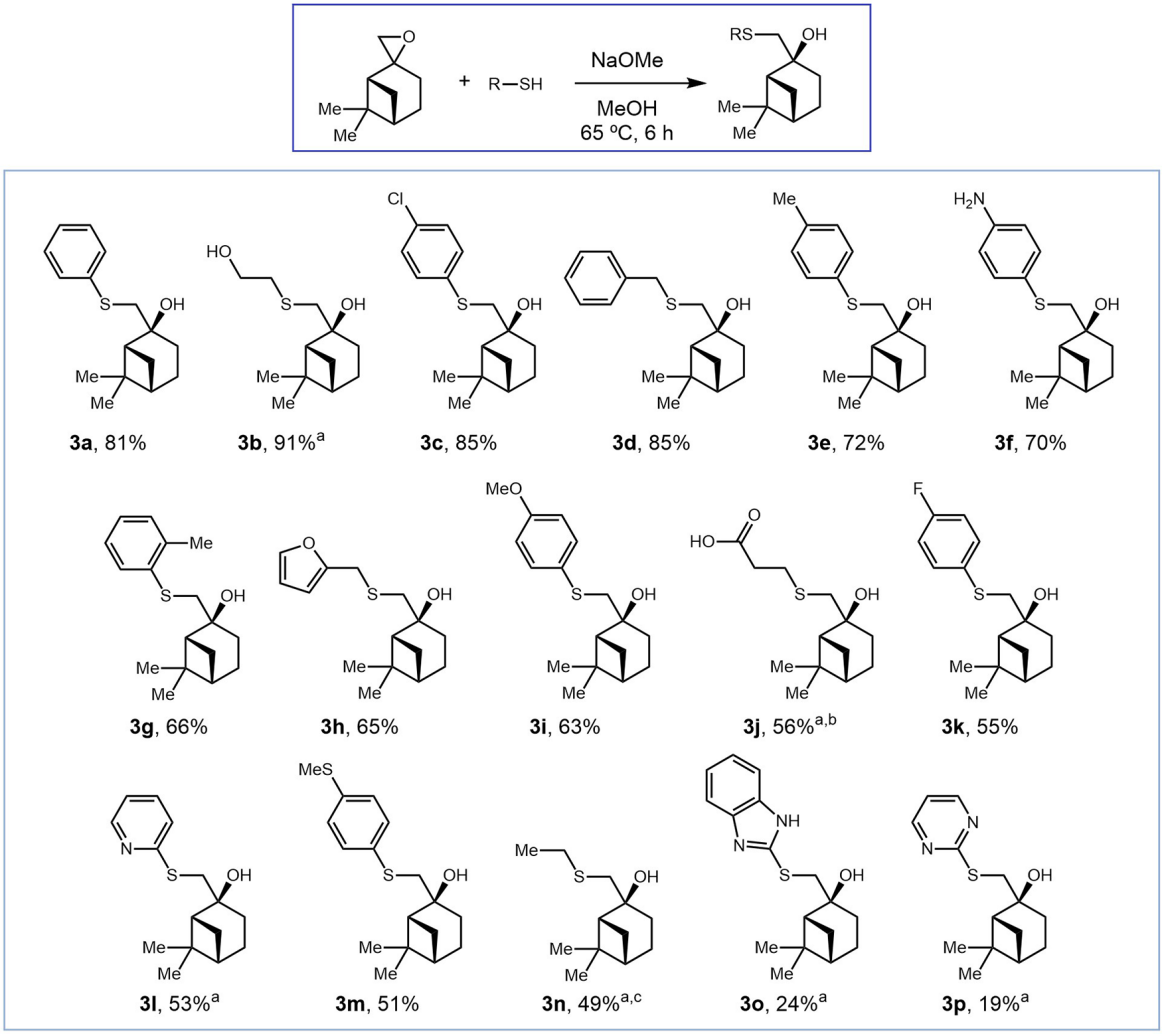
Synthesis of β-Hydroxysulfide Derivatives **3** ^a^

a Epoxidation reaction performed with (−)-β-pinene (**1**) (1 equiv), oxone (1 equiv), NaHCO_3_ (1 equiv) in a 3:2 H_2_O/acetone mixture for 30 min at room temperature. Thiolysis reaction performed with (−)-β-pinene epoxide **2** (1 equiv), NaOMe (1.6 equiv), thiol (1.6 equiv) in methanol for 6 h at 65 °C. ^[a]^Thiolysis reactions performed for 48 h. ^[b]^Thiolysis reactions performed with 2.6 equiv of NaOMe. ^[c]^Thiolysis reactions performed at room temperature.

The reactions proceeded with high chemoselectivity, even in the presence of competing nucleophilic groups (e.g., NH_2_, OH), without the formation of side products. The highest isolated yield (91%) was obtained with mercaptoethanol (Scheme 2; **3b**), followed by thiophenols bearing electron-donating groups (Scheme 2; **3c**) and benzylic thiols (Scheme 2; **3d**). In contrast, aliphatic and heteroaryl thiols led to a more time-consuming reaction due to their lower acidity, which limits thiolate formation and possible thiol-thione tautomerization, reducing nucleophile availability. In some cases, additional purification steps (e.g., **3o**, **3p**) unfortunately contributed to reduced isolated yields.

### Biological Evaluation

The β-hydroxysulfide derivatives were subsequently evaluated for their *in vitro* activity against *L. amazonensis* (MHOM/BR/77/LTB0016) promastigotes. At 100 μM, 11 compounds reduced parasite viability to <10% after 72 h (see Supporting Information, Section 6, Item 6.1, Figure S2) and were selected for further IC_50_ determination. IC_50_ values were determined by resazurin colorimetric assay after 72 h of incubation with derivatives **(except 3b, 3j, 3n)** (three independent experiments, in triplicate), and the same compounds were assessed for cytotoxicity in RAW 264.7 and VERO cells to calculate selectivity indexes (SI). As summarized in Table 1, compounds **3d**, **3e**, **3f**, **3g**, **3i**, **3k**, and **3o** exhibited IC_50_ values below 30 μM. Among them, **3f**, **3i**, and **3k** combined potency (low IC_50_) with a favorable selectivity index toward RAW cells (SI > 3.8), whereas **3d**, **3e**, and **3m** displayed marked cytotoxicity against RAW 264.7. The high SI values of compounds **3f**, **3i**, **3k**, and **3o** toward murine macrophages (RAW) are particularly relevant, since *Leishmania* replicates intracellularly within these host cells.

**Table 1:** Determination of Mean Inhibitory Concentration (IC_50_) against Promastigotes of *L. amazonensis* and Determination of Mean Cytotoxic Concentration (CC_50_) against Macrophages RAW 264.7 and Vero Cell Lines; Selectivity indexes (SI) Were Also Calculated^d^

compound	IC_50_ promastigote(μM)^a^	CC_50_ RAW 264.7^a^ (μM)^a^	CC_50_ VERO (μM)^a^	SI (CC_50_ RAW 264.7/IC_50_)^b^	SI (CC_50_ VERO/IC_50_)^b^
**3d**	22.9 ± 1.9	48.59 ± 8.27	>100	2.1	>4.4
**3e**	20.9 ± 1.2	28.51 ± 2.09	>100	1.4	>4.8
**3f**	26.3 ± 3	>100	>100	>3.8	>3.8
**3g**	21.5 ± 3.6	80.87 ± 1.6	99.1 ± 0.4	3.8	4.6
**3h**	39.0 ± 1.5	>100	>100	>3.5	>3.5
**3i**	19.7 ± 0.9	97.01 ± 2.27	>100	4.9	>5.1
**3k**	20.0 ± 1.8	>100	>100	>5	>5
**3l**	38.7 ± 3.6	>100	>100	>2.6	>2.6
**3m**	31.5 ± 3.3	28.85 ± 1.58	>100	0.9	>3.2
**3o**	29.7 ± 0.2	>100	39.2 ± 4	>3.3	1.3
**3p**	69.7 ± 4	>100	>100	>1.4	>1.4
Amphotericin B^c^	0.74 ± 0.1	8.1 ± 0.3	8.1	-	-
Miltefosine	5.4 ± 0.8	-	-	-	-

a The half-maximal antileishmanial (IC_50_), half-maximal cytotoxic against RAW 264.7, and VERO (CC_50_) are reported as mean values (μM ± standard error) of three independent experiments.

b The selectivity index (SI) was calculated as the ratio of CC_50_ to IC_50_ (promastigotes).

c Expressed in μg.mL^–1^, according to Adão and co-workers.^17^

d Selectivity indexes (SI) were also calculated.

### *In Silico* Studies

A structure–activity relationship (SAR) analysis was performed to correlate the chemical features of the derivatives with their activity against *L. amazonensis* promastigotes, considering IC_50_, molecular volume (MV, **Å** ^**3**^), and polar surface area (PSA, **Å** ^**2**^) (Table 2). Overall, heterocyclic substituents (**3h**, **3l**, **3p**) reduced activity compared to phenyl/benzyl groups, with **3o** as an exception due to its benzimidazole scaffold, previously reported as active against *Leishmania* spp.^27^

**Table 2:**
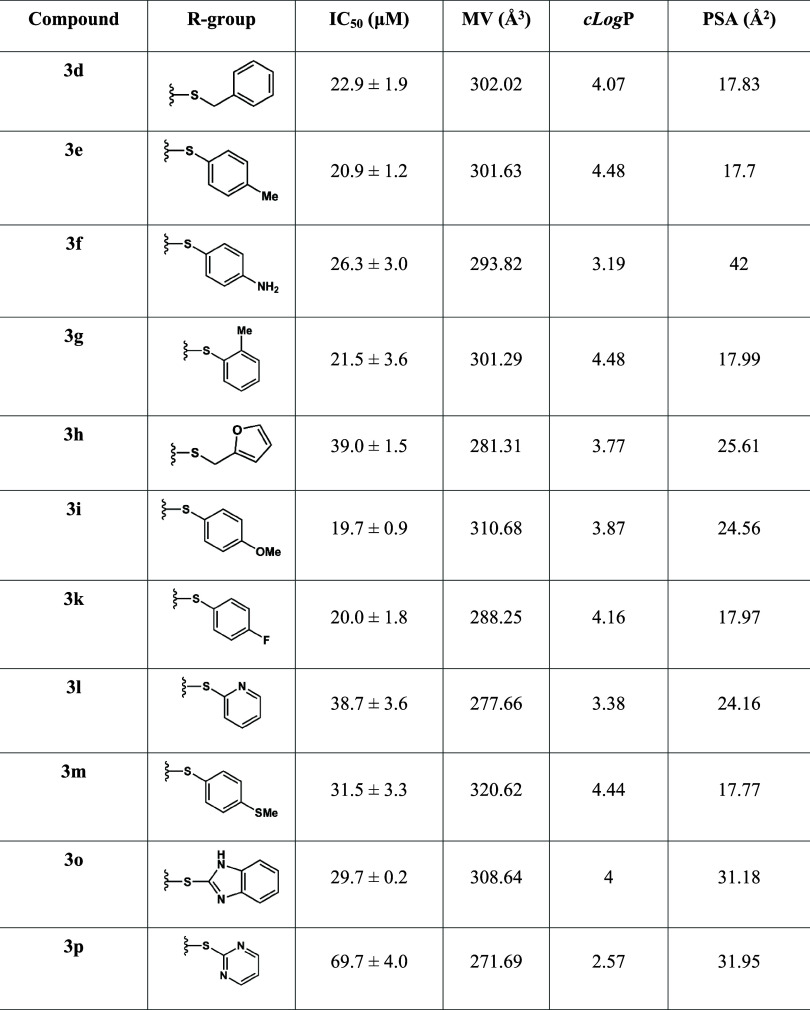
Comparison of *In Vitro* Leishmanicidal Activity against Promastigotes (IC_50_, μM) and the Molecular Descriptors Molecular Volume (MV, Å^3^) and Polar Surface Area (PSA, Å^2^) of β-Pinene Derivatives

Lipophilicity emerged as a key physicochemical property for enhancing membrane permeability and hence the antileishmanial activity. So, as the presence of heteroatoms decreases lipophilicity, leishmanial activity was reduced. With the introduction of methylene spacer or methyl groups (**3d**, **3e**, **3f**), the lipophilicity is maintained (ranging between 4.07 and 4.48), and the orientation of the substitution does not affect the leishmanicidal activity significantly (Figure 1B). Substitution with an electronegative group that activates the ring through resonance or inductive effect appeared to enhance the activity of compounds **3d, 3f, 3i**, and **3f** when compared to other molecules in the series (Figure 1A). In these instances, we can reinforce that MV, ranging from 285 to 310 A^3^, is a common factor among molecules with good biological activity.

**Figure 1:**
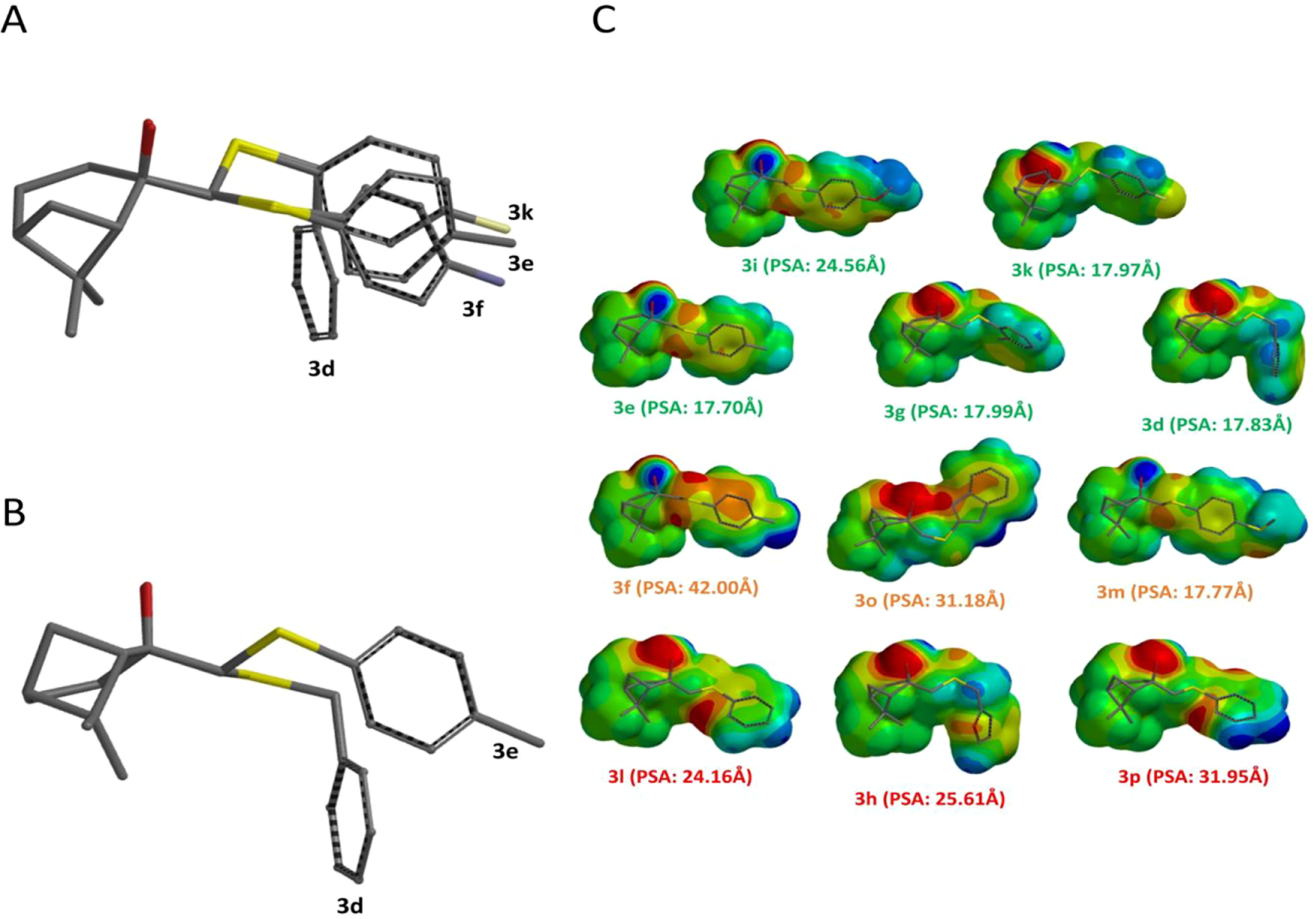
Structural analysis and electrostatic properties of β-pinene derivatives. (A) 3D structures of compounds **3d**, **3e**, **3f**, and **3k**, highlighting that certain substituents activate the ring, suggesting a relationship with the biological activity efficacy. (B) Structures of compounds **3e** and **3d**, demonstrating that the position of the substituents on the ring does not significantly influence biological activity, indicating that the presence of activating groups is more important than their location. (C) Electrostatic potential maps of the compounds, with the respective PSA values (polar surface area) in Å^2^. The analysis suggests that compounds with higher PSA values show lower biological activity, indicating that PSA is a crucial factor in modulating the pharmacological efficacy of the compounds.

As well as lipophilicity and MV, PSA also seems to be related to leishmanicidal activity (Table 2). PSA significantly influences the overall polarity of a molecule, thereby affecting its capacity to permeate biological membranes (Figure 1C). As can be seen in Table 2, PSA also correlated with potency, as all active compounds showed PSA < 60 Å^2^, consistent with literature indicating that values below this threshold favor passive membrane diffusion.^32,33^

Finally, given the three-dimensional structure of the derivatives, it could be observed that the hydroxyl groups of the β-pinene ring were consistently directed toward the sulfur lone pairs in all compounds. The only exception is **3o**, where bonding occurred with a nitrogen atom from the benzimidazole ring. This conformation facilitates intramolecular hydrogen bonds, which generally increase lipophilicity by reducing the molecule’s polar character. This feature reinforces the importance of lipophilicity in this parasitic activity (Figure 1C)

In summary, lipophilicity (cLogP) emerged as a key factor for permeability and function, with an optimal value exceeding 3.8, while the PSA should remain below 25 Å^2^ in agreement with the trend observed within the most active compound and the general threshold reported in the literature (PSA < 60 Å^2^), and molecular volume should be at about 300 ± 11Å^3^.

Compounds **3f**, **3i**, **3k**, and **3o**, which displayed the most promising promastigote activity (IC_50_ ≈ 20–30 μM; CC_50_ ≥ 90 μM for RAW 264.7 cell line) were selected for *in vitro* evaluation against intracellular amastigotes. Notably, these compounds showed low cytotoxicity toward VERO cells, a kidney-derived epithelial cell line, which is a relevant advantage considering that most clinically antileishmanial agents are associated with nephrotoxicity.^34^

As can be seen in Table 3, compounds **3k** and **3o** showed to be active against intracellular amastigotes, with low IC_50_ values of 6.3 and 21.2 μM, respectively (see Supporting Information Section 6, Item 6.3, Figures S3 and S4). Among them, **3k** emerged as the most promising compound, showing IC_50_ < 10 μM and SI > 15.9, outperforming its activity against intracellular amastigotes and meeting international criteria for antileishmanial drug discovery (IC_50_ < 10 μM; SI > 10.3), and was obtained in a synthetic route with only two steps.^3,35^

**Table 3:** Determination of IC_50_ and Selectivity Index (SI) of Selected Compounds against the Intracellular Amastigote of *L. amazonensis* ^a^

compound	IC_50_ intracellular amastigotes *L. amazonensis* (μM)	CC_50_ RAW 264.7 cell lines (μM)	selectivity index (SI)
**3f**	>50	>100	n.d
**3i**	>50	97.01 ± 2.27 b	n.d
**3k**	6.3 ± 0.8^b^	>100	>15.9
**3o**	21.2 ± 0.6	>100	>4.7

a Macrophages were infected with *L. amazonensis* promastigotes for 5 h at 37 °C and subsequently incubated in the absence or presence of varying concentrations (1.5–50 μM) of the derivatives for 72 h. A total of 200 macrophages were counted on each coverslip in duplicate, and the infection index was determined. The values represent the mean ± standard error of three independent experiments (*n* = 3). The IC_50_ was calculated by nonlinear regression using GraphPad Prism version 10.0.

b A statistically significant difference compared to the **3k** with the control was observed (****p < 0.0001). n.d. = *not determined*

Based on the structures of **3k** and **3o**, different mechanisms of action can be proposed. For instance, alkylation of 2-mercaptobenzimidazole derivatives has been reported to enhance their affinity for *L. mexicana* arginase, a key enzyme in polyamine biosynthesis. Molecular docking simulations by Betancourt-Courte and co-workers^27^ demonstrated significant π–π interactions involving the benzimidazole ring, as well as hydrogen bonding and van der Waals interactions within the enzyme’s active site. Therefore, we hypothesize that the elongation of the alkyl chain in **3o**, provided by the β-pinene scaffold, may strengthen these interactions and improve the inhibition of this target, while the insertion of a hydroxyl group could offer an additional site for hydrogen bonding. Meanwhile, the incorporation of fluorine in derivative **3k** increases lipophilicity (cLogP 4.16), which may favor membrane permeation—a behavior previously reported for β-pinene^17,36^ and sulfur-containing derivatives—^37^ and also could favor interactions with parasite targets.^38^ To confirm these hypotheses, *in silico* and *in vitro* studies are currently underway.

To further assess its potential as a hit compound, **3k** was analyzed for *in silico* pharmacokinetics and toxicological profile. It violated only one of Lipinski’s rules, suggesting good oral bioavailability (Figure 2).^39^ No mutagenic, cardiotoxic, or hepatotoxic potential was predicted, and carcinogenicity was classified as low (TD_50_ > 5 mg/kg/day), in contrast with miltefosine, which has already been demonstrated to be carcinogenic and to present several other side effects that limit its clinical use.^40^ Finally, skin sensitization assays suggested that topical formulations may require excipients to mitigate this effect.

**Figure 2:**
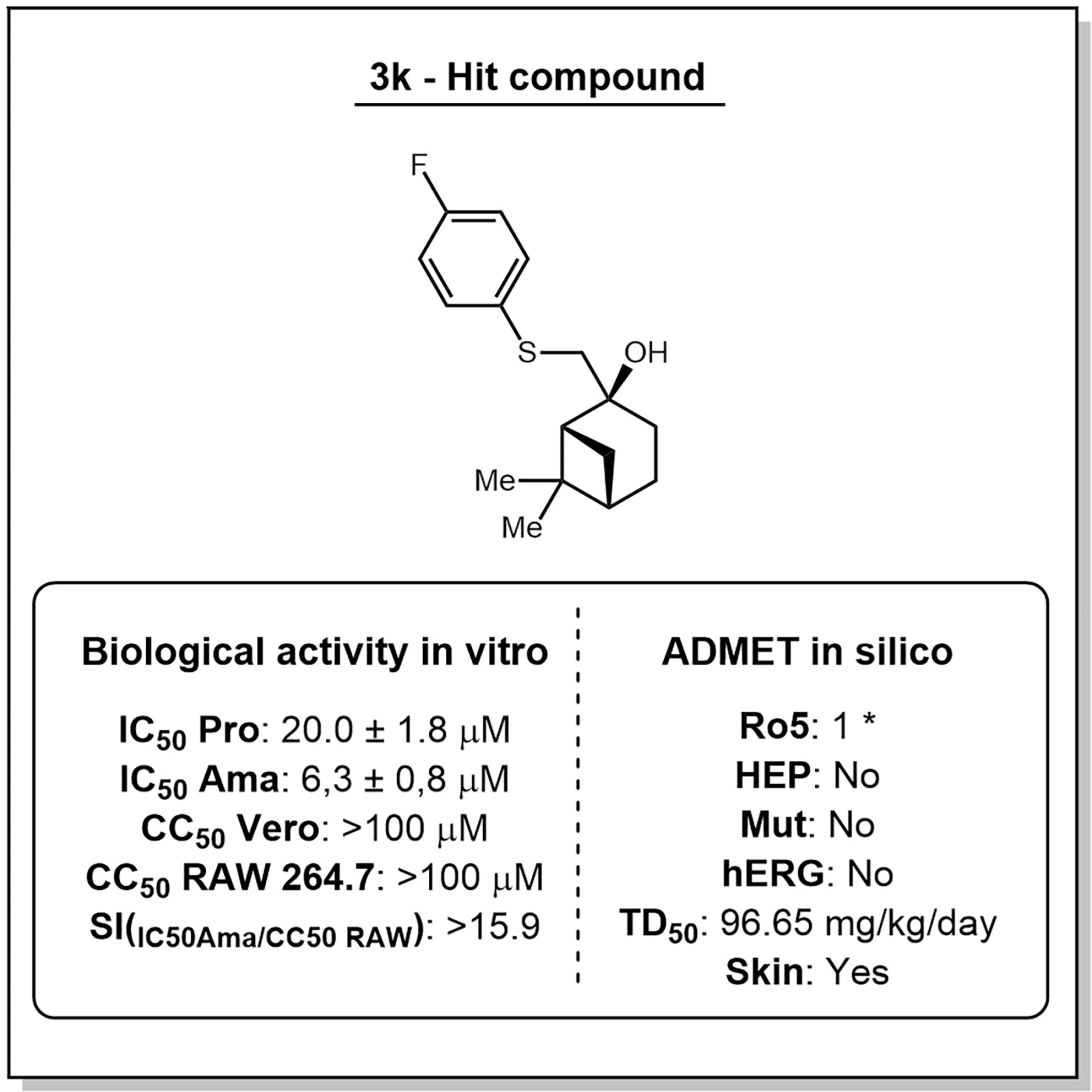
Experimental *in vitro* activity of **3k** and *in silico* prediction pharmacokinetic and toxicity screening Lipinski’s “rules of five” analysis parameters and specific toxicity analysis (Ro5), hepatotoxicity, mutagenicity, cardiotoxicity (hERG), carcinogenicity in rodents, and skin sensitization.

Taken together, these features prompted us to discuss plausible mechanisms of action. Although the precise molecular targets of our β-pinene hydroxysulfide derivatives remain to be experimentally validated, converging evidence of structurally related sulfur-containing compounds supports plausible hypotheses for future investigation. The trypanothione/trypanothione reductase (TR) system is a well-validated drug target in *Leishmania* spp. and other trypanosomatids^6,7^ Diaryl sulfide compounds have been extensively studied as TR inhibitors, with X-ray crystallographic studies revealing that the sulfur linker positions aryl substituents optimally within the TR active site, enabling key interactions with catalytic residues such as Glu466′, Cys57, and Cys52.^9^ For example, the diaryl sulfide RDS 777 exhibits competitive inhibition of *L. infantum* TR with a *K*_i_ of approximately 0.25 μM and demonstrates whole-cell antileishmanial activity (IC_50_ ≈ 29.4 μM).^9^ Structure-guided optimization of this scaffold yielded derivatives that reduced intracellular trypanothione levels by ∼33% in treated parasites, directly linking TR inhibition to cellular effects.^10^

Our β-pinene hydroxysulfide derivatives share key structural features with these established TR inhibitors: a thioether linkage and (hetero)­aryl substituents that could potentially engage the TR active site. The enhanced activity of the *para*-fluoroaryl analogue (IC_50_ = 6.3 μM against intracellular amastigotes, SI > 15.9) is particularly noteworthy, as halogenated aryl groups have been shown to improve TR binding affinity and whole-cell potency in related series.^9,10^ The fluorine atom may enhance metabolic stability, optimize lipophilicity for membrane penetration, and provide favorable electronic effects for target binding. Furthermore, the lipophilic β-pinene scaffold may facilitate membrane permeability and intracellular accumulation, enabling access to the cytoplasmic TR target.

Our SAR analysis revealed that lipophilicity, polar surface area (PSA), and cLogP are critical determinants of antileishmanial activity. These physicochemical properties are directly relevant to the proposed mechanism, as compounds must traverse multiple membrane barriers to reach intracellular targets: the macrophage plasma membrane, the parasitophorous vacuole membrane, and the parasite plasma membrane. The optimal c Log P range observed in our series is consistent with the need for balanced lipophilicity to achieve favorable intracellular accumulation while maintaining sufficient aqueous solubility.^41^ The β-pinene scaffold provides a rigid, lipophilic framework that may facilitate membrane insertion and intracellular delivery of the pharmacophoric sulfide moiety.

The superior activity of the *para*-fluoroaryl derivative can be rationalized by fluorine’s unique properties in medicinal chemistry: increased metabolic stability, enhanced lipophilicity without excessive molecular weight, and favorable electronic effects that may strengthen target binding. Aryl substituents in our series likely modulate both physicochemical properties (affecting membrane permeability) and molecular recognition (affecting potential TR binding affinity), consistent with the dual role of aryl groups in reported diaryl sulfide TR inhibitors.^9,10^

To contextualize our findings, we note that our hit compound’s activity profile (IC_50_ = 6.3 μM, SI > 15.9) is comparable to miltefosine, a clinically used antileishmanial agent that typically exhibits IC_50_ values of 2–10 μM against *L. amazonensis* amastigotes.^42^ The comparable potency and favorable selectivity index support the potential of our hit compound as a lead structure for further optimization.

## Conclusion

In conclusion, we demonstrated that (−)-β-pinene epoxide is a versatile chiral building block for the construction of novel derivatives, enabling the synthesis of β-hydroxysulfides bearing a quaternary stereogenic center. These compounds displayed significant activity against *L. amazonensis*, particularly toward the intracellular amastigote, which is the clinically relevant form in the mammalian host. Among them, the most active derivative (**3k**) stood out for its lipophilic character, likely attributed to the bioisosteric incorporation of fluorine into the aromatic ring, which enhanced lipophilicity and strengthened interactions with potential molecular targets in the parasite. Structure–activity relationship analysis revealed key physicochemical features, molecular volume (MV), polar surface area (PSA), and cLogP, which are strongly associated with the observed bioactivity. Complementary *in silico* ADMET studies supported compound **3k** as a promising hit candidate, exhibiting favorable oral bioavailability and compliance with international drug discovery guidelines for leishmaniasis.

The structural features of our β-pinene hydroxysulfide derivatives, combined with their activity profile and SAR trends, suggest potential targeting of the parasite-specific trypanothione reductase system and induction of oxidative stress, mechanisms well-established for structurally related sulfur-containing antileishmanial agents. Future mechanistic studies, including enzyme inhibition assays, ROS quantification, and molecular modeling, will be essential to validate these hypotheses and guide rational optimization of this promising scaffold.

As future work, we intend to advance to *in vivo* studies to assess the efficacy and safety of the most active derivatives in animal models as well as to investigate their mechanism of action at the molecular level.

## Experimental Section

### Chemical Synthesis

#### Procedure for β-Pinene Epoxidation^23^

In a 125 mL Erlenmeyer flask, (−)-β-pinene (1.360 g, 10 mmol, 1 equiv) and sodium bicarbonate (4.0324 g, 23.9 mmol, 4.8 equiv) were dissolved in 40 mL of acetone. An aqueous solution of Oxone (4.0192 g, 10 mmol, 1 equiv, in 60 mL of deionized water) was then added dropwise over 3 min. The reaction mixture was stirred vigorously at room temperature for 30 min and then extracted with dichloromethane (3 × 30 mL). The combined organic layers were dried over anhydrous MgSO_4_, filtered, and concentrated under reduced pressure. The crude product was purified by column chromatography on silica gel pretreated with 2.5% triethylamine in *n*-hexane, eluting with 1% ethyl acetate/hexane, to afford compound **2** as a colorless oil in 44% yield.

#### General Procedure for β-Pinene Epoxide Thiolysis

The thiolysis reaction for the synthesis of β-hydroxysulfide derivatives **3a–p** was performed in a conical vial under an argon atmosphere. Anhydrous methanol (1.2 mL) and sodium (11 mg, 0.5 mmol, 1.6 equiv) were stirred in an ice–water bath until complete dissolution, followed by the addition of the corresponding thiol (0.5 mmol, 1.6 equiv). The mixture was then heated at 65 °C for 10 min, after which compound **2** (50 mg, 54 μL, 0.32 mmol, 1 equiv) was added, and the reaction was stirred for 6 h. The reaction was quenched with saturated aqueous NH_4_Cl (10 mL), and the methanol was removed under reduced pressure. The aqueous phase was extracted with ethyl acetate (3 × 20 mL), and the combined organic layers were dried over anhydrous MgSO_4_ and concentrated. The crude product was purified by column chromatography to afford the desired derivatives.

#### Biological Evaluation

##### Parasites

*Leishmania amazonensis* (MHOM/BR/77/LTB0016) was maintained as promastigotes at 26 ◦C in Schneider’s insect medium (Sigma-Aldrich, St Louis, MO, USA) with 10% heat-inactivated fetal calf serum (HIFCS), 100 μg/mL streptomycin, and 100 U/mL penicillin. Parasites were maintained until the 10th passage; subsequently, new cultures were obtained from infected animals.

##### Antipromastigote Activity

*L. amazonensis* promastigotes were cultivated in Schneider’s insect medium supplemented with 10% HIFCS as above, in either the absence or presence of different concentrations of the substances. The culture was initiated with 1.0 × 106 cells·mL^‑1^ and maintained at 26 °C for 72 h. Cell viability was estimated by reducing the level of resazurin.

##### Antiamastigote Activity

Resident peritoneal macrophages were plated in RPMI medium (Sigma-Aldrich) at 1 × 106/mL (0.4 mL/well) in Lab-Tek eight-chamber slides (Nunc) and incubated at 37 ◦C in 5% CO_2_ for 1 h. After 1 h, the medium containing nonadherent cells was removed. The remaining cells were incubated at 37 ◦C and 5% CO_2_ with *L. amazonensis* promastigotes in a ratio of 5:1. After 4 h, the monolayers were washed to remove the free parasites. The cells were incubated with the substances in different concentrations for 72 h at 37 ◦C and 5% CO_2_. After the incubation period, the slides were stained using the Instant Prov hematological dye system (Newprov, Curitiba, Brazil), and leishmanicidal activity was evaluated microscopically. The number of amastigotes was determined by counting at least 100 macrophages per sample. The results are expressed as an infection index (II), which was calculated as follows:

II=(percentageofinfectedcells)×(number⁢ofamastigotes/totalnumberofmacrophages)

#### IC_50_ Determination and Statistical Analysis

Concentration–response curves were generated from at least three independent experiments performed in triplicate (*n* ≥ 3). Data were analyzed using GraphPad Prism software (ver. 10.6.1, GraphPad Software, San Diego, CA, USA) by nonlinear regression analysis. The four-parameter logistic model (4PL; “sigmoidal dose–response, variable slope”) was fitted to the normalized data:

Y=bottom+(top−bottom)/[1+10^((LogIC50−X)×hillslope)]

where *Y* is the percent inhibition, *X* is the log_10_ of compound concentration, and bottom and top were constrained to 0 and 100%, respectively.

IC_50_ values were determined as the concentration producing 50% inhibition and are reported as the mean ± standard error of the mean (SEM) from independent experiments. The 95% confidence intervals (CI) were calculated from the asymptotic standard errors of the nonlinear least-squares fit. The goodness-of-fit was assessed by *R* ^2^ values (all >0.95) and visual inspection of residual plots. No comparisons between IC_50_ values of the different compounds were performed.

#### Cell Viability and Selectivity Index Calculation

RAW 264.7 macrophage and VERO cell lines were maintained in complete DMEM medium (supplemented with 10% fetal bovine serum and streptomycin 100 μg/mL to penicillin 100 U/mL) and maintained at 37 °C in a 5% CO_2_ atmosphere. For the cytotoxicity assays, the cells were harvested at subconfluency (after 48 h of growth), washed twice with phosphate-buffered saline (PBS, pH 7.2), detached with 0.25% trypsin solution, and resuspended in fresh medium. Subsequently, 10^5^ cells were seeded into 96-well microplates and incubated under the same conditions for 2 h (RAW = 264.7) or 24 h (VERO) to allow adherence. The cultures were then treated with different concentrations of derivatives **3a–3p** (3.12–100 μM) for 48 h. After treatment, MTT solution (0.5 mg/mL) was added, and cells were incubated for 3 h. The supernatant was removed, 100 μL of dimethyl sulfoxide (DMSO) was added to dissolve the formazan crystals, and absorbance was measured at 570 nm (SpectraMax M2, Molecular Devices, CA, USA). The half-maximal cytotoxic concentration (CC_50_) was determined by nonlinear regression analysis. The selectivity index (SI) was calculated as the ratio of CC_50_ (RAW = 264.7) to IC_50_.

#### In Silico

##### Molecular Modeling

The 3D chemical structures of the β-pinene derivatives 3a-p were constructed, and the molecular modeling calculations were performed using SPARTAN’10 software (Wave function Inc., CA, 2000). The compounds in their neutral states were submitted to a systematic search using a molecular mechanics calculation (MMFF94Aq) to obtain conformations of the minimum energy state. The geometry optimization of these derivatives was subsequently performed in vacuum, using the semiempirical method RM1. In order to obtain the molecular descriptors, all of the derivatives were submitted to a single-point calculation using the DFT method with the B3LYP/6–311G* basis set. Some molecular descriptors were obtained for these compounds, such as the highest occupied molecular orbital (HOMO) and the lowest unoccupied molecular orbital (LUMO) energy values, orbital coefficient and density, molecular volume and electrostatic potential maps (MEPs), dipole moment, and the partial atomic charges. The 3D isosurfaces of the MEPs at the van der Waals contact surface represent electrostatic potentials superimposed onto a surface of constant electron density (0.002 e/au3) and were generated at a range of −150 to +150 kJ/mol. These color-coded isosurface values indicate the overall molecular size and location of negative (red) or positive (blue) electrostatic potentials.

##### *In Silico* Pharmacokinetic and Toxicology Profile

The 2D chemical structure of the most active β-pinene derivative (3k), along with miltefosine, was subjected to in silico ADMET analysis (absorption, distribution, metabolism, excretion, and toxicity) using QSAR-based models implemented in the ADMET Predictor version 11 software (Simulation Plus Inc., Lancaster, CA, USA). Toxicological parameters predicted included hepatotoxicity, mutagenicity, cardiotoxicity, carcinogenicity, and skin sensitization. Hepatotoxicity predictions were based on the potential elevation of five key serum biomarkers: alkaline phosphatase (ALP), aspartate transaminase (AST), alanine transaminase (ALT), γ-glutamyltransferase (GGT), and lactate dehydrogenase (LDH). Mutagenic potential was assessed using a model based on the Ames test, while cardiotoxicity was predicted through the likelihood of hERG potassium channel inhibition, which is associated with arrhythmogenic risk. Finally, a qualitative assessment of allergenic skin sensitization was performed based on the murine local lymph node assay (LLNA), a validated method for the determination of the relative potency of skin sensitizing chemicals.

## Supplementary Material



## Notes

General information; (−)-β-pinene epoxidation optimization; thiolysis reaction optimization; synthesis of β-hydroxysulfides 3a–3p; ^1^H and ^13^C NMR of β-pinene epoxide (2) and β-hydroxysulfides derivatives 3a–3p; biological evaluation: determination of IC_50_ against promastigote and intracellular amastigote of *Leishmania amazonensis*; antipromastigote activity of 3a–p at fixed concentration of 100 μM; determination of IC_50_ against intracellular amastigote *L. amazonensis* 7; and references (PDF)
