# Supplementary material for: Hit-Identification to Novel Antileishmanial Agents from a β‑Pinene Scaffold: from Synthesis to In Vitro Evaluation and In Silico SAR/ADMET Profiling
Source: ACS Omega. 2026 Jan 20;11(4):5498–507. doi: 10.1021/acsomega.5c08971 (PMC12878726; doi:10.1021/acsomega.5c08971)
Supplement: Supplementary file 1 [file ao5c08971_si_001.pdf]

Supporting Information for:

Hit-Identification to Novel Antileishmanial Agents from a β-Pinene Scaffold: From synthesis to In Vitro Evaluation and In Silico SAR/ADMET profiling

Gustavo dos S. Martins,[1,2] Bruno M. da S. Santos,[2] Yago S. S. Emiliano,[3] João Pedro A. Santos,[4] Gérzia M. Machado,[3] Mariana S. de Carvalho,[3] Kamila Marques Sette,[5] Igor de A. Rodrigues,[5] Alessandra M. T. de Souza,[4] Eduardo Caio Torres-Santos[3], Fernanda G. Finelli,[2] Ivana Correa Ramos Leal[1]*

[1] Laboratory of Natural Products and Biological Assays (LaProNEB), Department of Natural Products and Food, Faculty of Pharmacy, Federal University of Rio de Janeiro, UFRJ, [2] Laboratory of Organic Synthesis, Instituto de Pesquisa de Produtos Naturais, Federal University of Rio de Janeiro, UFRJ, [3] Laboratory of Trypanosomatid Biochemistry, Instituto Oswaldo Cruz, FIOCRUZ, 4 Laboratory of Molecular Modelling QSAR, Faculty of Pharmacy, Federal University of Rio de Janeiro, UFRJ, 5 Laboratory of Investigation of Bioactive Substances, Department of Natural Products and Food, Faculty of Pharmacy, Federal University of Rio de Janeiro, UFRJ

**Corresponding author: ivanafarma@yahoo.com.br; ivana@farmacia.ufrj.br*

Table of contents

1. General 2
2. (-)-β-pinene epoxidation optimization 3
3. Thiolysis reaction optimization...4
4. Synthesis of β-hydroxysulfides **3a-p** 5
5. ^{1}H and ^{13}C NMR of β-pinene epoxide (**2**) and β-hydroxysulfides derivatives 3**a-p**........13
6. Biological evaluation: determination of IC_{50} against promastigote and intracellular amastigote of *Leishmania amazonensis* 42
6.1. Antipromastigote activity of 3**a-p** at fixed concentration of 100 μM......................42
7. References 44

1. General

Commercially available reagents and solvents were purified and dried according to procedures described in the literature. Anhydrous methanol (MeOH) was obtained by treatment with calcium hydride (CaH_2) under argon atmosphere and stirring overnight, followed by distillation and storage in a sealed flask for up to one week. Ethyl acetate and hexane were used after distillation. All other reagents and solvents were used without further purification. Flash column chromatography with silica gel (200–400 mesh) as the stationary phase was used for compound purification. The eluents employed as the mobile phase are described in the experimental procedures.

Proton and carbon nuclear magnetic resonance (^1H and ^{13}C NMR) spectra were recorded on Varian MR-400 (400 MHz) and VNMRSYS-500 (500 MHz) spectrometers. Chemical shifts (δ) are reported in parts per million (ppm), and coupling constants (J) are given in Hertz (Hz). NMR data were described using the following abbreviations: s (singlet), brs (broad singlet), d (doublet), t (triplet), q (quartet), quint (quintet), dd (doublet of doublets), ddd (doublet of doublets of doublets), dt (doublet of triplets), and m (multiplet). High-resolution mass spectra (HRMS) were obtained using a Bruker Daltonics solariX FT-ICR mass spectrometer by direct infusion and electrospray ionization (ESI) in positive mode.Infrared spectra were collected on a Thermo FTIR iS-50 equipped with an ATR probe.

2. (-)-β-pinene epoxidation optimization

Table S1. Synthetic procedures for obtention of β-pinene epoxide (**2**)

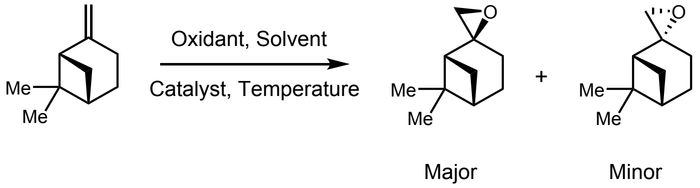

Entry	Reaction condition	Yield (%)	d.r[a]
1	1 eq. of OXONE, r.t, 30 min [b]	44%[c]	12:1
**2**	2 eq. of OXONE, r.t, 30 min [b]	39%[d]	12:1
**3**	8.8 eq. of H_2O_2, 2 eq. of DCC, 2 eq. of $KHCO_2$, r.t, 24 h[e]	58%[d]	>95:5
**4**	2.5 eq. of UHP*, 10% m/v of Novozyme 435 [e]	68%[c]	3:1
**5**	2.5 eq. of UHP*, 10% m/v of Novozyme 435, 0.1 eq. of $NaHCO_3$ [e]	70%[c]	6:1
**6**	2.5 eq. of UHP*, 10% m/v of Novozyme 435, 1 eq. of $NaHCO_3$ [e]	61%[c]	5:1
**7**	2.5 eq. of UHP*, 10% m/v of Novozyme 435, 1 eq. of K_2HPO4[e]	54%[c]	3:1
**8**	2.5 eq. of UHP*, 10% m/v of Novozyme 435, 1 eq. of Et_3N[e]	-	-

[a] Calculated based on the ratio of the signals in δ2,77 ppm and δ2,55 ppm in ^{1}H NMR analysis; [b] Reaction conducted using 5 mmol of (-)-β-pinene and 4.8 equivalent of $NaHCO_3$; [c]Isolated yield; [d] Yield by 1H NMR analysis using 1,3-benzodioxole as internal standard; [e] Reaction conducted using 1 mmol of (-)-β-pinene, 0.33 M of ethyl acetate, molecular sieves 4Å, 45 °C, 225 rpm, 3 h. *Urea-Hydrogen Peroxide adduct.

(1R,2S,5S)-6,6-dimethylspiro[bicyclo[3.1.1]heptane-2,2'-oxirane] (2):

^{1}H NMR (500 MHz, $CDCl_3$) (major diastereoisomer): δ 2.77 (d, J = 4.8 Hz, 1H), 2.61 (d, J = 4.8 Hz, 1H), 2.37 – 2.23 (m, 2H), 2.22 – 2.11 (m, 2H), 2.04 – 1.94 (m, 1H), 1.94 – 1.76 (m, 3H), 1.75 – 1.63 (m, 3H), 1.60 – 1.48 (m, 1H), 1.25 (s, 3H), 0.92 (s, 3H).

^{13}C NMR (126 MHz, $CDCl_3$) δ 61.63, 56.58, 49.04, 40.87, 40.29, 26.24, 25.31, 23.70, 22.45, 21.31.

HRMS (ESI): Calculated for $C_{10}H_{17}O$ $[M + H]^+$: m/z 153.1274; found: m/z 153.1274. Error: 0 ppm.

3. Thiolysis reaction optimization

Table S2. Reaction optimization under basic conditions using thiophenol as model nucleophile.

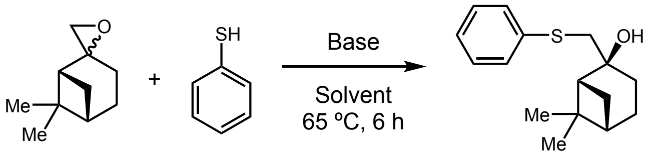

Entry	Reaction Conditions [a,e]	Yield (%)[f]
1	NaOH (2 equiv.), Methanol	65[b]
2	NaOH (4 equiv.), Methanol	52[b]
3	NaOH (2 equiv.), Acetonitrile	58[b]
4	NaOH (2 equiv.), Acetonitrile:Water (1:1)	48[b]
5	NaOH (2 equiv.), Methanol[c]	64[b]
6	KOH (2 equiv.), Methanol	60[b]
7	KHCO3 (2 equiv.), Methanol	60[b]
8	DBU (2 equiv.), DMSO	58[b]
9	DBU (3.2 equiv.), DMSO	46[b]
10	Pirrolidine (3.2 equiv.), DMSO	26[b]
11	NaOMe (1.6 equiv.), Methanol	81[d]

[a] Reaction conducted in 0.32 mmol of **2**(50 mg, 54 μL), 0.5 mmol of thiophenol (1.6 equiv., 55 mg, 59 μL), 0.26 M of solvent, 65 °C, 6 h; [b] Yield determined by ^{1}H NMR using 1,3-benzodioxole as internal standard; [c] Reaction conducted using 0.52 M of solvent; [d] Isolated yield. [e]**2** was used as a mixture of diastereoisomers in the ratio of 12:1 (anti:syn); [f] Yield reported of the major diastereoisomer.

4. Synthesis of β-hydroxysulfides 3a-p

*(1R,2S,5S)-6,6-dimethyl-2-((phenylthio)methyl)bicyclo[3.1.1]heptan-2-ol (**3a**)*:

Synthesized according to the general procedure of thiolysis, using thiophenol (0.5 mmol, 1.6 equiv., 63.9 mg, 59 μL). The product was isolated by column chromatography on an Isolera One system, using a 0–20% ethyl acetate/n-hexane gradient, affording compound **3a** as a yellowish oil (68 mg, 81% yield).

^{1}H NMR (500 MHz. CDCl$_3$) δ 7.43 (dd, *J* = 8.3. 1.2 Hz. 2H), 7.28 (t. *J* = 7.5 Hz. 2H). 7.21 – 7.17 (m. 1H), 3.30 (d. *J* = 13.4 Hz. 1H), 3.22 (d. *J* = 13.4 Hz. 1H), 2.45 (s. 1H), 2.18 (dtd. *J* = 10.1. 6.2. 2.1 Hz. 1H), 2.06 (dd. *J* = 6.3. 4.9 Hz. 1H), 1.99 – 1.80 (m. 5H), 1.54 (d. *J* = 10.1 Hz. 1H), 1.12 (s. 3H), 0.90 (s. 3H) ppm.

^{13}C NMR (126 MHz. CDCl$_3$)δ 136.74, 130.54, 129.11, 126.61, 76.28, 51.56, 48.42, 41.38, 37.49, 29.96, 27.37, 27.31, 25.00, 22.68 ppm.

HRMS (ESI): Calculated for $C_{16}H_{22}OSNa$ [M + Na]$^+$: m/z 285.1284; found: m/z 285.1283. Error: −0.1 ppm.

IR (KBr): ν˜ (cm^{-1}): 3452 (br) (O-H), 2910 (m) (C-H), 2866 (s) (C-H sp^2), 1583 (m) (C=C), 1087 (s) (C-O).

(1R,2S,5S)-2-(((2-hydroxyethyl)thio)methyl)-6,6-dimethylbicyclo[3.1.1]heptan-2-ol (**3b**):

Synthesized according to the general procedure of thiolysis, using 2-mercaptoethanol (0.5 mmol, 1.6 equiv.v, 57 mg, 57 μL). The product was isolated by column chromatography on an Isolera One system, using a 25–32% ethyl acetate/n-hexane gradient, affording compound **3b** as a yellowish oil (67.3 mg, 91% yield).

^{1}H NMR (500 MHz, CDCl$_3$): δ 3.81–3.73 (m, 2H), 2.89 (d, *J* = 13.5 Hz, 1H), 2.82 (d, *J* = 13.6 Hz, 1H), 2.79–2.75 (m, 2H), 2.21 (dtd, *J* = 10.2, 6.1, 2.0 Hz, 1H), 2.07 (dd, *J* = 6.2, 4.8 Hz, 1H), 1.99–1.78 (m, 5H), 1.51 (d, J = 10.1 Hz, 1H), 1.25 (s, 3H), 0.95 (s, 3H) ppm.

^{13}C NMR (126 MHz, CDCl$_3$): δ 76.29, 61.56, 50.82, 47.01, 40.97, 38.34, 37.45, 31.18, 28.07, 27.32, 24.99, 23.57 ppm.

HRMS (ESI): Calculated for $C_{12}H_{22}NaO_2S$ [M+Na]$^+$ m/z 252.1233; found m/z 253.1218. Error: −6.0 ppm.

IR (KBr): ν˜ (cm^{-1}): 3019 (br) (3019), 1214 (m) (C-O).

(1R,2S,5S)-2-(((4-chlorophenyl)thio)methil)-6,6-dimethilbicyclo[3.1.1]heptan-2-ol (***3c***): Synthesized according to the general procedure of thiolysis, using 4-chlorothiophenol (0.5 mmol, 1.6 equiv., 74 mg). The product was isolated by column chromatography on an Isolera One system, using 5% ethyl acetate/n-hexane, affording compound **3c** as a yellowish oil (80.7 mg, 85% yield).

^{1}H NMR (500 MHz, CDCl$_3$)δ 7.37 – 7.32 (m, 2H), 7.27 – 7.21 (m, 2H), 3.25 (d, *J* = 13.3 Hz, 1H), 3.19 (d, *J* = 13.3 Hz, 1H), 2.33 (s, 1H), 2.18 (dtd, *J* =.10.2, 6.1, 2.0 Hz, 1H), 2.04 (dd, *J* = 6.3, 4.8 Hz, 1H), 1.99 – 1.74 (m, 5H), 1.52 (d, *J* = 10.2 Hz, 1H), 1.15 (s, 3H), 0.90 (s, 3H) ppm.

^{13}C NMR (126 MHz, CDCl$_3$)δ 135.85, 132.50, 131.64, 129.17, 76.25, 51.95, 49.25, 41.47, 37.60, 29.41, 28.10, 27.30, 25.46, 23.43 ppm.

HRMS (ESI): Calculated for $C_{16}H_{20}ClS$ [M-H_2O]$^+$ m/z 279.0969; found m/z 279.0984. Error: −5.4 ppm.

IR (KBr): ν˜ (cm^{-1}): 3382 (br) (O-H), 2912 (m) (C-H), 2867 (m) (C-H sp^2), 1573 (w) (C=C), 1090 (C-O), 741 (m) (C-Cl)

*(1R,2S,5S)-2-((benzylthio)methil)-6,6-dimethilbicyclo[3.1.1]heptan-2-ol (**3d**):*

Synthesis according to the general procedure of thiolysis, using benzylmercaptan (0,5 mmol, 1,6 equiv., 63,3 mg, 60 μL. The product was isolated by column chromatography on an Isolera One system, using a 2-10% ethyl acetate/n-hexane gradient, affording compound **3d** as a yellowish oil (73.1 mg, 85% yield).

^{1}H NMR(500 MHz, CDCl$_3$) δ 7.37 – 7.27 (m, 4H), 7.24 (tt, *J* = 5,6, 3,0 Hz, 1H), 3.76 (s, 2H), 2.77 (d, *J* = 13.3 Hz, 1H), 2.72 (d, *J* = 13.4 Hz, 1H), 2.35 (*s*, 1H), 2.18 (dtd, *J* = 10.1, 6.1, 1.9 Hz, 1H), 2.03 (dd, *J* = 6.3, 4.8 Hz, 1H), 1.96 – 1.73 (m, 5H), 1.51 (d, *J* = 10.1 Hz, 1H), 1.22 (*s*, 3H), 0.84 (s, 3H) ppm.

^{13}C NMR (126 MHz, CDCl$_3$)δ 138.50, 129.07, 128.69, 127.26, 75.52, 51.50, 45.75, 40.96, 38.31, 38.29, 30.59, 27.6, 27.32, 25.01, 23.40 ppm.

HRMS (ESI): Calculated for $C_{17}H_{23}S$ *m/z* 259.1515 [M-H_2O]$^+$, found *m/z* 259.1531. Error: 6,1 ppm.

IR (KBr): ν˜ (cm^{-1}): 3461 (br) (O-H), 2909 (m) (C-H), 2866 (m) (C-H sp^2), 1601 (w) (C=C), 1023 (m) (C-O).

(*1R,2S,5S)-6,6-dimethyl-2-((p-toluilthio)methyl)bicyclo[3.1.1]heptan-2-ol* (**3e**):

Synthesis according to the general procedure of thiolysis, using 4-methylthiophenol (0,5 mmol, 1,6 eq., 62 mg, 61 µL). The product was isolated by column chromatography on an Isolera One system, using 1-5% ethyl acetate-hexane gradient, affording **3e** as a white solid (63.4 mg, 72% yield).

^{1}H NMR (500 MHz, $CDCl_3$)δ 7.35 – 7.31 (m, 2H), 7.11 – 7.07 (m, 2H), 3.26 (d, *J* = 13.4 Hz, 1H), 3.17 (d, *J* = 13.4 Hz, 1H), 2.31 (s, 3H), 2.16 (dtd, *J* = 10.2, 6.1, 2.0 Hz, 1H), 2.08 – 2.03 (m, 1H), 1.95 – 1.78 (m, 5H), 1.53 (d, *J* = 10.1 Hz, 1H), 1,11 (s. 3H), 0,89 (s, 3H) ppm,

^{13}C NMR (126 MHz, $CDCl_3$)δ 137.26, 133.39, 131.10, 129.90, 76.28, 51.14, 49.74, 41.01, 38.25, 31.16, 28.10, 27.29, 25.00, 23.37, 21.15 ppm.

HRMS (ESI): Calculated for $C_{17}H_{23}S$ m/z 259.1515 [M-H_2O]+, found m/z 259.1515. Error: 0 ppm

IR (KBr): ν˜ (cm^{-1}): 3019 (br) (O-H), 2924 (m) (C-H), 2833 (m) (C-H sp^2), 1599 (w) (C=C), 1030 (m) (C-O)

(1R,2S,5S)-2-(((4-aminophenyl)thio)methyl)-6,6-dimethylbicyclo[3.1.1]heptan-2-ol (***3f***):

Synthesis according to the general prodedure of thiolysis, using 4-aminothiophenol (0,5 mmol, 1,6 equiv., 63 mg, 56 µL). In this reaction, the aqueous solution of NH_4Cl was substituted for phosphate buffer (pH 9-10). The product was isolated by column chromatography on Isolera One system, using 10-20% of ethyl acetate-hexane gradient, affording **3f** as a yellowish oil (63 mg, 71% yield).

1H NMR (500 MHz, $CDCl_3$)δ 7.28 – 7.25 (m, 2H), 6.61 – 6.58 (m, 2H), 3.70 (s, 2H), 3.17 (d, *J* = 13.5 Hz, 1H), 3.08 (d, *J* = 13.5 Hz, 1H), 2.55 (s, 1H), 2.15 (dtd, *J* = 10.0, 6.1, 1.8 Hz, 1H), 2.07 – 2.03 (m, 1H), 1.94 – 1.73 (m, 5H), 1.51 (d, *J* = 10.0 Hz, 1H), 1.09 (s, 3H), 0.83 (s, 3H) ppm.

^{13}C NMR (126 MHz, $CDCl_3$)δ 146.15, 134.22, 124.28, 117.13, 76.37, 51.38, 51.00, 40.88, 38.18, 30.45, 27.35, 27.24, 25.00, 23.36, ppm.

HRMS (ESI): Calculated for $C_{16}H_{22}NS$ *m/z* 260.1467 [M-H_2O]$^+$, found *m/z* 260.1484. Error: 6.2 ppm.

IR (KBr): ν˜ (cm^{-1}): 3433 (s) (N-H), 3033(br) (O-H), 2900 (m) (C-H), 2824 (m) (C-H sp^2), 1599 (w) (C=C), 1244 (C-N sp^2) 1025 (m) (C-O).

(1R,2S,5S)-6,6-dimethyl-2-((o-tolylthio)methyl)bicyclo[3.1.1]heptan-2-ol (***3g***)

Synthesis according to the general prodedure of thiolysis, using 2-methylthiophenol (0,5 mmol, 1,6 equiv., 61 mg, 60 µL). The product was isolated by column chromatography on

Isolera One system, using 1-5% of ethyl acetate-hexane gradient, affording **3g** as a white solid (51.1 mg, 58% yield).

^{1}H NMR (500 MHz, $CDCl_3$)δ 7.41 (dd, *J* = 7.5, 1.6 Hz, 1H), 7.19 – 7.07 (m, 3H), 3.24 (d, *J* = 13.1 Hz, 1H), 3.18 (d, *J* = 13.1 Hz, 1H), 2.43 (s, 3H), 2.41 (s, 1H), 2.19 (dtd, *J* = 10.1, 6.1, 2.0 Hz, 1H), 2.07 (dd, *J* = 6.3, 4.8 Hz, 1H), 1.99 – 1.88 (m, 4H), 1.81 (dddt, *J* = 10.5, 5.0, 4.2, 2.6 Hz, 1H), 1.54 (d, *J* = 10.1 Hz, 1H), 1.15 (s, 3H), 0.91 (s, 3H) ppm.

^{13}C NMR (126 MHz, $CDCl_3$)δ 138.46, 136.14, 130.35, 130.14, 126.64, 126.49, 76.22, 51.33, 48.31, 40.90, 38.24, 30.51, 27.42, 27.31, 24.99, 23.35, 20.85 ppm.

HRMS (ESI): Calculated for $C_{17}H_{23}SK$ m/z 297.1074 $[M\text{-}H_2O+K]^+$, found m/z 297.1074. Error: 0.1 ppm.

IR (KBr): ν˜ (cm^{-1}): 3028 (br) (O-H), 2912 (m) (C-H), 2830 (m) (C-H sp^2), 1602 (w) (C=C), 1027 (m) (C-O)

(1R,2S,5S)-2-(((furan-2-ylmethyl)thio)methyl)-6,6-dimethylbicyclo[3.1.1]heptan-2-ol (***3h***)

Synthesis according to the general procedure of thiolysis, using furan-2-ylmethanethiol (0.5 mmol, 1.6 equiv., 57 mg, 57 μL). The product was isolated by column chromatography on Isolera One system, using 2-20% of ethyl acetate-heane, affording **3h** as a yellowish oild (55.5 mg, 65% yield).

^{1}H NMR (500 MHz, $CDCl_3$) δ 7.35 (dd, *J* = 1.9, 0.8 Hz, 1H), 6.30 (dd, *J* = 3.2, 1.9 Hz, 1H), 6.19 (dd, *J* = 3.2, 0.8 Hz, 1H), 3.76 (s, 2H), 2.84 (d, *J* = 13.5 Hz, 1H), 2.78 (d, *J* = 13.5 Hz, 1H), 2.18 (dtd, J = 10.0, 6.1, 1.9 Hz, 1H), 2.02 (dd, *J* = 6.3, 4.8 Hz, 1H), 1.97 – 1.74 (m, 5H), 1.51 (d, J = 10.1 Hz, 1H), 1.22 (s, 3H), 0.90 (s, 3H) ppm.

^{13}C NMR (126 MHz, $CDCl_3$) δ 152.26, 142.23, 110.41, 108.43, 76.05, 51.39, 45.78, 40.83, 38.16, 30.42, 29.50, 27.96, 27.19, 24.87, 23.30 ppm.

HRMS (ESI): Calculated for $C_{15}H_{21}OS$ *m/z* 249.1308 $[M\text{-}H_2O]^+$, found *m/z* 249.1293. Error: -6.0 ppm.

IR (KBr): ν˜ (cm^{-1}): 3340 (br) (O-H), 2944 (m) (C-H), 2876 (m) (C-H sp^2), 1633 (w) (C=C),1055 (m) (C-O).

(1R,2S,5S)-2-(((4-metoxyphenyl)thio)methyl)-6,6-dimethyllbicyclo[3.1.1]heptan-2-ol (***3i***)

Synthesis according to the general procedure of thiolysis, using 4-metoxythiophenol (0.5 mmol, 1.6 equiv., 70 mg, 62 μL). The product was isolated by column chromatography using Isolera One system, using 2-10% of ethyl acetate-hexane gradient, affording **3i** as a yellowish oil (58.8 mg, 63% of yield).

^{1}H NMR (500 MHz, $CDCl_3$) δ 7.42 – 7.37 (m, 2H), 6.85 – 6.81 (m, 2H), 3.79 (s, 3H), 3.20 (s, 1H), 3.13 (d, *J* = 13.5 Hz, 1H), 2.49 (s, 1H), 2.16 (dtd, *J* = 10.1, 6.1, 1.9 Hz, 1H), 2.05 (dd, *J* = 6.3, 4.9 Hz, 1H), 1.96 – 1.75 (m, 5H), 1.52 (d, *J* = 10.1 Hz, 1H), 1.10 (s, 3H), 0.85 (s, 3H) ppm.

^{13}C NMR (126 MHz, $CDCl_3$) δ 158.47, 134.18, 128.06, 114.81, 75.09, 54.42, 51.07, 50.96, 40.90, 38.72, 30.47, 27.38, 27.27, 24.99, 23.38 ppm.

HRMS (ESI): Calculated for $C_{17}H_{23}OS$ *m/z* 275.1464 [M-H_2O]$^+$, found *m/z* 275.1480. Error: 5.7 ppm

IR (KBr): ν˜ (cm^{-1}): 3501 (br) (O-H), 2909 (m) (C-H), 2866 (m) (C-H sp^2), 1591 (m) (C=C), 1029 (C-O).

3-((((1R,2S,5S)-2-hydroxy-6,6-dimethylbicyclo[3.1.1]heptan-2yl)methyl)thio) propanoic acid (***3j***)

Synthesis according to the general procedure of thiolysis, using 3-mercaptopropanoic acid (0.5 mmol, 1.6 equiv., 53 mg, 43 μL). In this product, we changed the aqueous solution of NH4Cl for 3 M HCl, until reach pH of 2-3. The product was isolated by column chromatography using Isolera One system, using 30-50% of ethyl acetate-hexane gradient, affording **3j** as a yellowish oil (46 mg, 56% yield).

^{1}H NMR (500 MHz, CD_3OD) δ 2.82 (d, *J* = 7.2 Hz, 1H), 2.81 (d, *J* = 4.2 Hz, 3H), 2.59 (t, *J* = 7.1 Hz, 2H), 2.20 (dtd, *J* = 10.0, 6.1, 2.1 Hz, 1H), 2.12 (dd, *J* = 6.3, 4.9 Hz, 1H), 2.00 – 1.80 (m, 5H), 1.54 (d, *J* = 10.1 Hz, 1H), 1.27 (s, 3H), 0.97 (s, 3H) ppm.

^{13}C NMR (126 MHz, CD_3OD)δ 175.10, 77.59, 52.53, 46.51, 42.03, 38.66, 35.87, 31.23, 29.29, 28.13, 28.05, 25.98, 23.78 ppm.

HRMS (ESI): Calculated for $C_{13}H_{21}O_2S$ *m/z* 241.1257 [M-H_2O]$^+$, found *m/z* 241.1243. Error: -5.6 ppm

IR (KBr): ν˜ (cm^{-1}): 3500 (br) (O-H), 2913 (m) (C-H), 1708 (m) (C=O), 1011 (m) (C-O).

(1R,2S,5S)-2-(((4-fluorphenyl)thio)methyl)-6,6-dimethylbicyclo[3.1.1]heptan-2-ol (***3k***)

Synthesis according to the general procedure of thiolysis, using 4-fluorthiophenol (0.5 mmol, 1.6 equiv., 65.9 mg, 55 μL). The product was isolated by column cromatography using Isolera One system, using 2-10% ethyl acetate-hexane gradient, affording **3k** as a yellowish oil (49.6 mg, 55% yield).

^{1}H NMR (500 MHz, $CDCl_3$) δ 7.44 – 7.40 (m, 2H), 7.01 – 6.96 (m, 2H), 3.24 (d, *J* = 13.4 Hz, 1H), 3.17 (d, *J* = 13.4 Hz, 1H), 2.38 (s, 1H), 2.18 (dtd, *J* = 10.1, 6.2, 2.0 Hz, 1H), 2.04

(dd, J = 6.3, 4.9 Hz, 1H), 1.96 – 1.77 (m, 5H), 1.52 (d, J = 10.1 Hz, 1H), 1.12 (s, 3H), 0.87 (s, 3H) ppm.

^{13}C NMR (126 MHz, CDCl$_3$) δ 162.05 (d, J = 246.9 Hz), 133.18 (d, J = 8.1 Hz), 132.23 (d, J = 3.5 Hz), 116.21 (d, J = 21.9 Hz), 76.33, 51.15, 50.30, 43.43, 38.73, 31.76, 27.39, 27.28, 25.57, 23.81.

HRMS (ESI): Calculated for $C_{16}H_{20}FS$ *m/z* 263.1264 [M-H$_2$O]$^+$, found *m/z* 263.1278. Error: 5.3 ppm.

IR (KBr): ν~ (cm^{-1}): 3451 (br) (O-H), 2911 (m) (C-H), 2867 (C-H sp^2), 1589 (m) (C=C), 1023 (m) (C-O).

$[\alpha]_D^{21}$ = –39.90º (c=0.8 in acetone)

(1R,2S,5S)-6,6-dimethyl-2-((pyridin-2-ylthio)methyl)bicyclo[3.1.1]heptan-2-ol (***3l***)

Synthesis according to the general procedure of thiolysis, using 2-mercaptopyridine (0.5 mmol, 1.6 equiv., 56 mg). The product was isolated by column chromatography using Isolera One system, using 5-15% of ethyl acetate-hexane, affording **3l** as a yellowish oil (45 mg, 53% yield).

^{1}H NMR (500 MHz, CDCl$_3$)δ 8.35 – 8.29 (m, 1H), 7.47 (td, J = 7.7, 1.8 Hz, 1H), 7.27 (d, J = 8.1 Hz, 1H), 6.99 (ddd, J = 7.5, 5.0, 1.1 Hz, 1H), 5.86 (s, 1H), 3.46 (d, J = 14.9 Hz, 1H), 3.35 (d, J = 14.8 Hz, 1H), 2.26 – 2.20 (m, 1H), 2.17 (t, J = 5.6 Hz, 1H), 2.05 – 1.77 (m, 5H), 1.62 (d, J = 10.0 Hz, 1H), 1.24 (s, 3H), 0.98 (s, 3H) ppm.

^{13}C NMR (101 MHz, CDCl$_3$)δ 159.50, 148.64, 136.37, 122.74, 119.83, 77.38, 77.06, 76.74, 75.68, 52.22, 44.14, 40.67, 38.10, 31.48, 27.57, 27.16, 25.02, 23.43 ppm.

HRMS (ESI): Calculated for $C_{15}H_{20}NS$ *m/z* 246.1311 [M-H$_2$O]$^+$, found *m/z* 246.1311. Error: 0 ppm

IR (KBr): ν~ (cm^{-1}): 3510 (br) (O-H), 2916 (m) (C-H), 2866 (C-H sp^2), 1709 (m) (C=C), 1579 (m) (C=N), 1025 (m) (C-O).

(1R,2S,5S)-6,6-dimethyl-2-(((4-(methylthio)phenyl)thio)methyl)bicyclo[3.1.1]heptan-2-ol (***3m***)

Synthesis according to the general procedure of thiolysis, using 4-(methylsulfanyl)thiophenol (0.5 mmol, 1.6 equiv., 78 mg, 65 μL). The product was isolate using Isolera One system, using 2-10% of ethyl acetate-hexane gradient, affording **3m** as yellowish oil (50.6 mg, 51% yield).

^{1}H NMR (500 MHz, $CDCl_3$) δ 7.37 – 7.33 (m, 2H), 7.19 – 7.15 (m, 2H), 3.25 (d, *J* = 13.4 Hz, 1H), 3.17 (d, *J* = 13.4 Hz, 1H), 2.46 (s, 3H), 2.40 (s, 1H), 2.17 (dtd, *J* = 10.2, 6.1, 2.0 Hz, 1H), 2.04 (dd, J = 6.2, 4.9 Hz, 1H), 1.95 – 1.78 (m, 5H), 1,52 (d, *J* = 10.2 Hz, 1H), 1.13 (s, 3H), 0.89 (s, 3H) ppm.

^{13}C NMR(126 MHz, $CDCl_3$) δ 138.87, 133.55, 131.40, 127.39, 74.93, 51.20, 49.15, 40.90, 38.81, 31.17, 27.40, 27.30, 24.99, 23.41, 16.16 ppm.

HRMS (ESI): Calculated for $C_{17}H_{23}S_2$*m/z* 291.1236 $[M\text{-}_{H2}O]^+$, found *m/z* 291,1231. Error:1.7 ppm.

IR (KBr): ν˜ (cm^{-1}): 3488 (br) (O-H), 2916 (m) (C-H), 2866 (m) (C-H sp^2) 1707 (m) (C=C), 1577 (m) (C=C), 1072 (m) (C-O).

(1R,2S,5S)-2-((ethylthio)methyl)-6,6-dimethylbicyclo[3.1.1]heptan-2-ol (***3n***)

Synthesis according to the general procedure of thiolysis, using ethanethiol (0.5 mmol, 1.6 equiv., 31.1 mg, 37 µL). This reaction was conducted at room temperature for 48 h. The product was isolated in Isolera One using 2-10% ethyl acetate-hexane, affording **3n** as yellowish oil (33.7 mg, 49% yield).

^{1}H NMR (500 MHz, $CDCl_3$) δ 2.85 (d, *J* = 13.3 Hz, 1H), 2.75 (d, *J* = 13.3 Hz, 1H), 2.56 (qd, J = 7.4, 1.6 Hz, 2H), 2.18 (dtd, *J* = 10.0, 6.2, 1.9 Hz, 1H), 2.04 – 1.99 (m, 1H), 1.96 – 1.75 (m, 5H), 1.53 (d, *J* = 10.1 Hz, 1H), 1.24 (t, *J* = 7.4 Hz, 3H), 1.23 (s, 3H), 0.94 (s, 3H) ppm.

^{13}C NMR (126 MHz, $CDCl_3$) δ 76.55, 51.29, 47.80, 41.77, 37.88, 30.50, 28.35, 27.62, 27.41, 25.03, 21.94, 13.99 ppm.

HRMS (ESI): Calculated for $C_{12}H_{21}S$ *m/z* 197.1358 [M-H_2O]+, found *m/z* 197.1368. Error: 4.8 ppm

IR (KBr): ν˜ (cm^{-1}): 3019 (br) (3019), 1150 (m) (C-O).

(1R,2S,5S)-2(((1H-benzo[d]imizadol-2-yl)thiomethyl-6-6-dimethylbicyclo[3.1.1] heptan-2-ol (***3o***)

Synthesis according to the general procedure of thiolysis, using 1*H*-2-mercapto benzimidazole (0.5 mmol, 1.6 equiv., 75 mg). In this product, the pH was adjusted to 13 by the addition of NaOH. The product was isolated by column chromatography using Isolera One system, using 5-100% of ethyl acetate-hexane, affording **3o** as a white solid (23.4 mg, 24% yield).

^{1}H NMR (500 MHz, CD_3OD) δ 7.43 (dt, *J* = 7.2, 3.5 Hz, 2H), 7.19 – 7.14 (m, 2H), 3.60 (d, *J* = 13.4 Hz, 1H), 3.51 (d, *J* = 13.4 Hz, 1H), 2.27 – 2.19 (m, 1H), 2.17 (dd, *J* = 6.3, 4.8 Hz,

1H), 2.12 – 2.04 (m, 1H), 2.00 – 1.80 (m, 4H), 1.56 (d, *J* = 10.1 Hz, 1H), 1.18 (s, 3H), 0.98 (s, 3H) ppm,

^{13}C NMR(126 MHz, CD_3OD)δ 151.91, 122.59, 121.83, 108.35, 75.64, 51.74, 45.85, 40.01, 37.08, 30.30, 27.56, 26.12, 24.51, 21.34 ppm.

HRMS (ESI): Calculated for $C_{17}H_{23}N_2OS$ *m/z* 303.1526 $[M+H]^+$, found *m/z* 303.1548. Error: 7.3 ppm

IR (KBr): ν˜ (cm^{-1}): 3150 (br) (O-H), 2924 (m) (C-H), 2867 (m) (C-H sp^2), 1739 (w) (C=C), 1672 (w) (C=C), 1043 (m) (C-O).

(1R,2S,5S)-6,6-dimethyl-2-((pyrimidin-2-ylthio)methyl)bicyclo[3.1.1]heptan-2-ol (***3p***)

Synthesis according to the general procedure of thiolysis, using 2-mercapto pyrimidine (0.5 mmol, 1.6 equiv., 56.1 mg). In this product, the pH was adjusted to 9-10 by the addition of 1 M NaOH. The product was isolated by column chromatography using Isolera One system, using 20-50% of ethyl acetate-hexane, affording **3p** as yellowish solid (16.5 mg, 19% yield).

^{1}H NMR(500 MHz, $CDCl_3$) δ 8.35 – 8.29 (m, 1H), 7.47 (td, *J* = 7.7, 1.8 Hz, 1H), 7.27 (d, *J* = 8.1 Hz, 1H), 6.99 (ddd, *J* = 7.5, 5.0, 1.1 Hz, 1H), 5.86 (s, 1H), 3.46 (d, *J* = 14.9 Hz, 1H), 3.35 (d, *J* = 14.8 Hz, 1H), 2.26 – 2.20 (m, 1H), 2.17 (t, *J* = 5.6 Hz, 1H), 2.05 – 1.77 (m, 5H), 1.62 (d, *J* = 10.0 Hz, 1H), 1.24 (s, 3H), 0.98 (s, 3H) ppm.

^{13}C NMR (126 MHz, $CDCl_3$) δ 173.77, 157.12, 117.60, 75.84, 52.02, 42.86, 40.72, 38.17, 29.80, 28.37, 27.12, 26.36, 23.35 ppm.

HRMS (ESI): Calculated for $C_{14}H_{20}N_2OSLi$ *m/z* 271.1451 $[M+Li]^+$, found *m/z* 271.1451. Error: 0 ppm.

IR (KBr): ν˜ (cm^{-1}): 3430 (br) (O-H), 2933 (m) (C-H), 2840 (C-H sp^2), 1709 (m) (C=C), 1545 (m) (C=N), 1028 (m) (C-O).

5. ^{1}H and ^{13}C NMR of β-pinene epoxide (2) and β-hydroxysulfides derivatives 3a-3p

^{1}H NMR (500 MHz, $CDCl_3$) of **2**

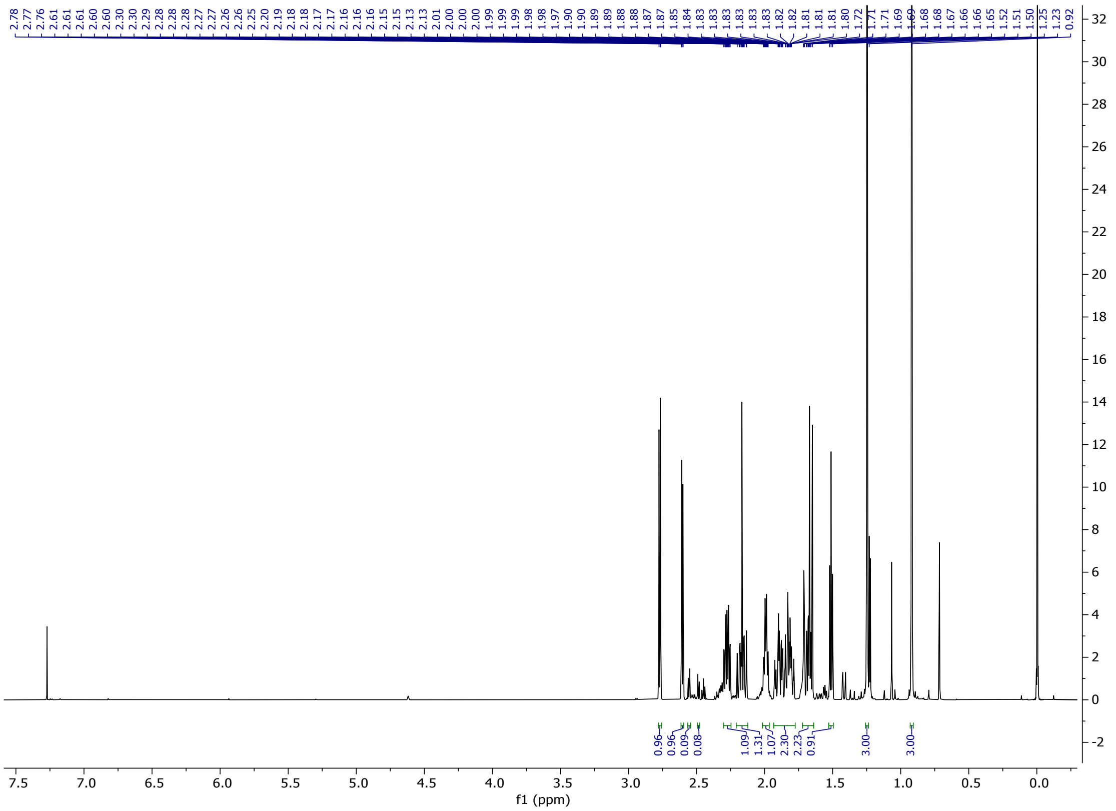

^{13}C NMR (126 MHz, $CDCl_3$) of **2**

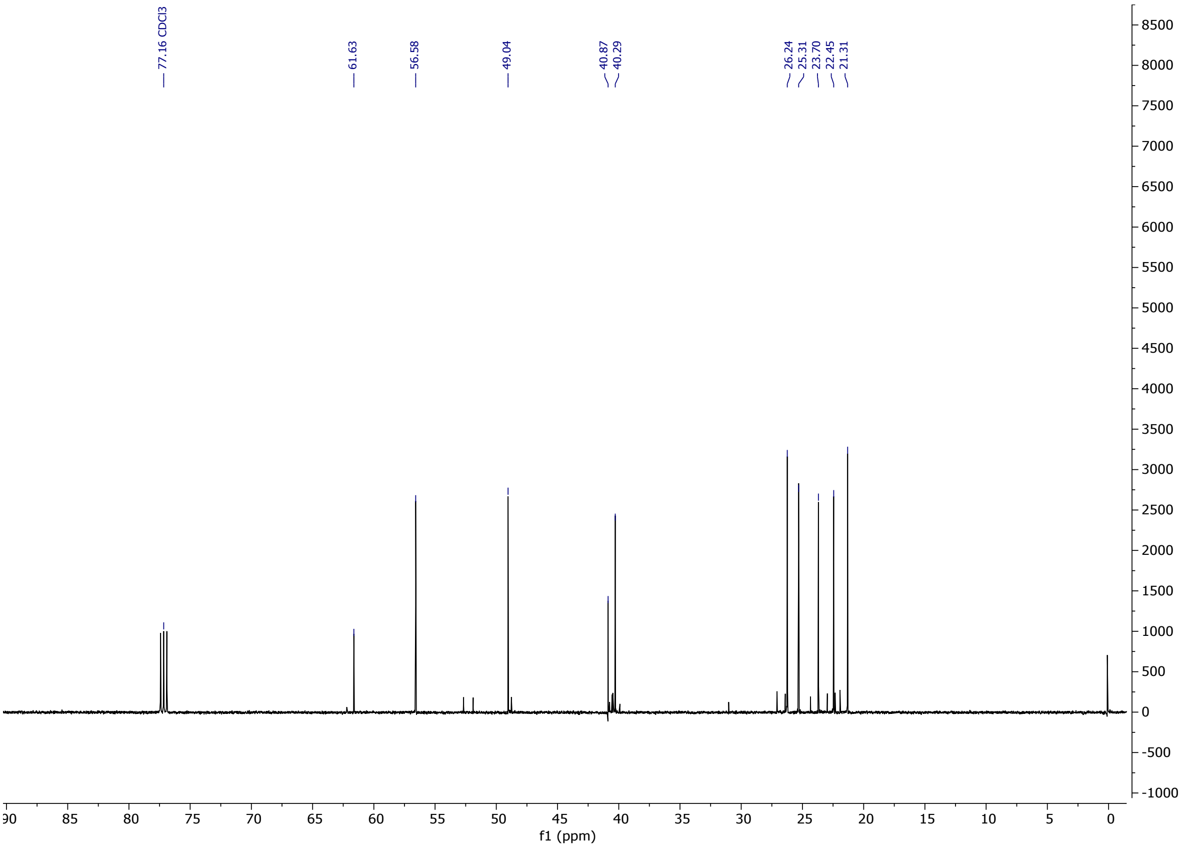

2D NOESY (500 MHz, $CDCl_3$)of **2**

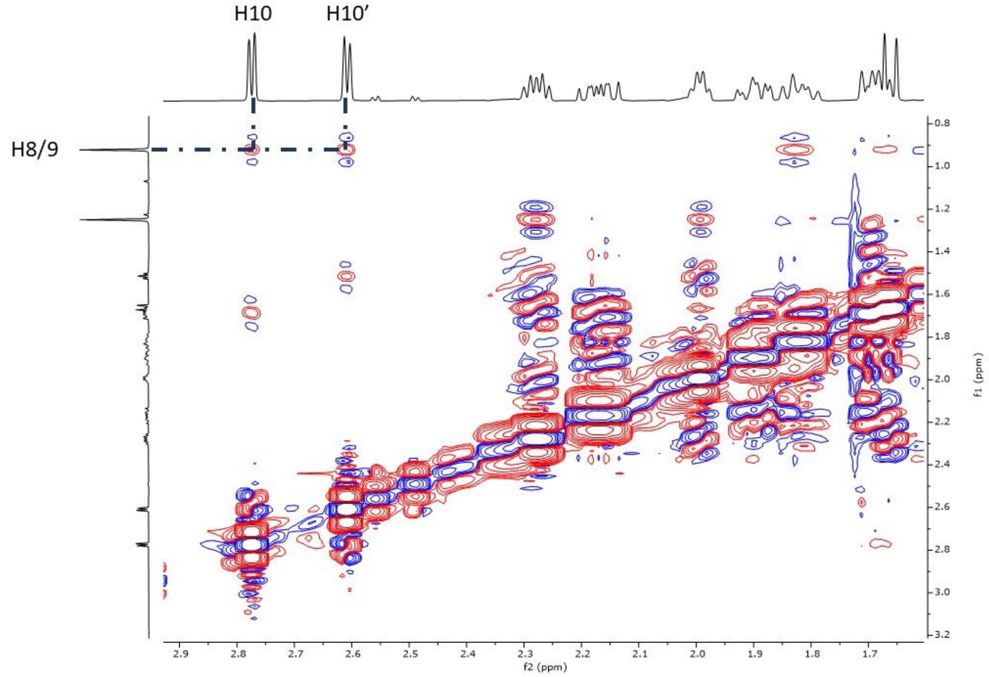

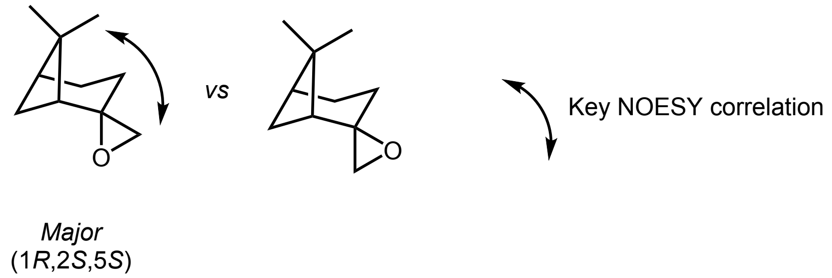

Figure S1. KeyNOESY correlation of **2** for major diastereoisomer identification.

^{1}H NMR (500 MHz. $CDCl_3$) of **3a**

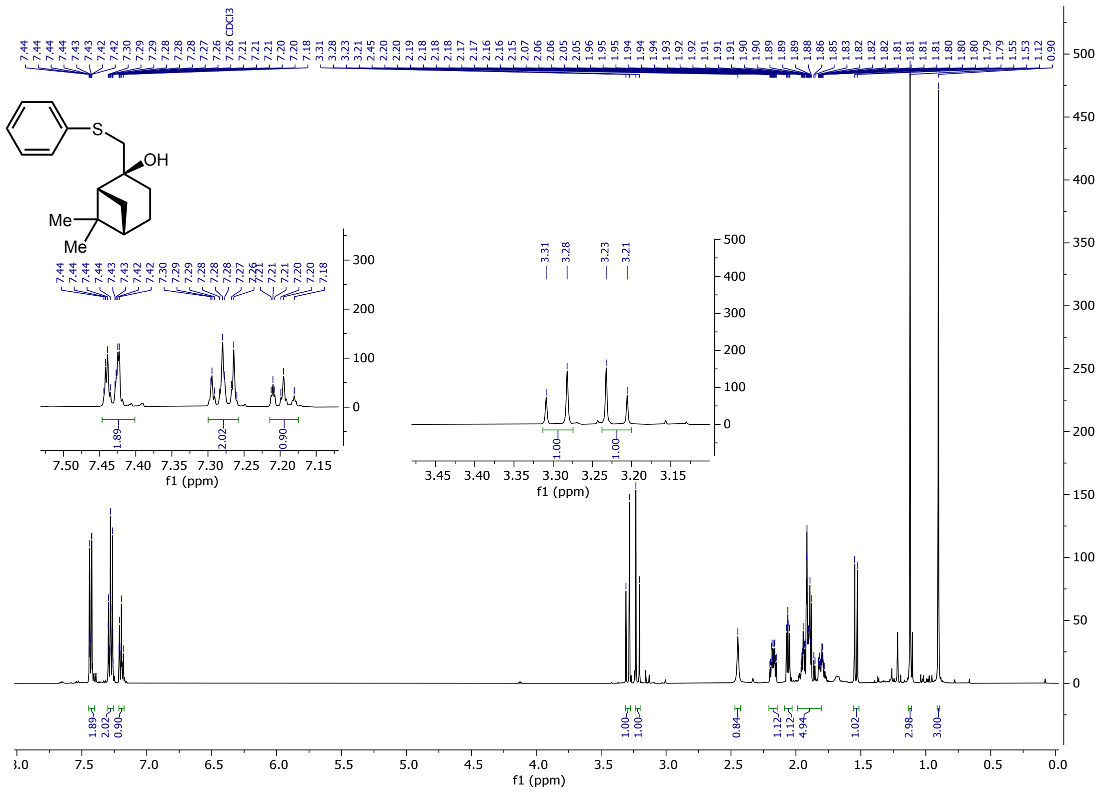

^{13}C NMR (126 MHz, $CDCl_3$) of **3a**

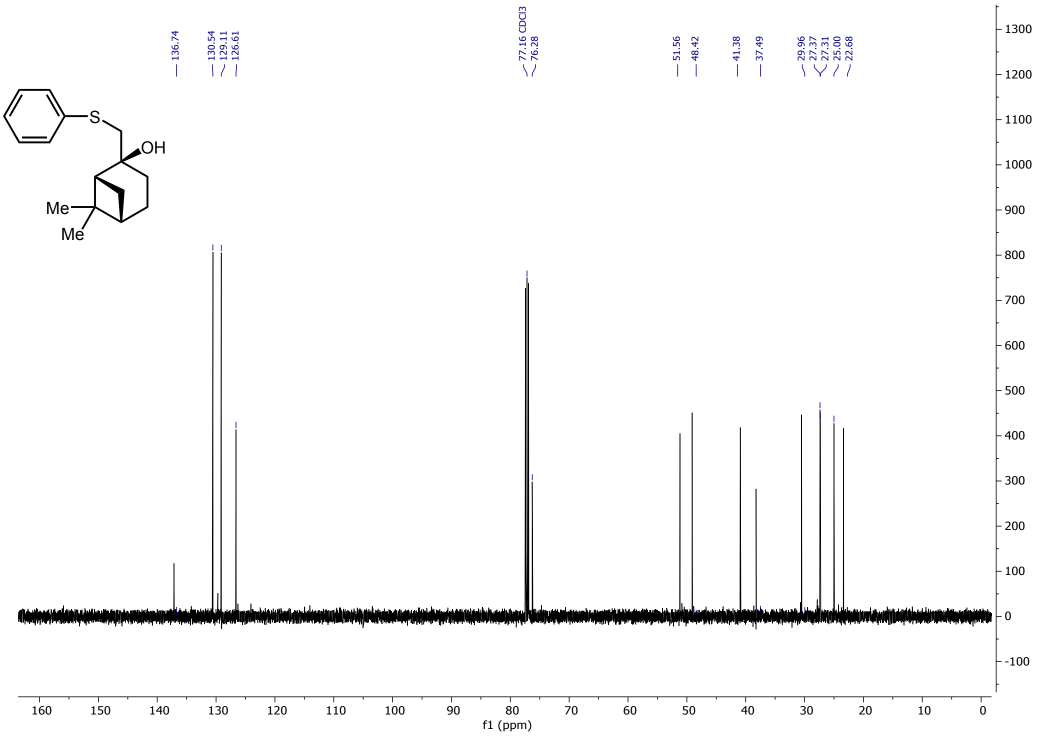

HSQC (500 MHz, $CDCl_3$) of **3a**

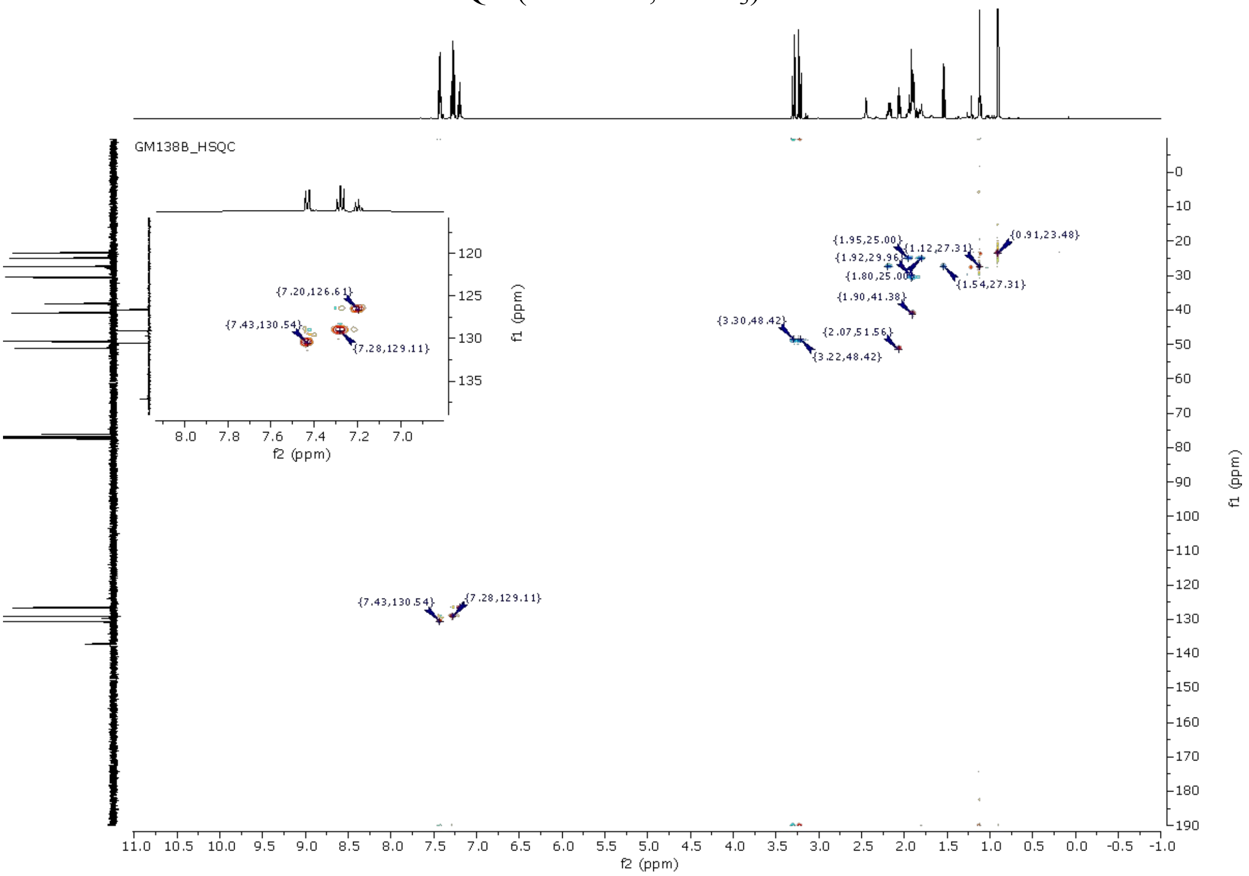

HMBC (500 MHz, $CDCl_3$) of **3a**

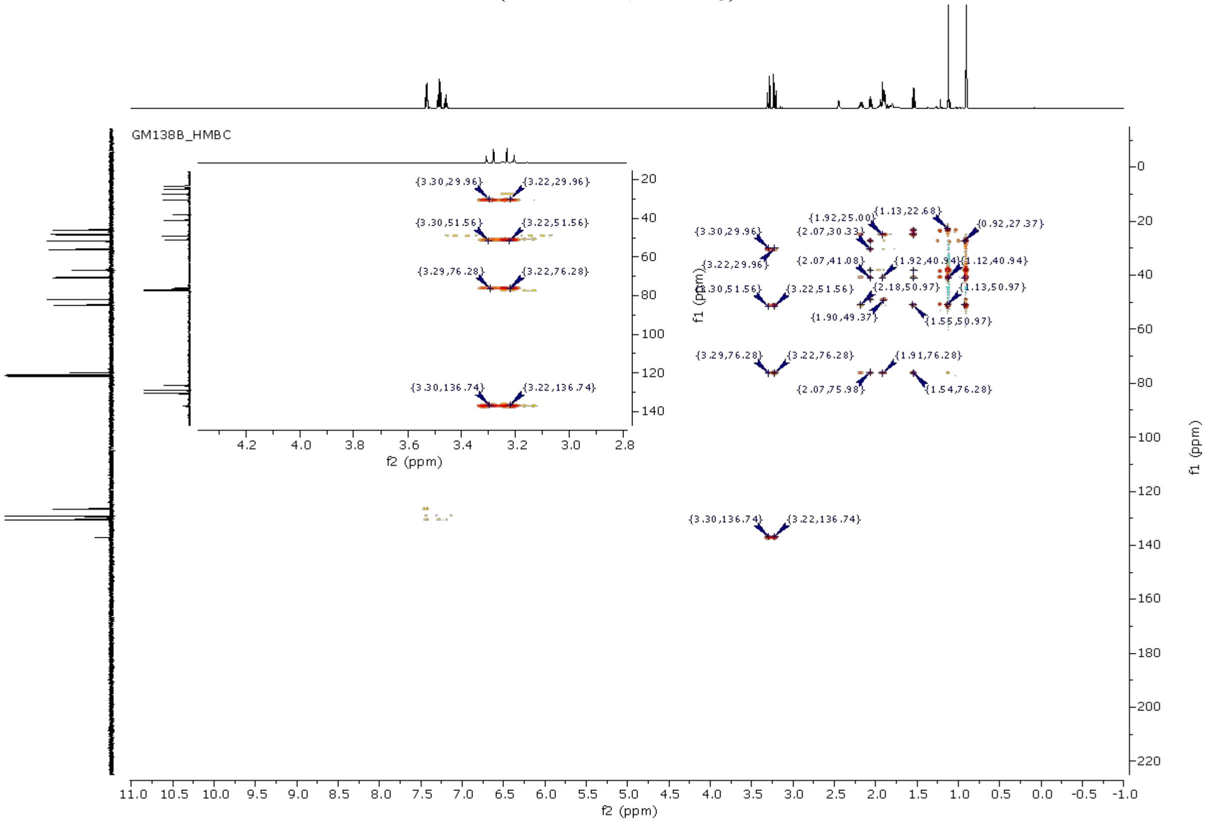

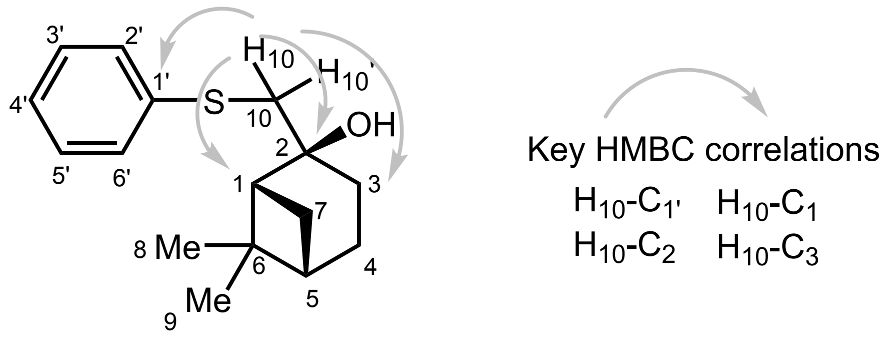

Figure S2. Key HMBC correlations for product elucidation of **3a**

^{1}H NMR (500 MHz, $CDCl_3$) of **3b**

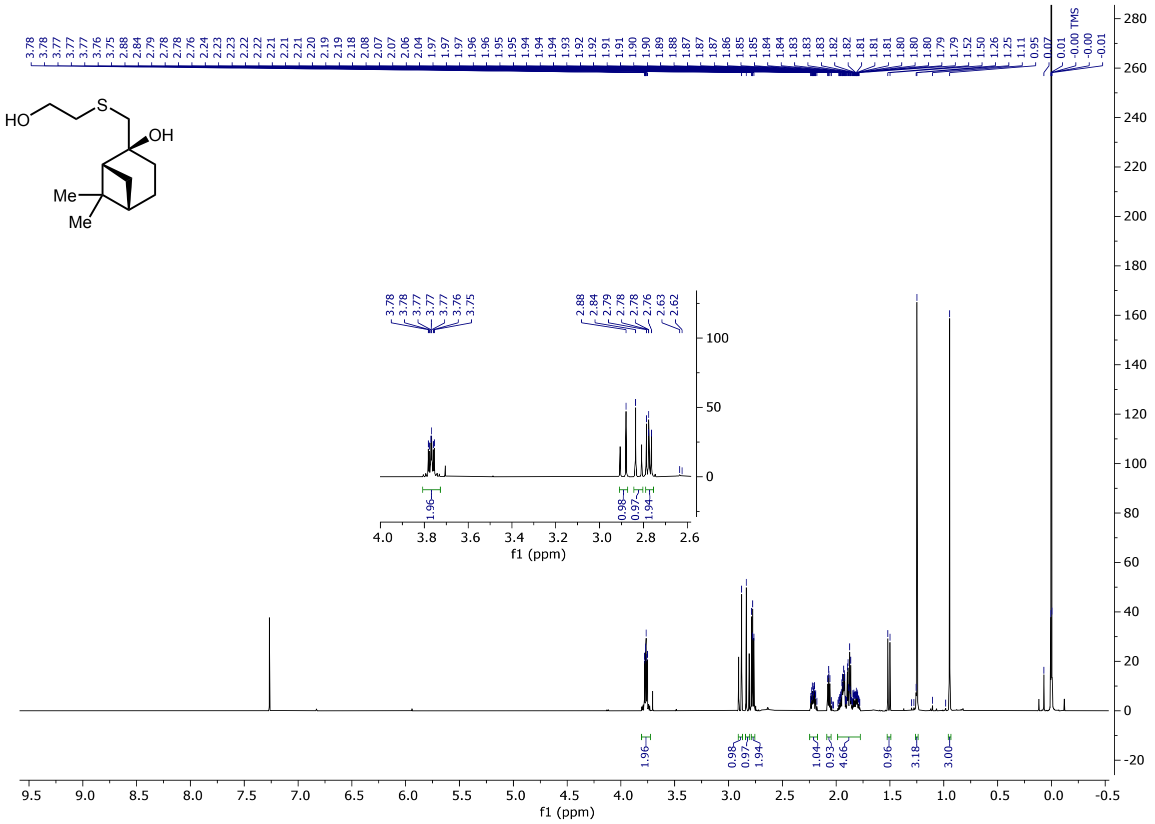

^{13}C NMR (126 MHz, $CDCl_3$) of **3b**

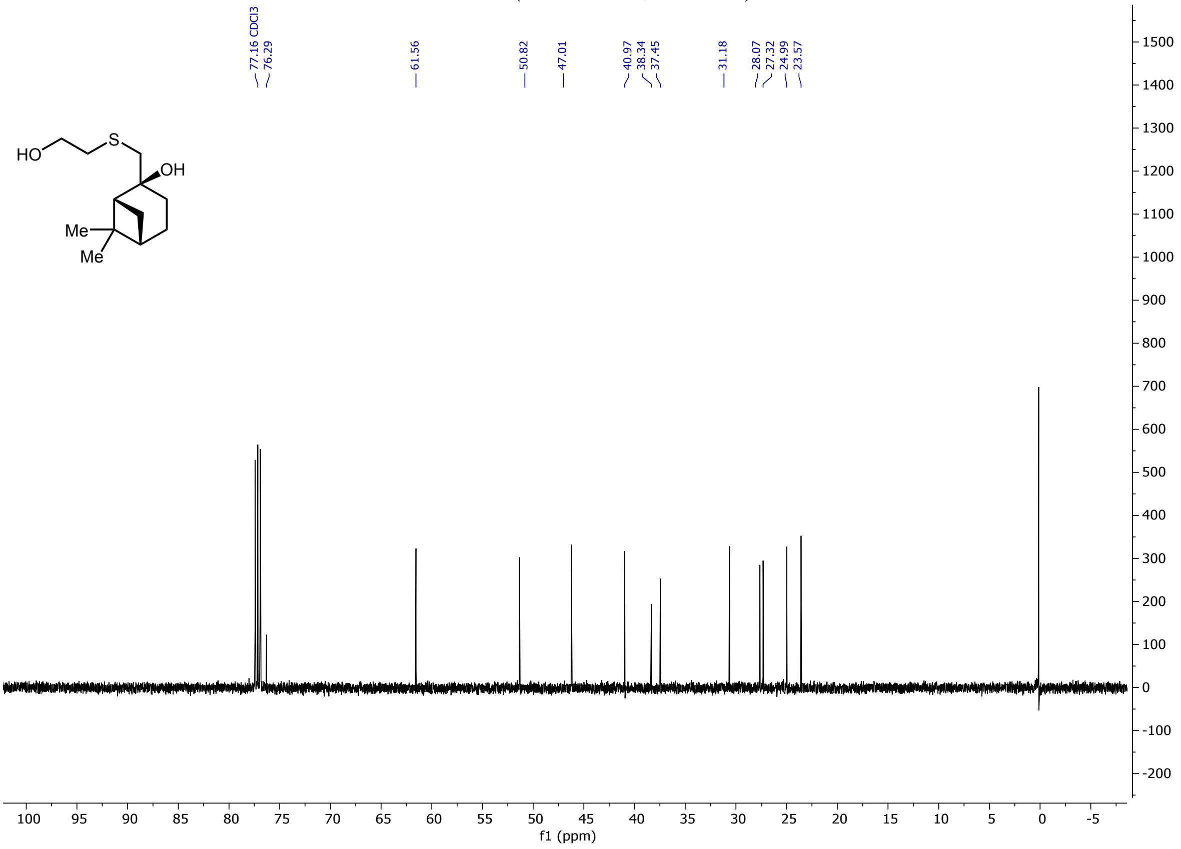

^{1}H NMR (500 MHz, $CDCl_3$) of **3c**

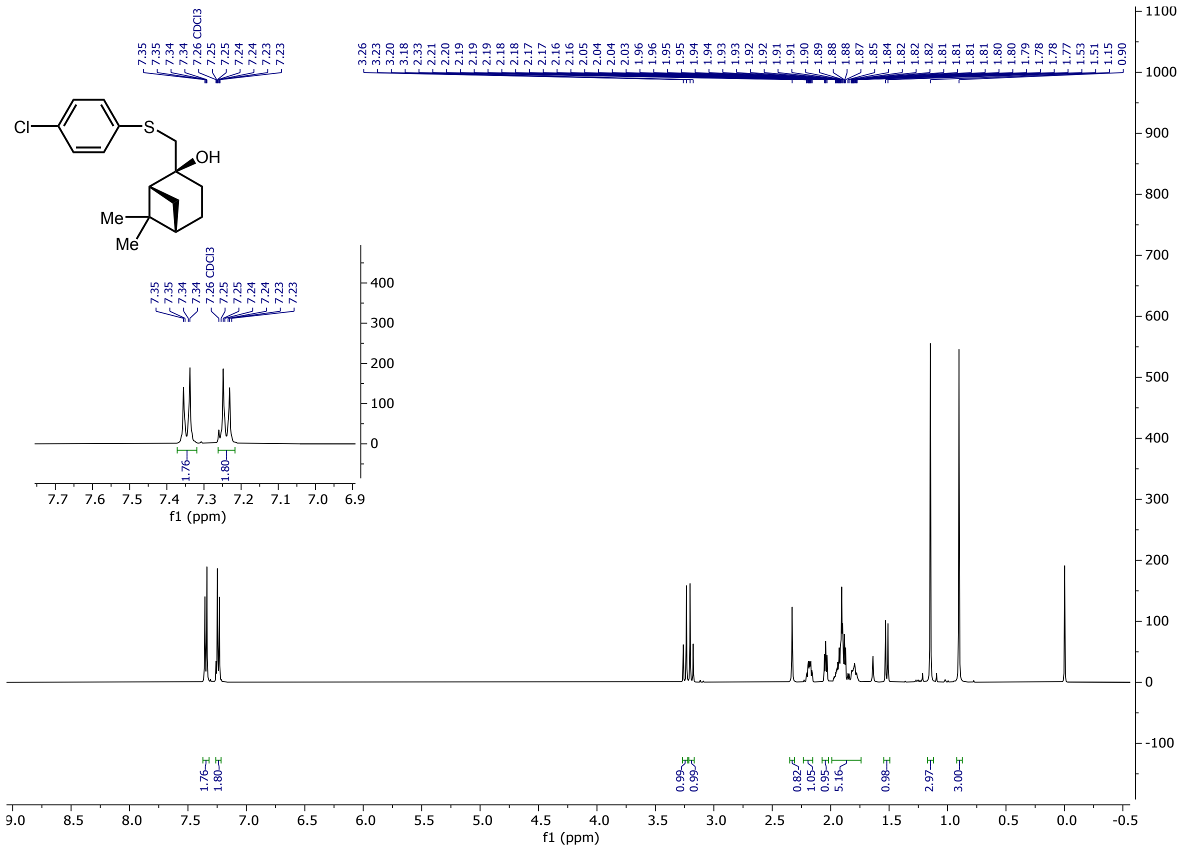

^{13}C NMR (126 MHz, $CDCl_3$) of **3c**

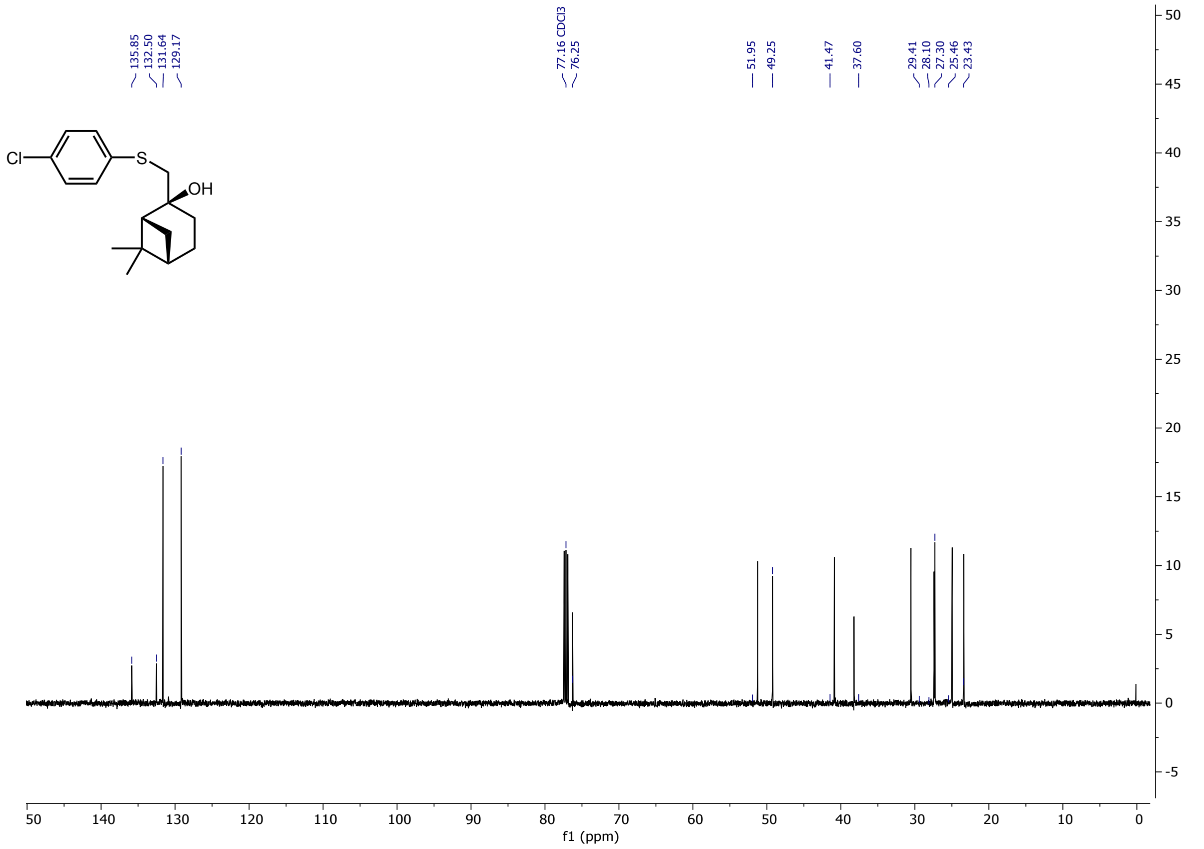

^{1}H NMR (500 MHZ, $CDCl_3$) of**3d**

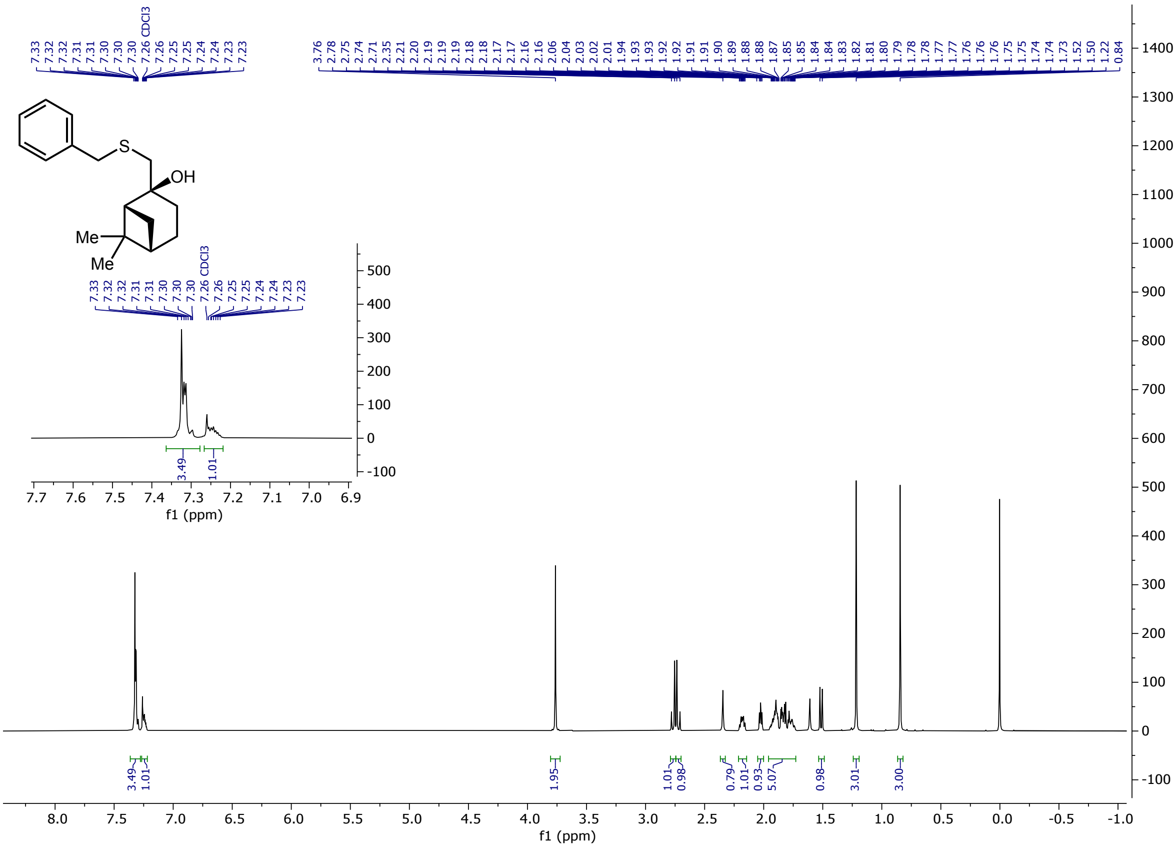

^{13}C NMR (126 MHz, CDCl$_3$) of **3d**

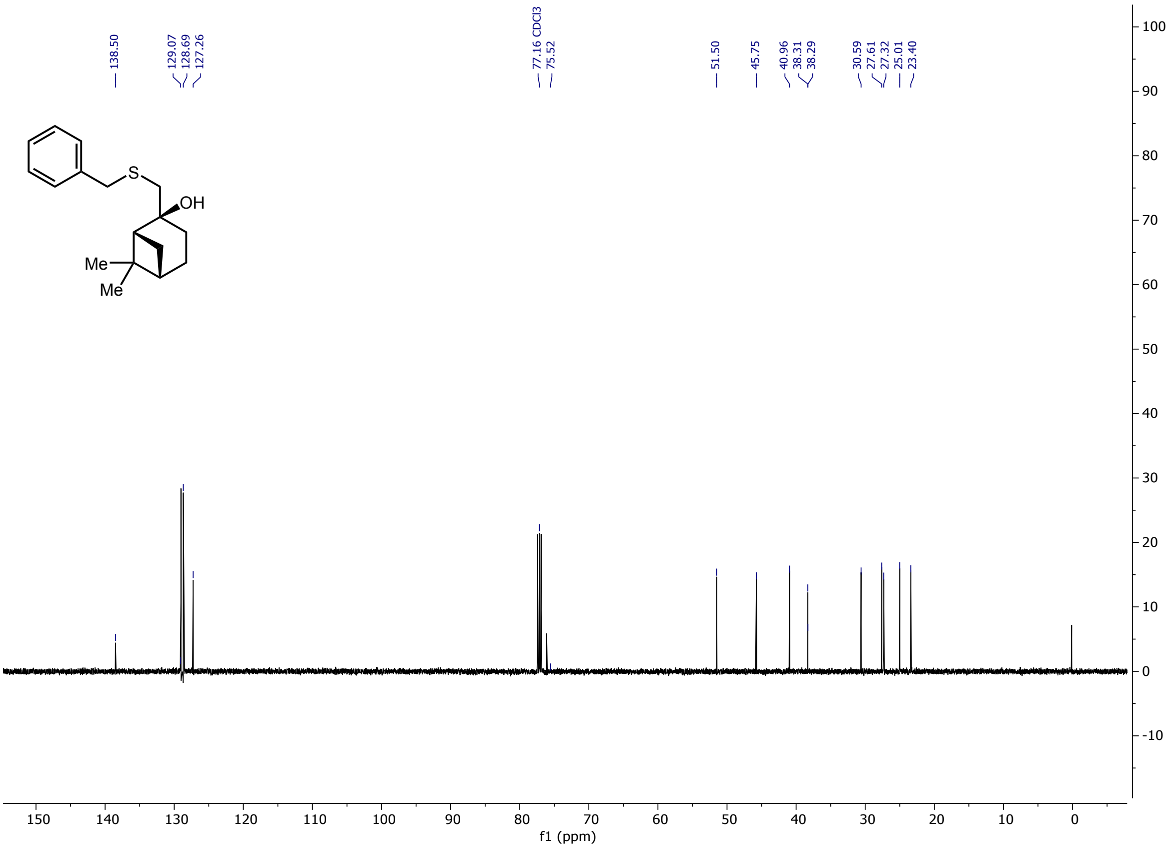

HSQC (500 MHz, CDCl$_3$) of **3d**

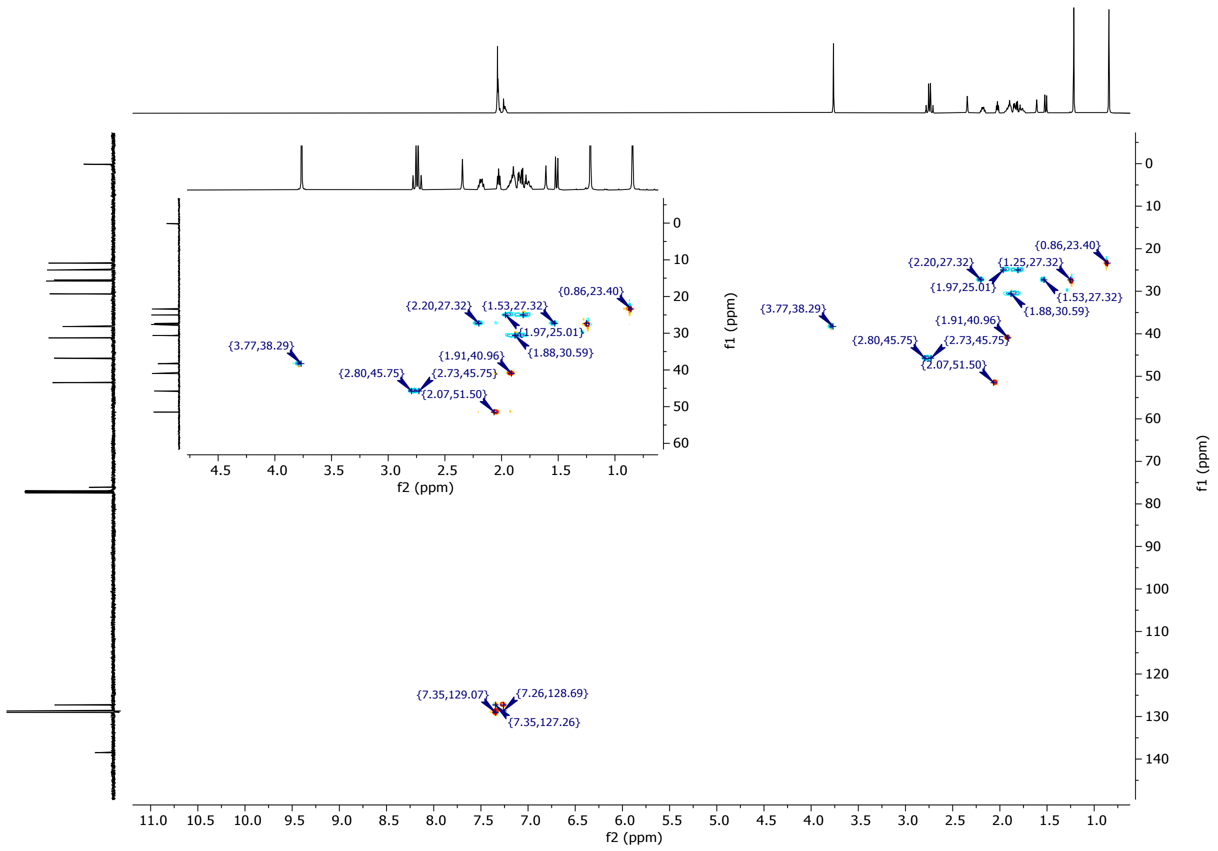

HMBC (500 MHz, $CDCl_3$) of **3d**

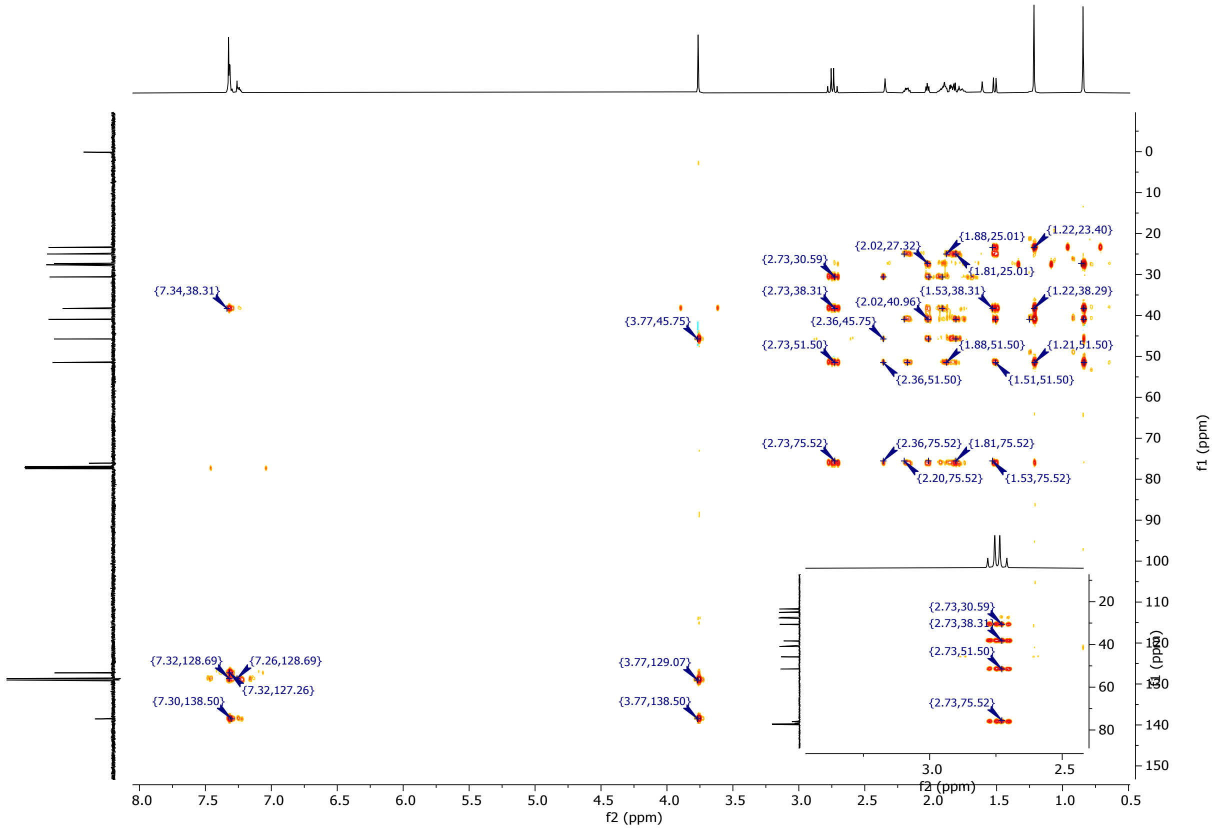

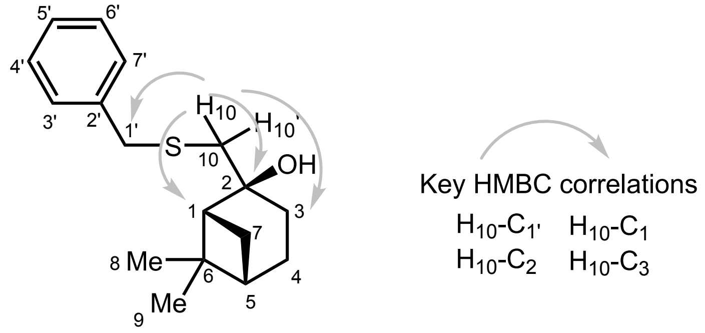

Figure S3. Key HMBC correlations for product elucidation of **3d**

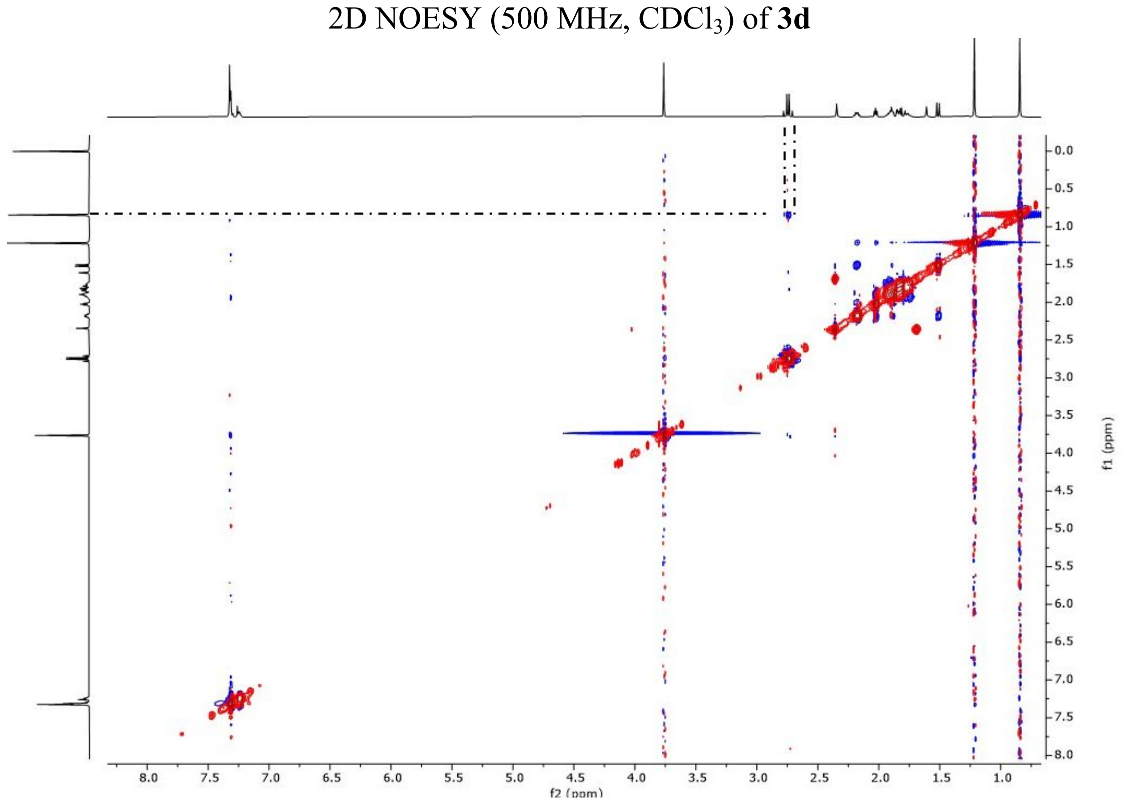

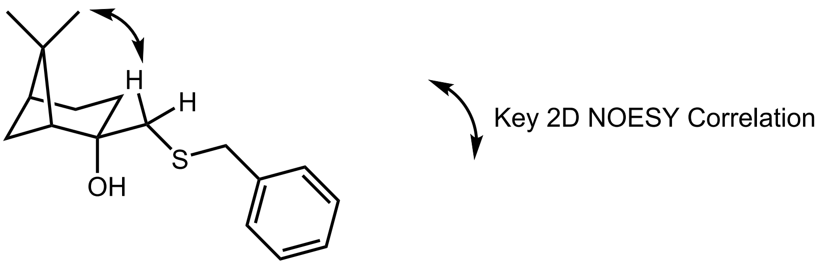

^{1}H NMR (500 MHz, $CDCl_3$) of **3e**

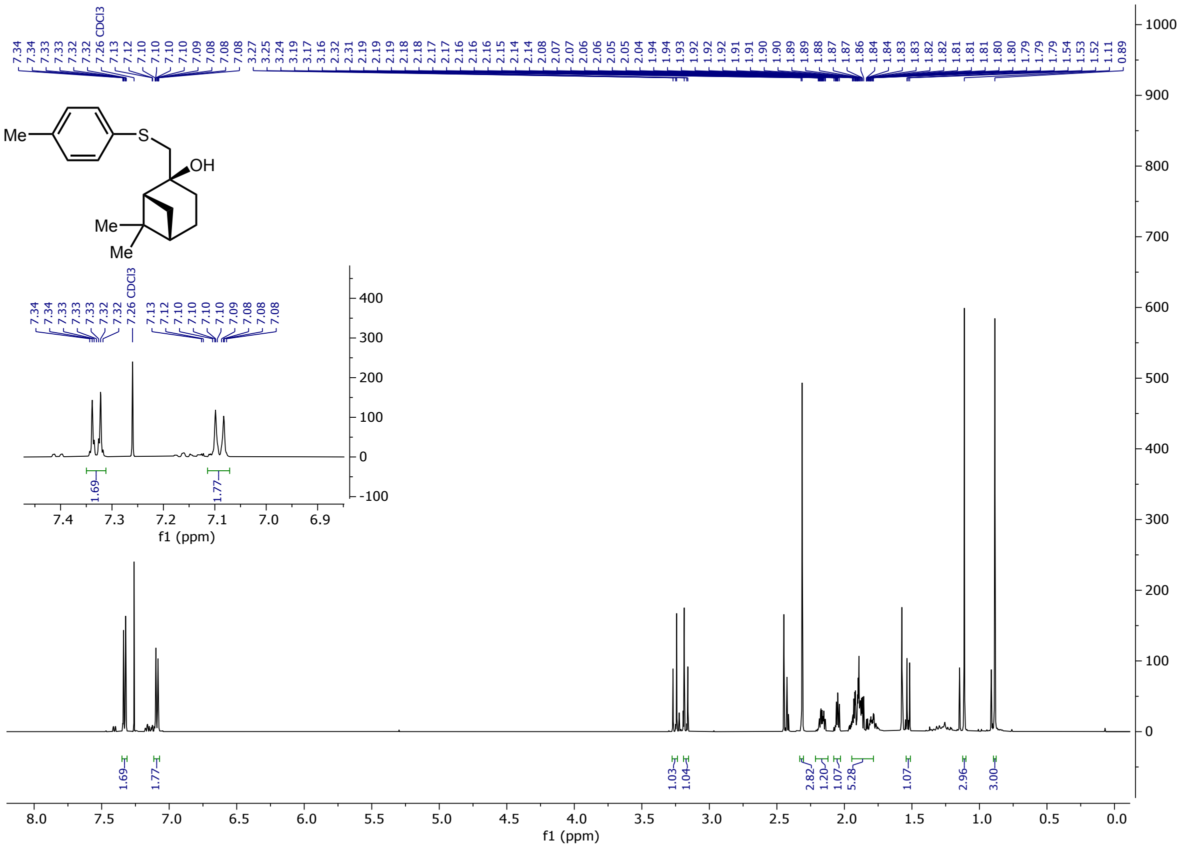

^{13}C NMR (126 MHz, $CDCl_3$) of **3e**

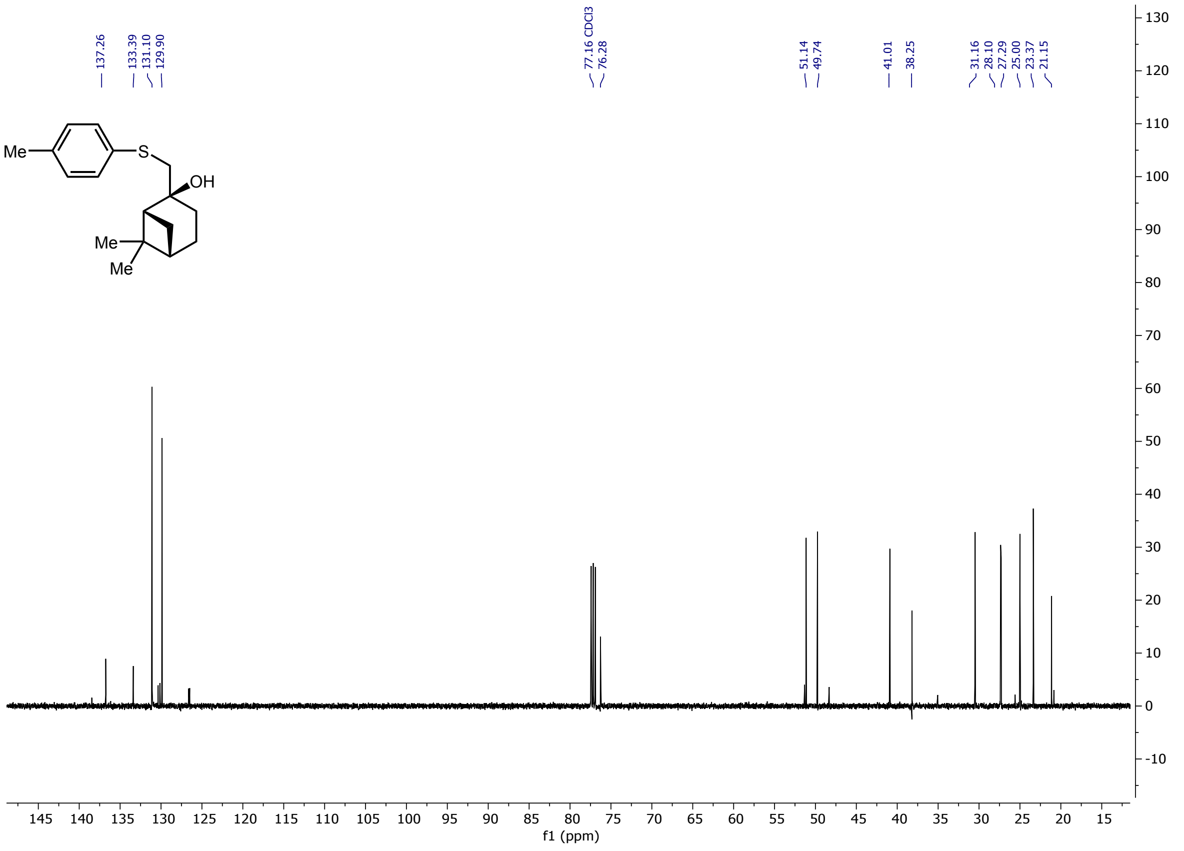

^{1}H NMR (500 MHz, $CDCl_3$) of **3f**

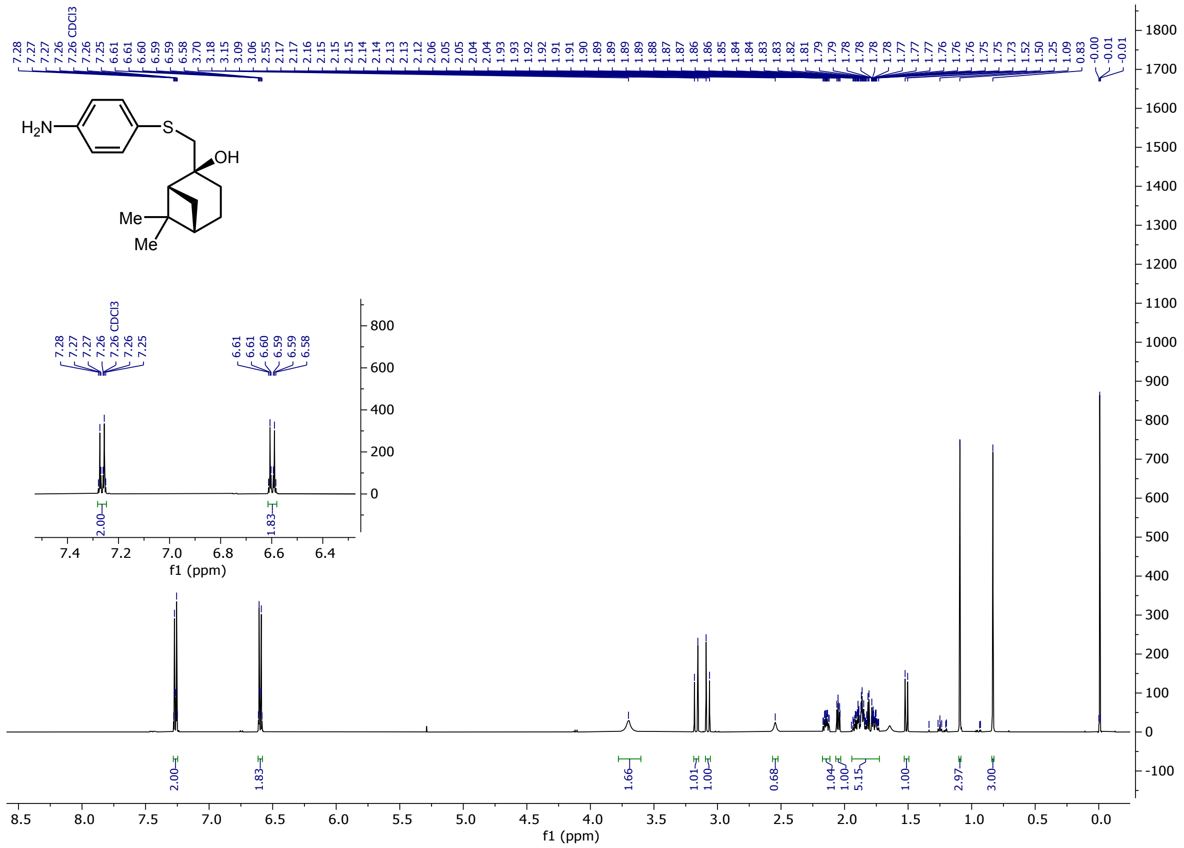

^{13}C NMR (126 MHz, $CDCl_3$) of **3f**

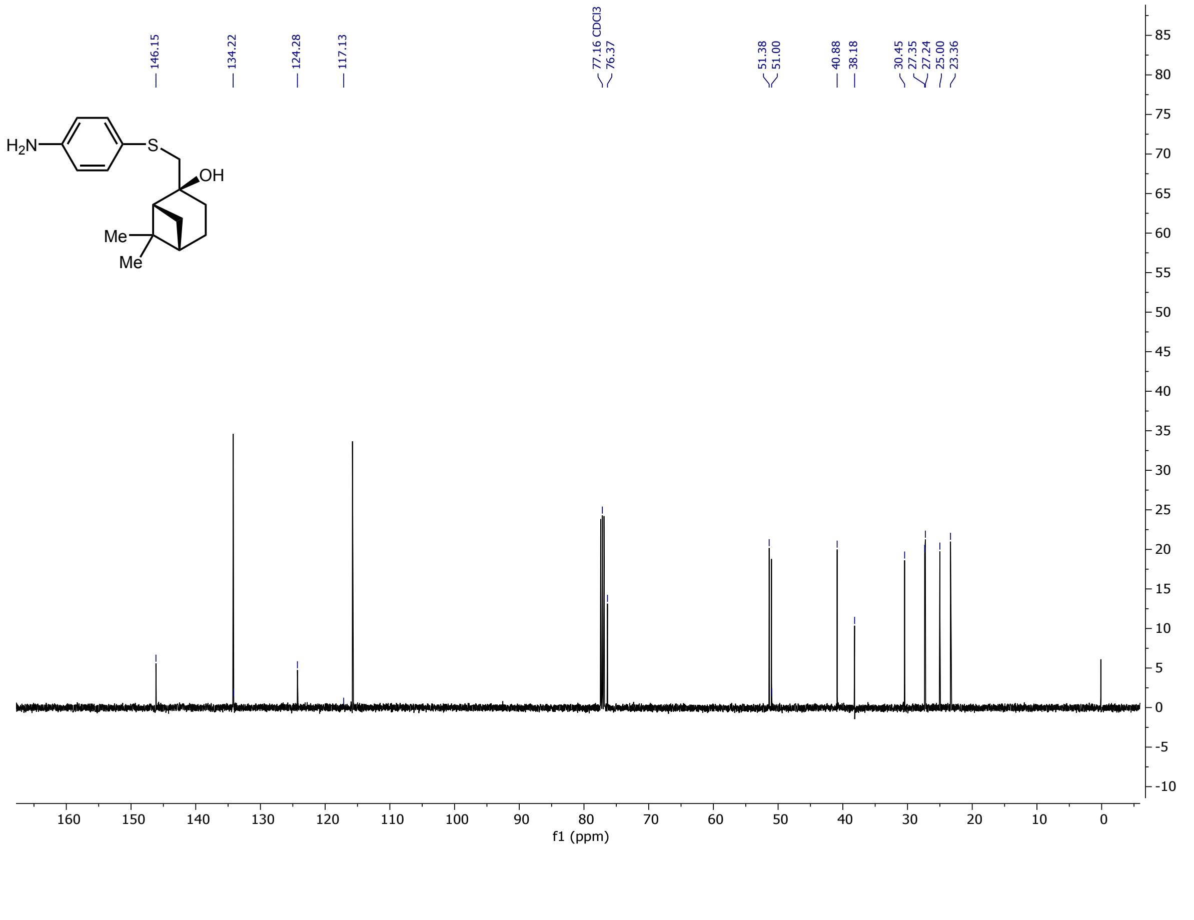

^{1}H NMR (500 MHz, CDCl$_3$) of **3g**

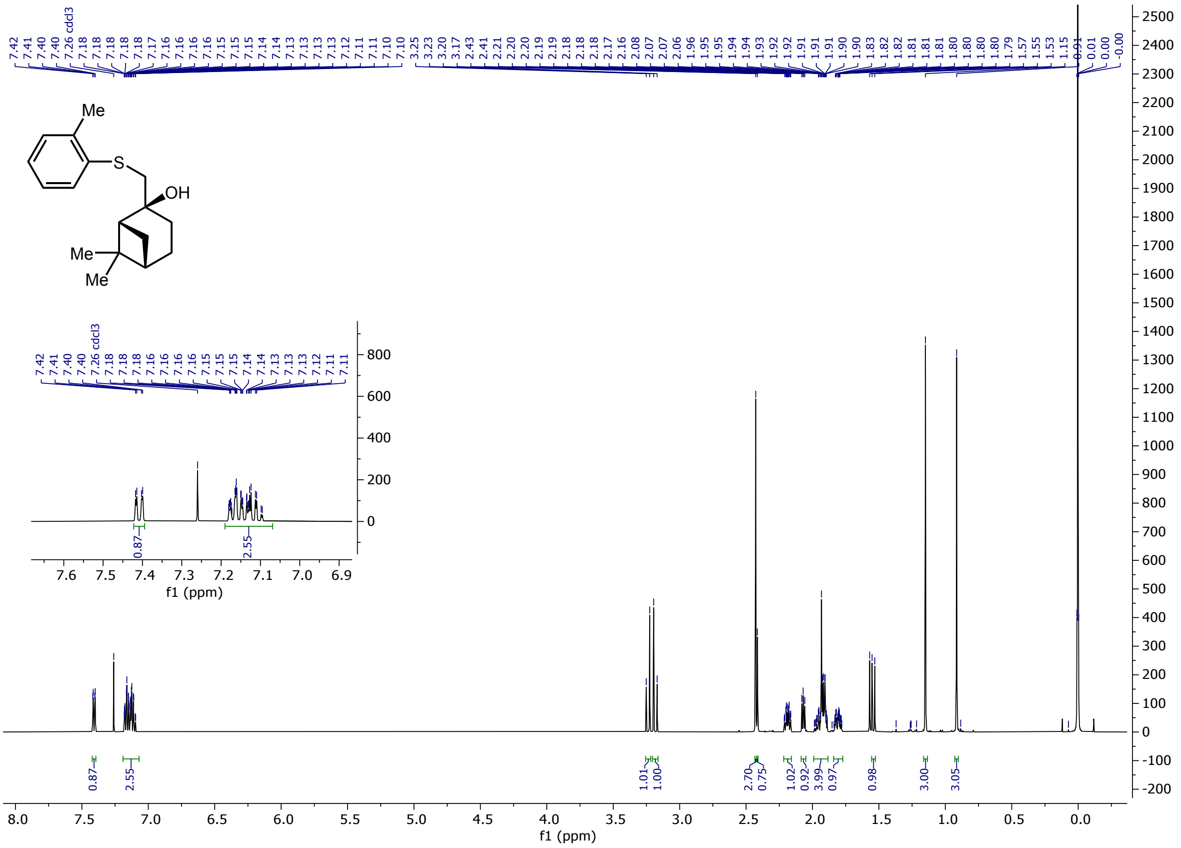

^{13}C NMR (126 MHz, CDCl$_3$) of **3g**

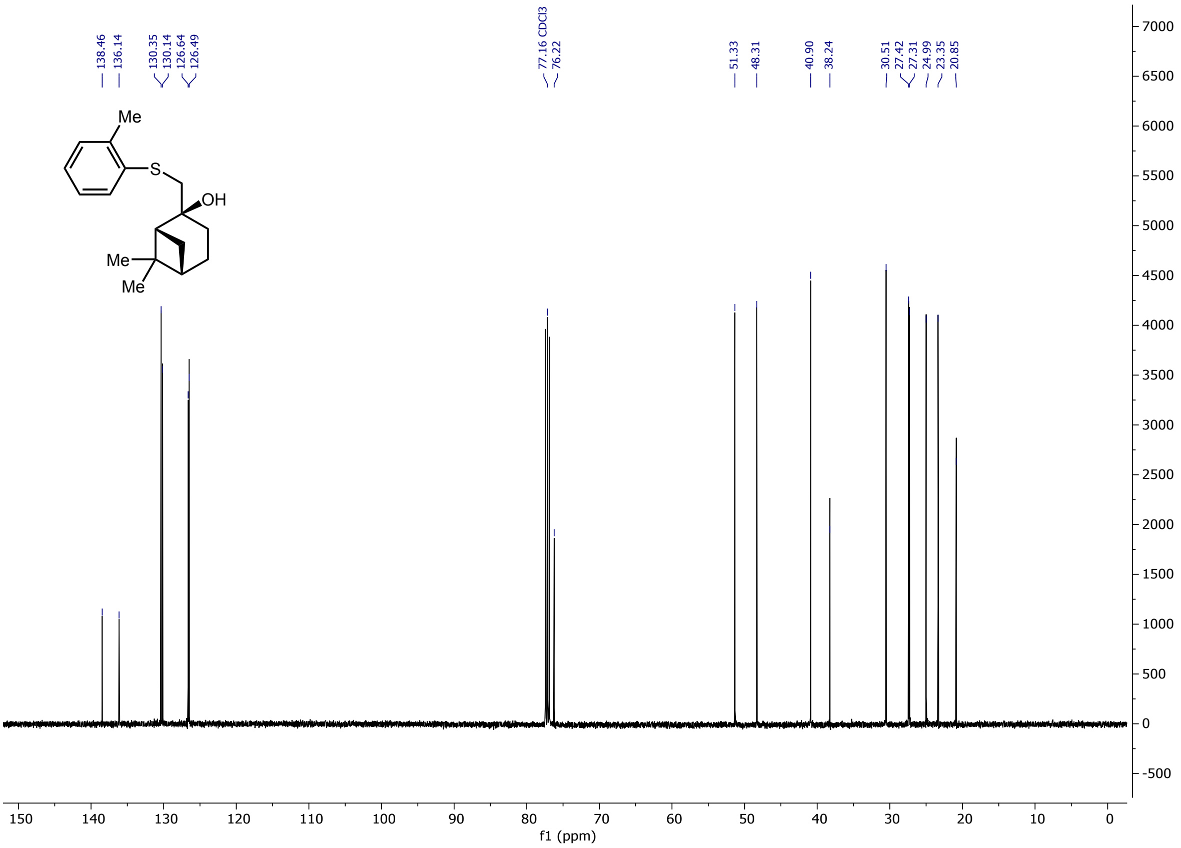

^{1}H NMR (500 MHz, $CDCl_3$) of **3h**

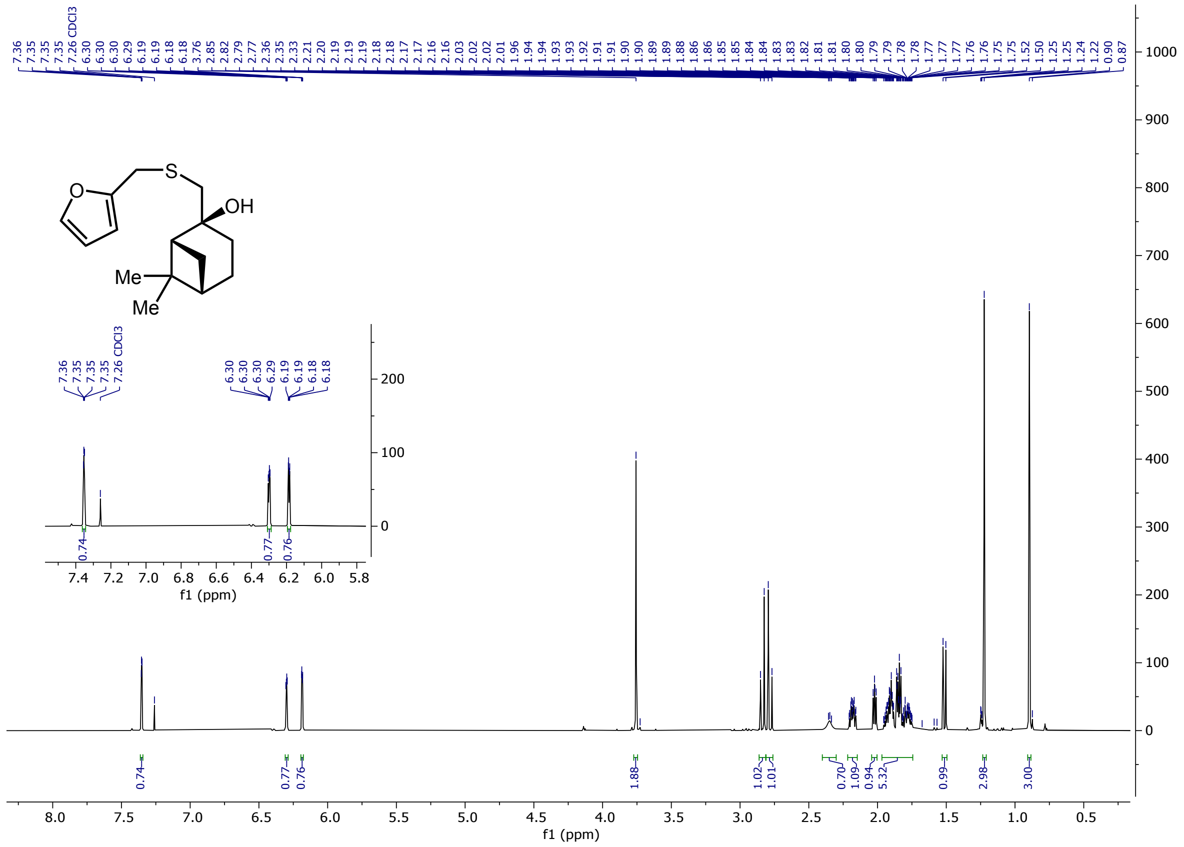

^{13}C NMR (126 MHz, $CDCl_3$) of **3h**

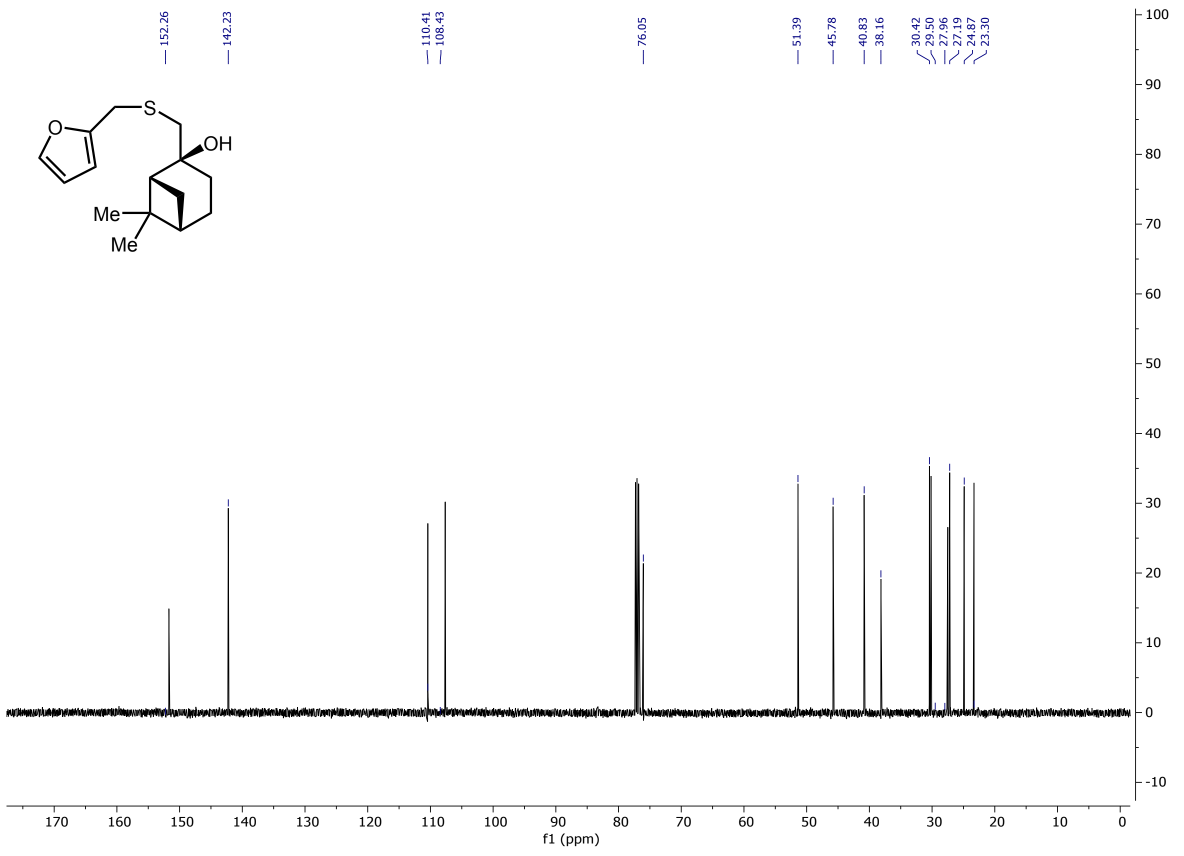

^{1}H NMR (500 MHz, $CDCl_3$) of **3i**

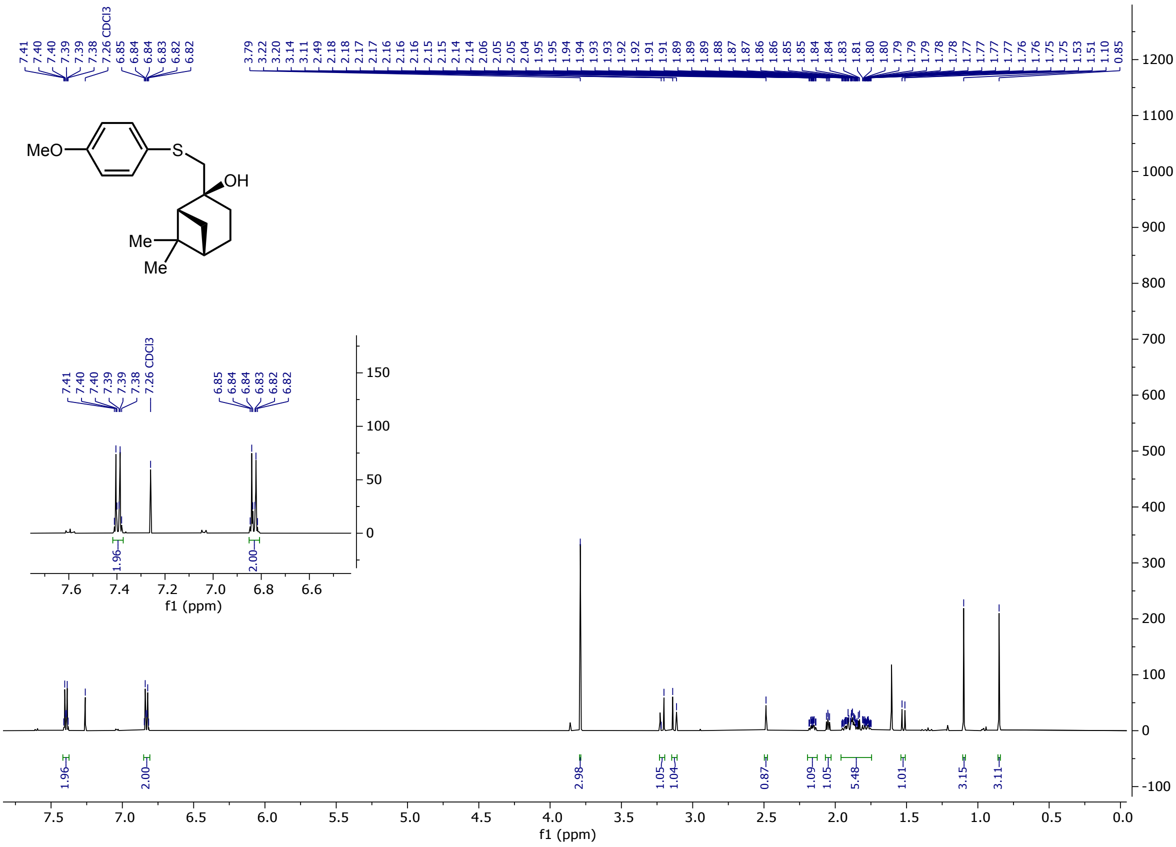

^{13}C NMR (126 MHz, $CDCl_3$) of **3i**

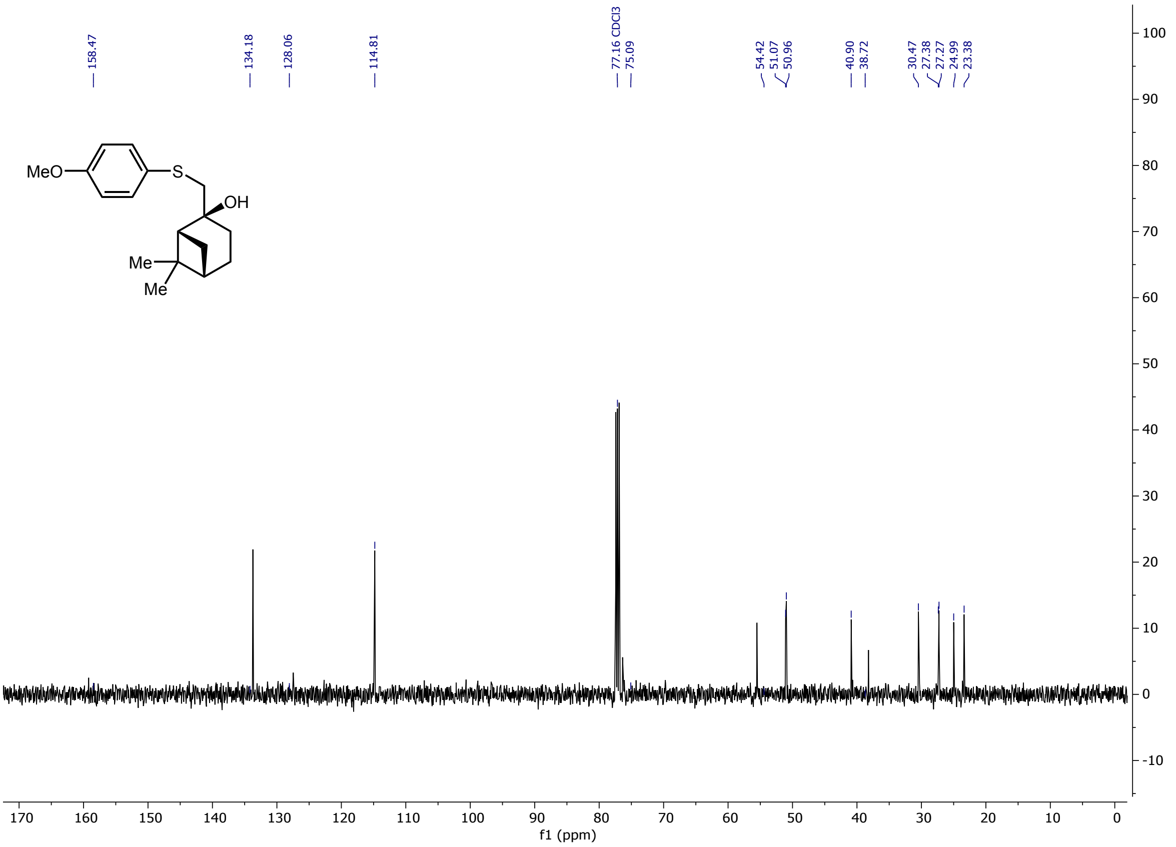

HSQC (400 MHz, $CDCl_3$) of **3i**

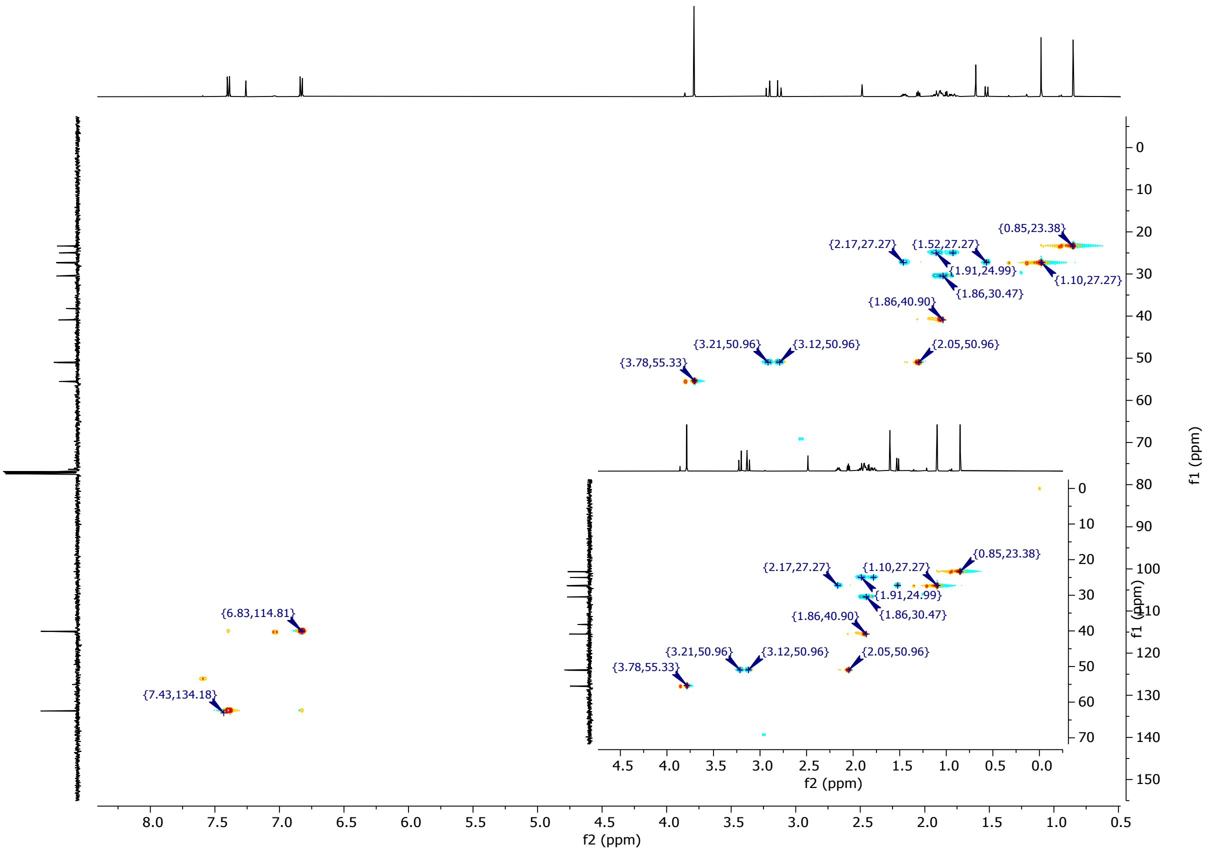

HMBC (400 MHz, $CDCl_3$) of **3i**

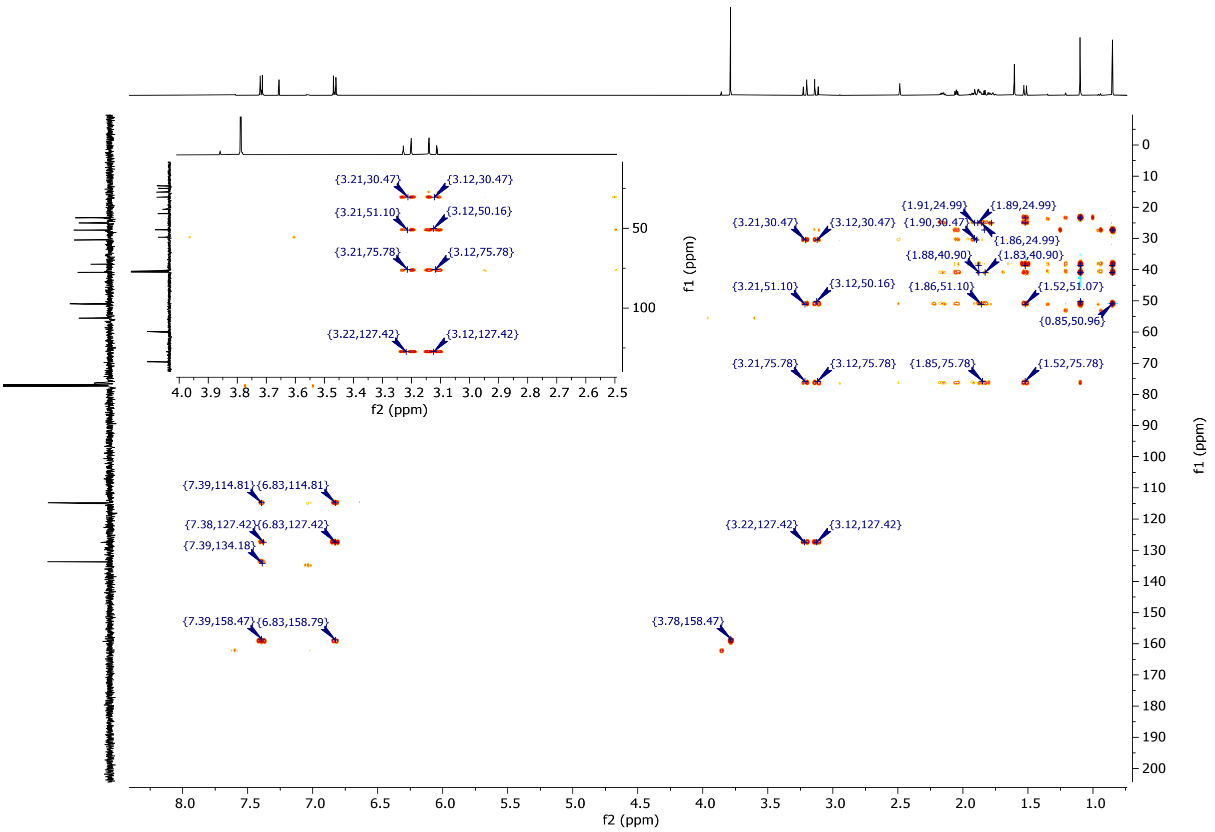

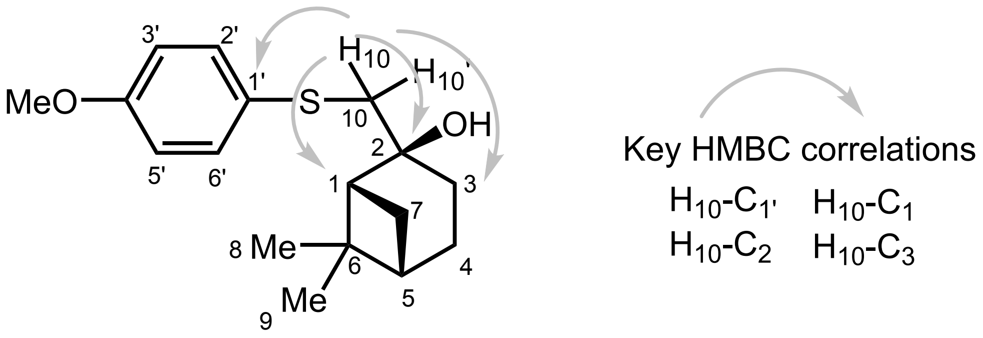

Figure S4. Key HMBC correlations for product **3i**elucidation.

2D NOESY (400 MHz, $CDCl_3$) of **3i**

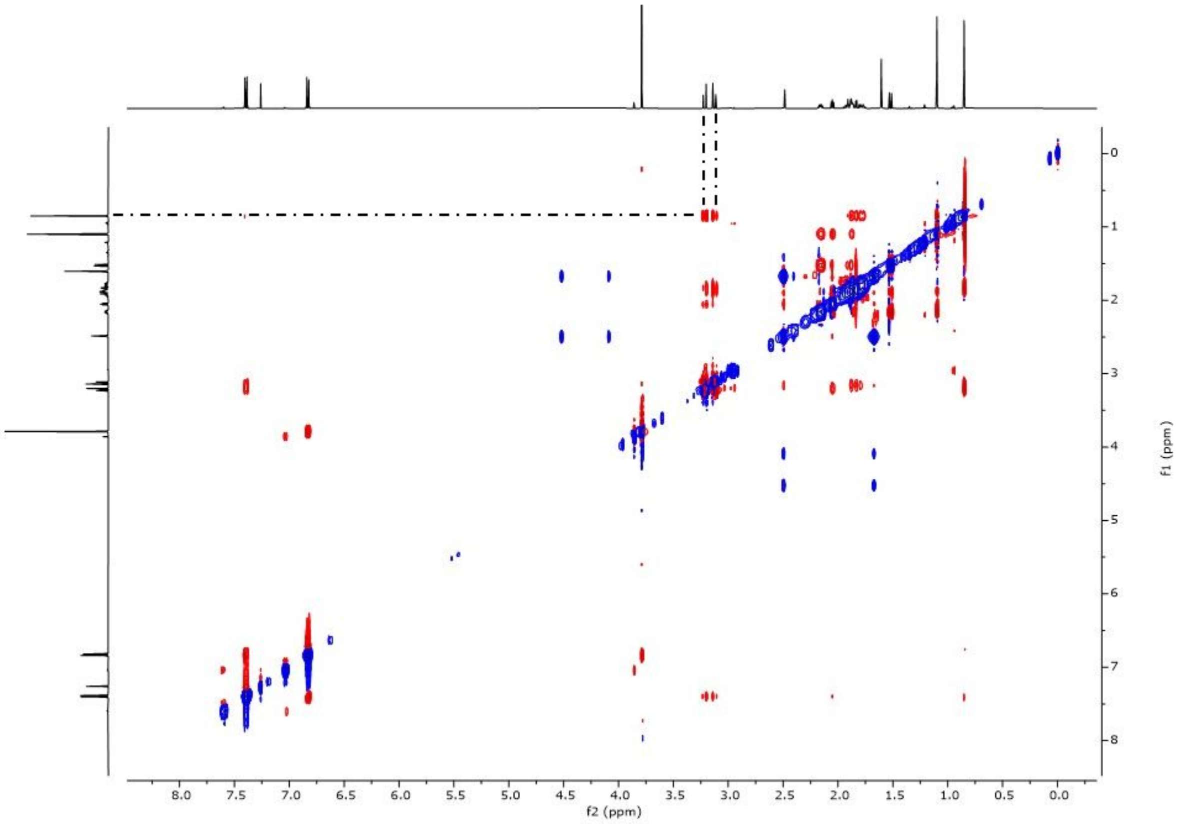

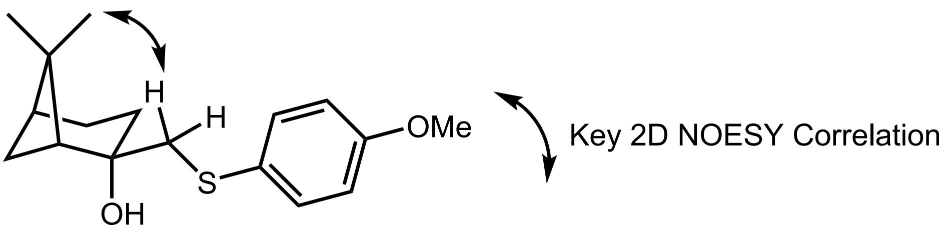

Figure S5. Key NOESY correlation for **3i** elucidation.

^{1}H NMR (500 MHz, CD_3OD) of **3j**

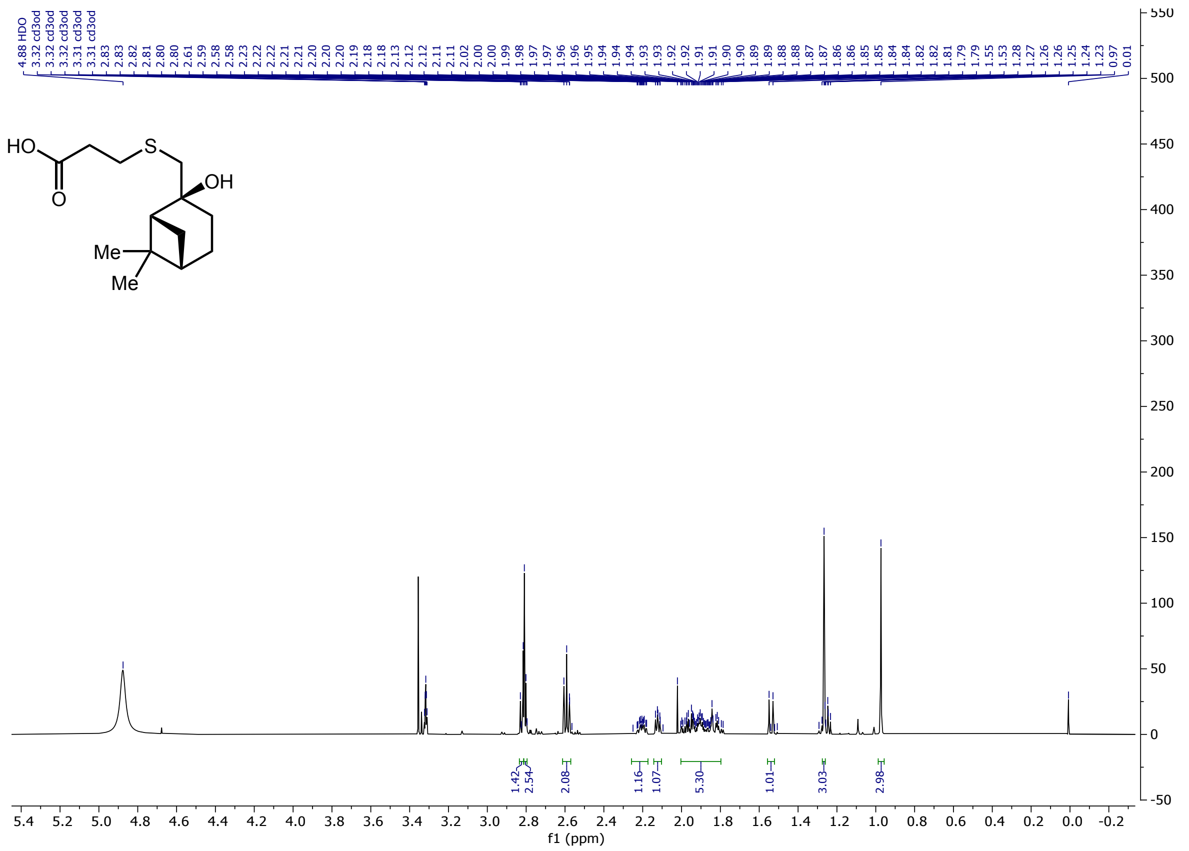

^{13}C NMR (126 MHz, CD_3OD) of **3j**

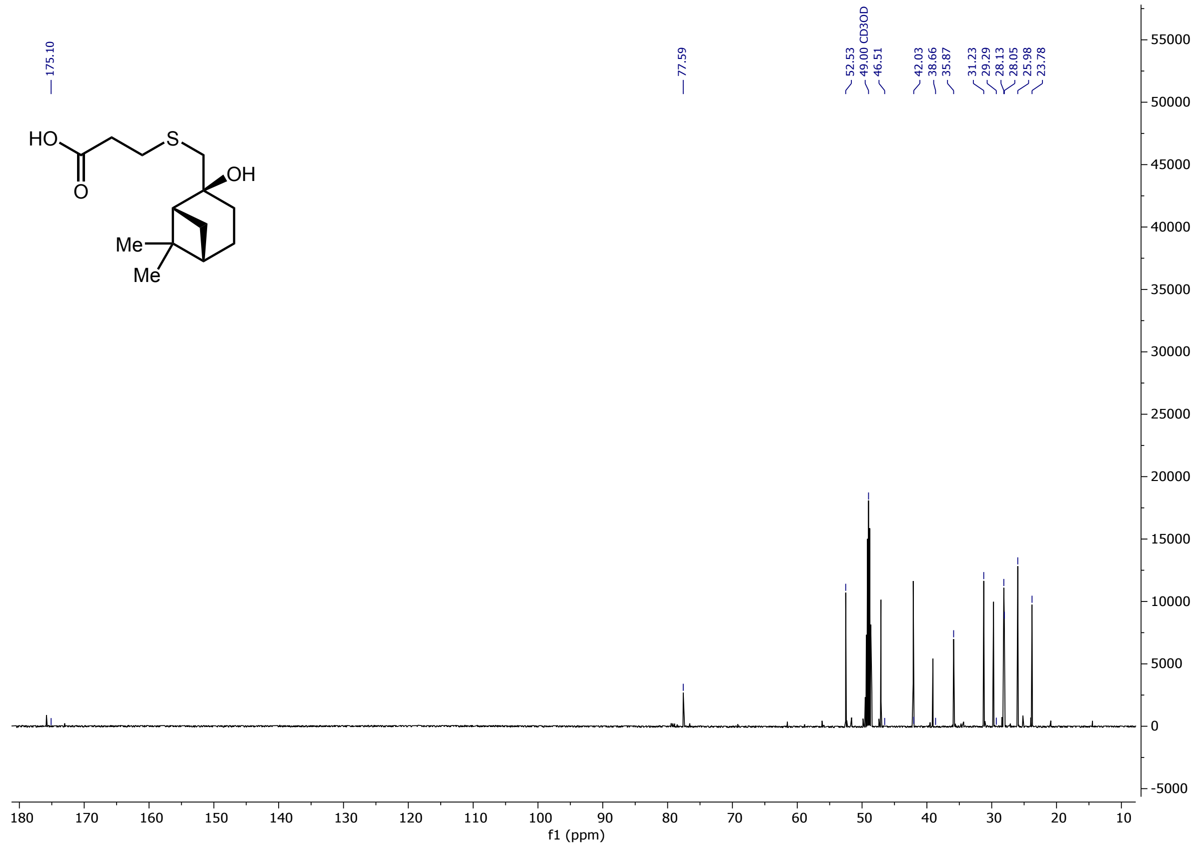

^{1}H NMR (500 MHz, $CDCl_3$) of **3k**

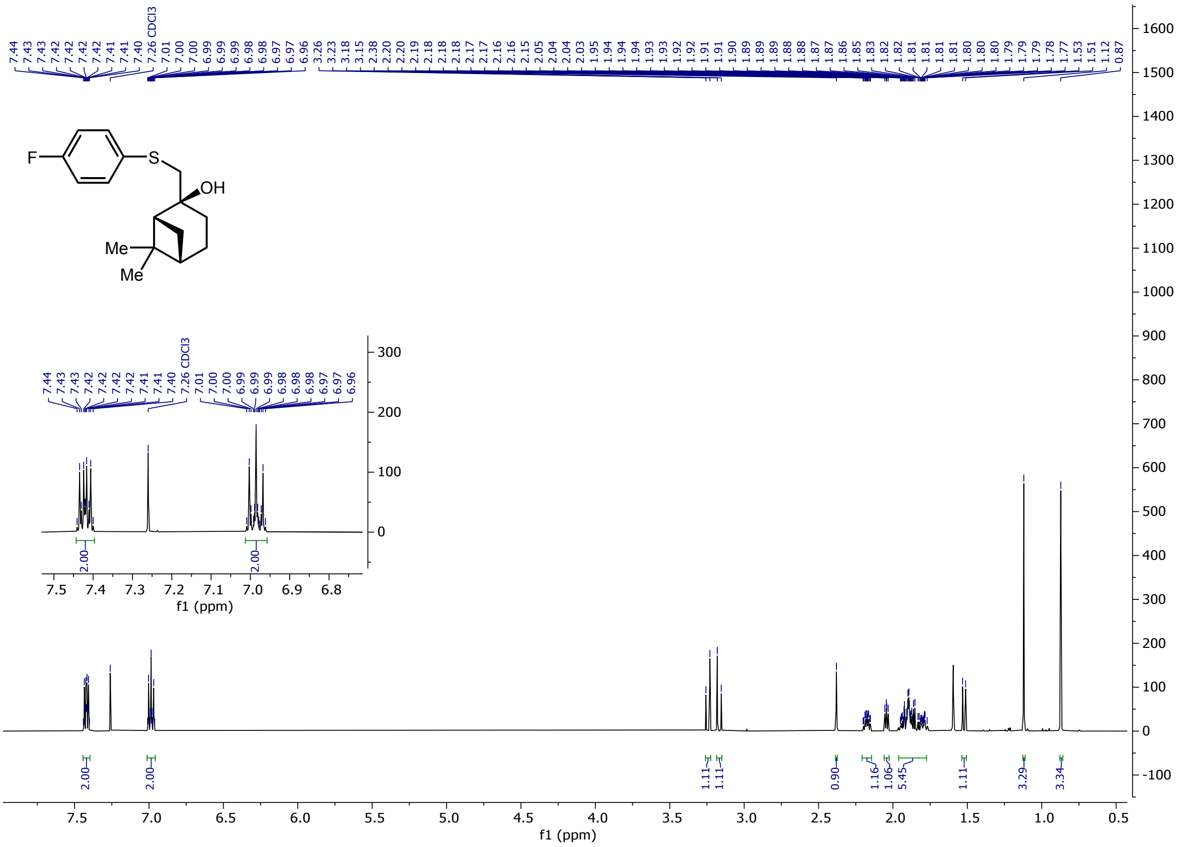

^{13}C NMR (126 MHz, $CDCl_3$) of **3k**

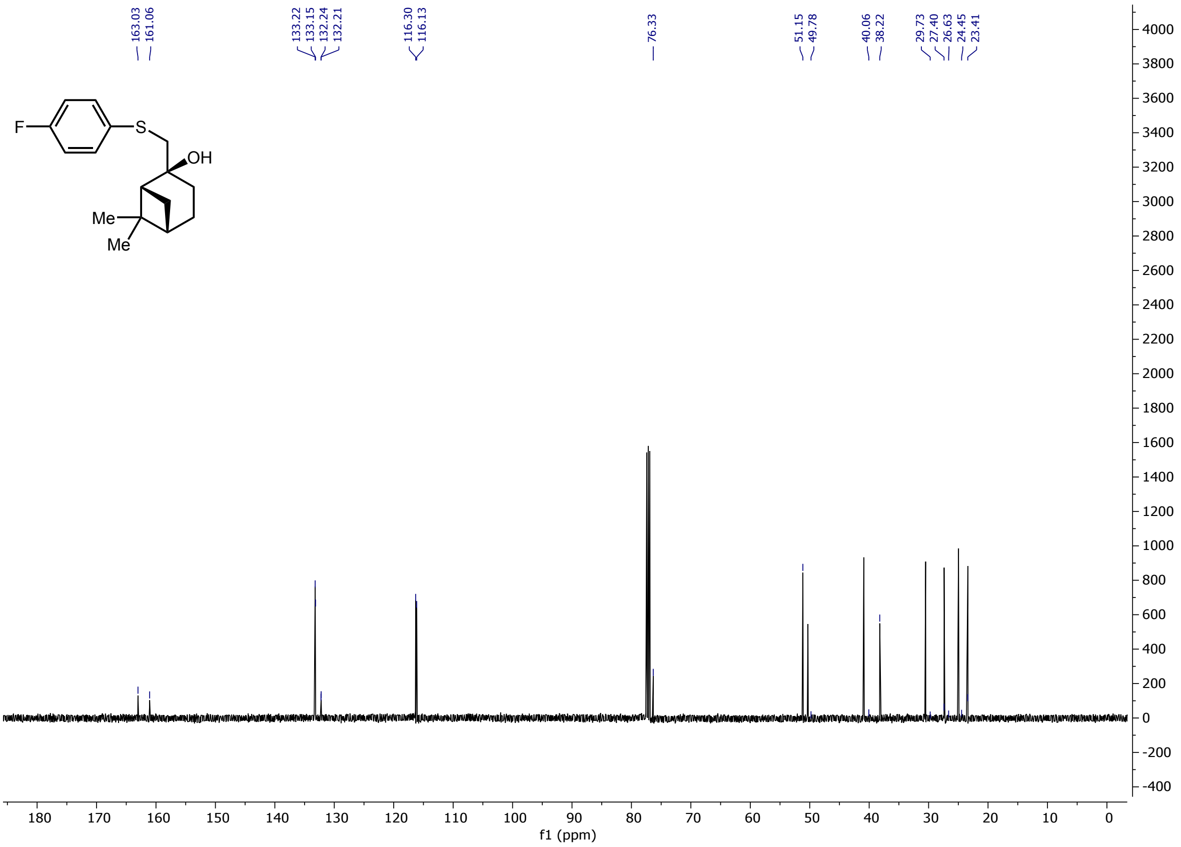

HSQC (500 MHz, $CDCl_3$)of **3k**

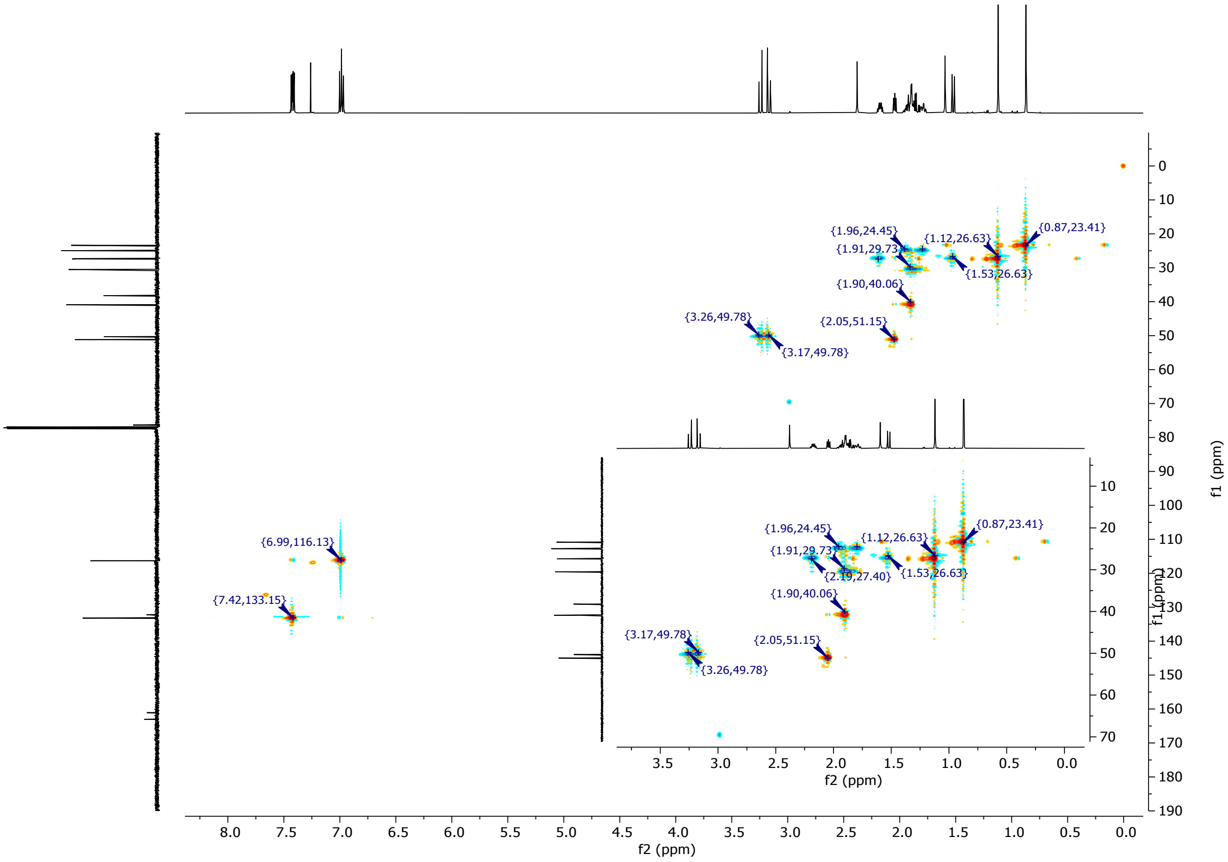

HMBC(500 MHz, $CDCl_3$) of **3k**

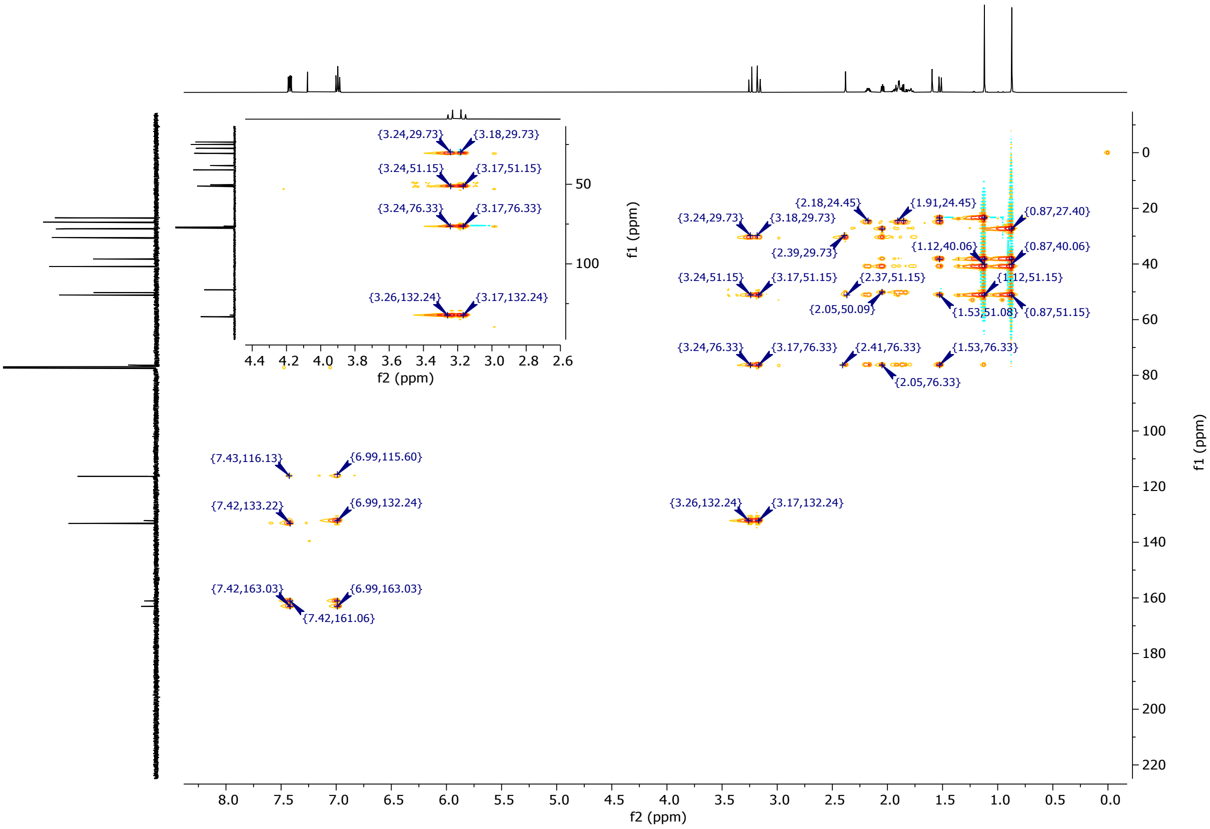

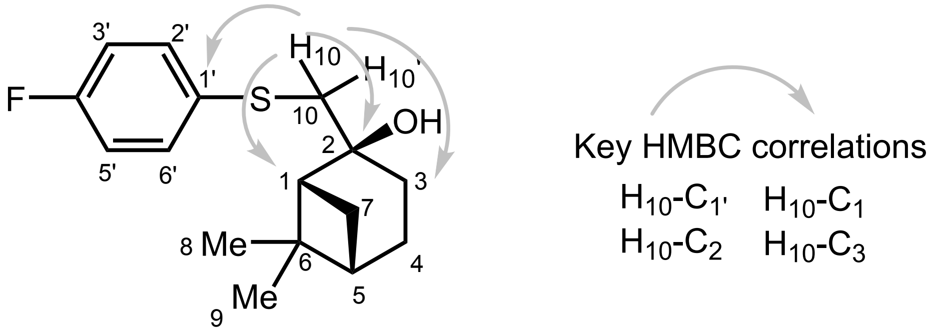

Figure S6. Key HMBC correlations for product **3k** elucidation.

2D NOESY (500 MHz, $CDCl_3$) of **3k**

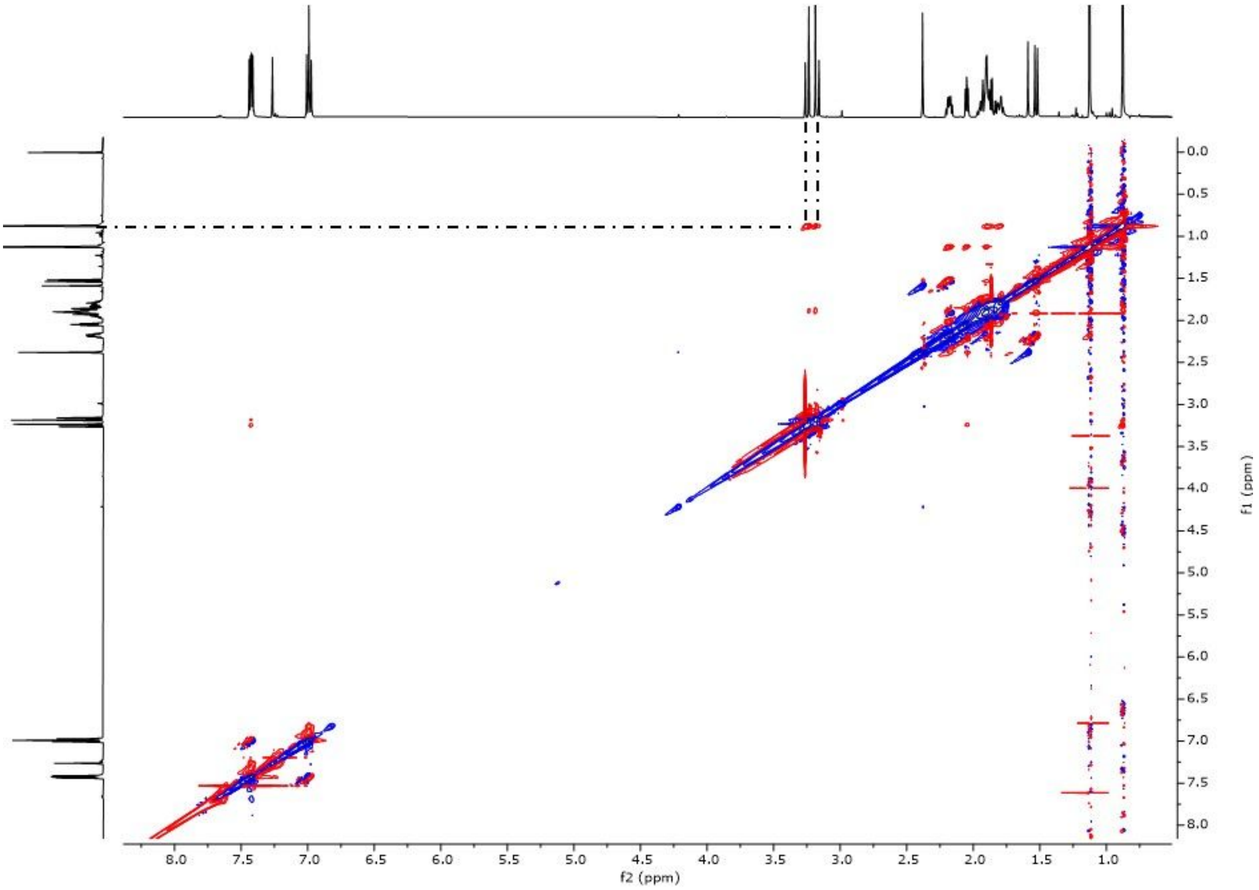

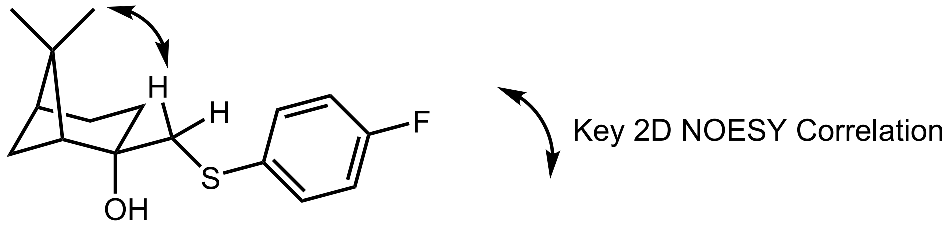

Figure S7. Key NOESY correlations for **3k** elucidation.

^{1}H NMR (500 MHz, CDCl$_3$) of **3l**

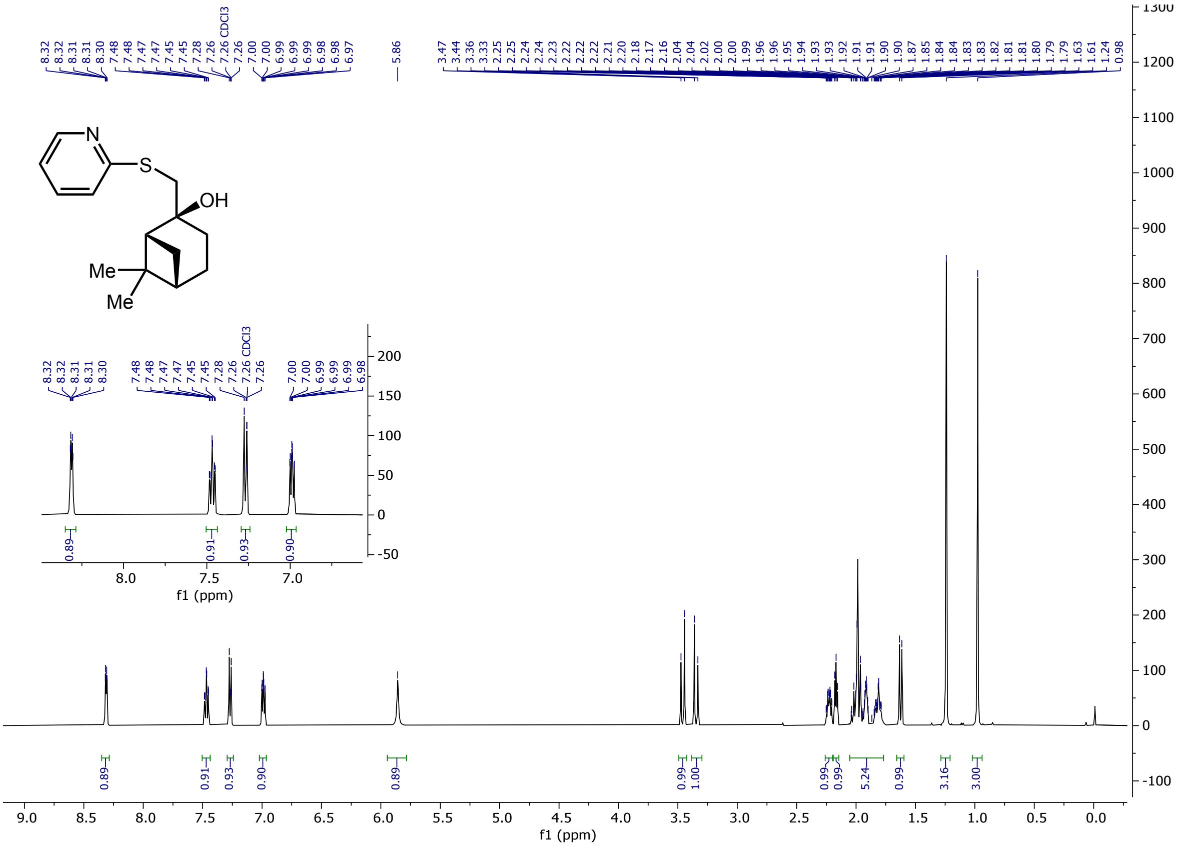

^{13}C NMR (126 MHz, CDCl$_3$) of **3l**

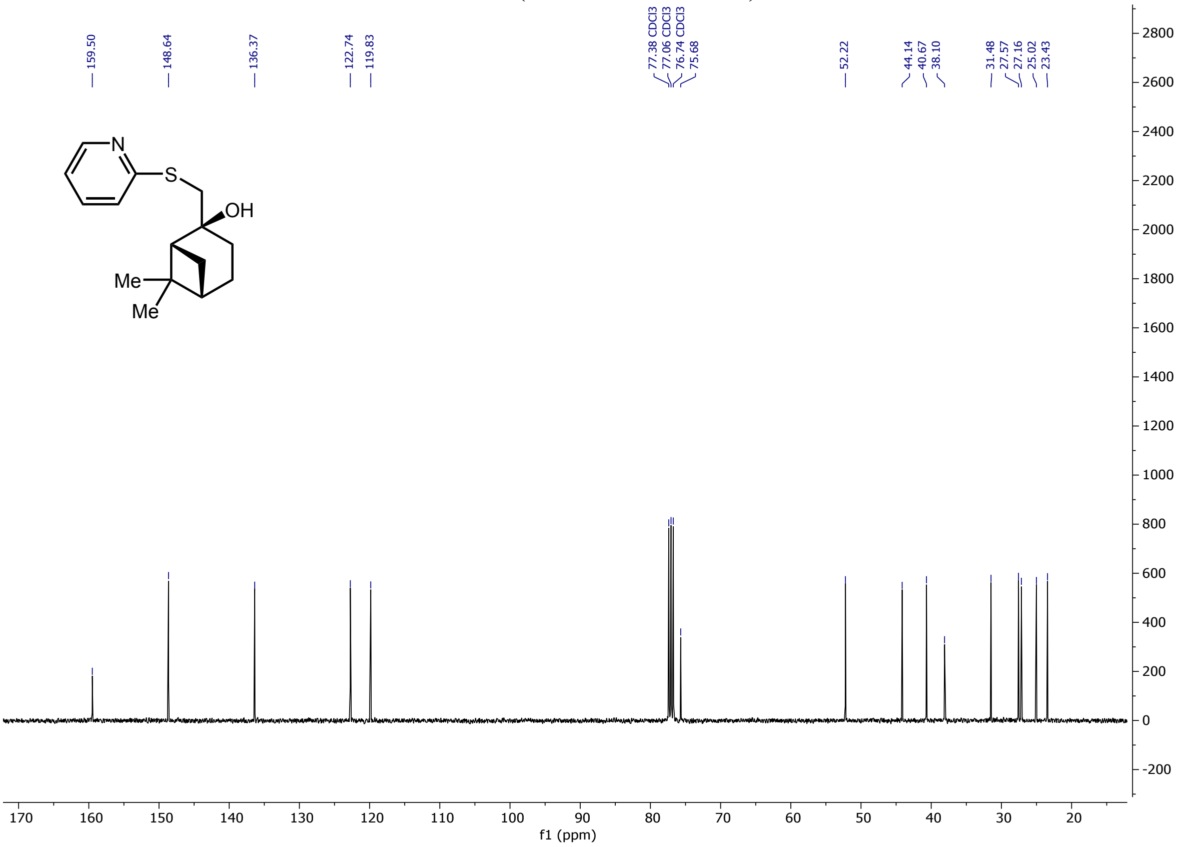

HSQC (500 MHz, $CDCl_3$) of **3l**

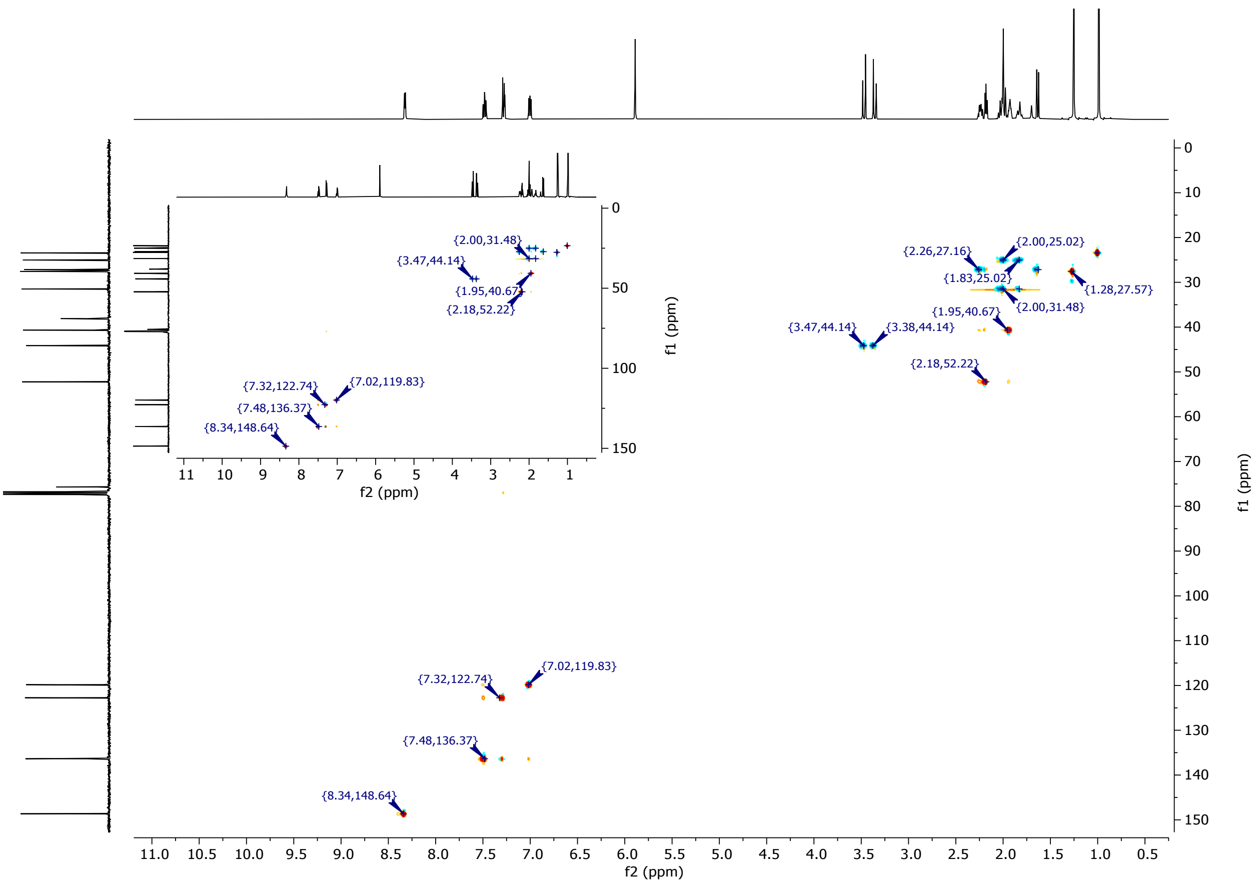

HMBC (500 MHz, $CDCl_3$) of **3l**

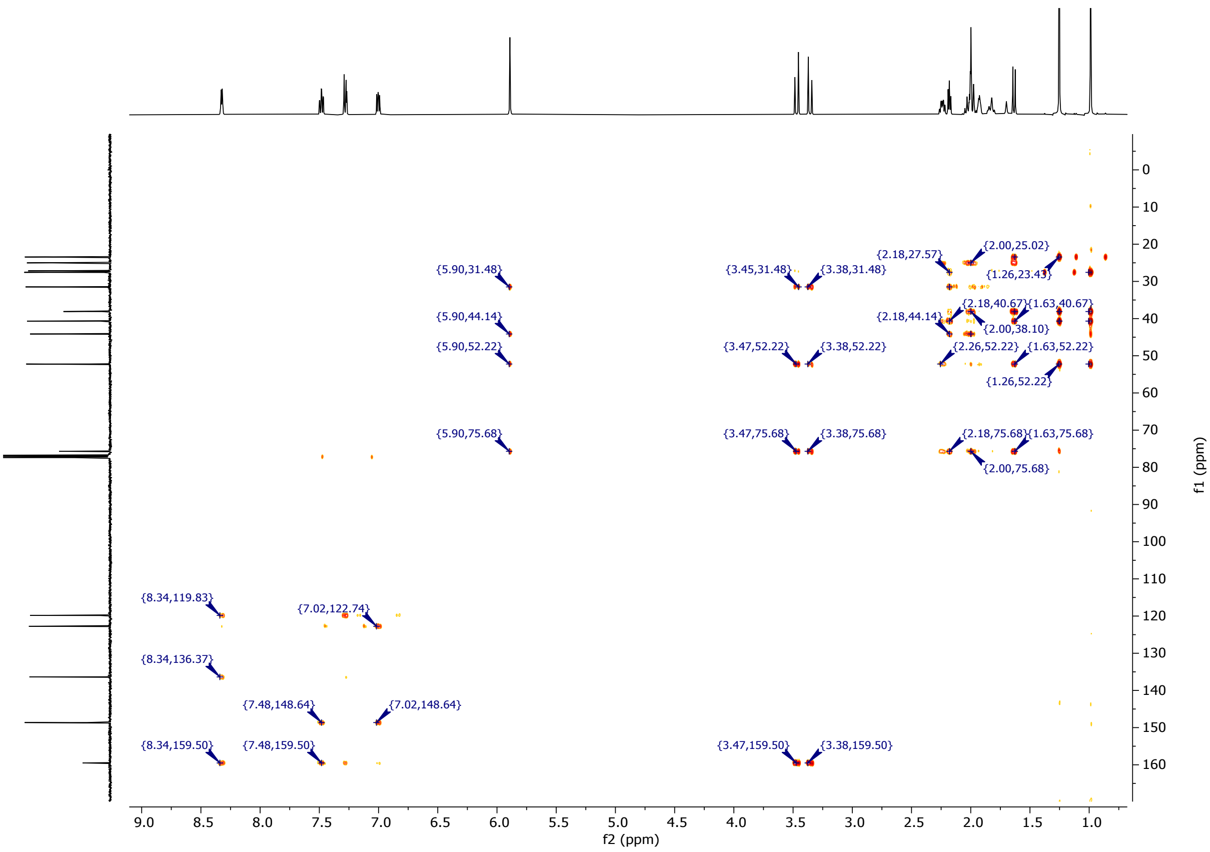

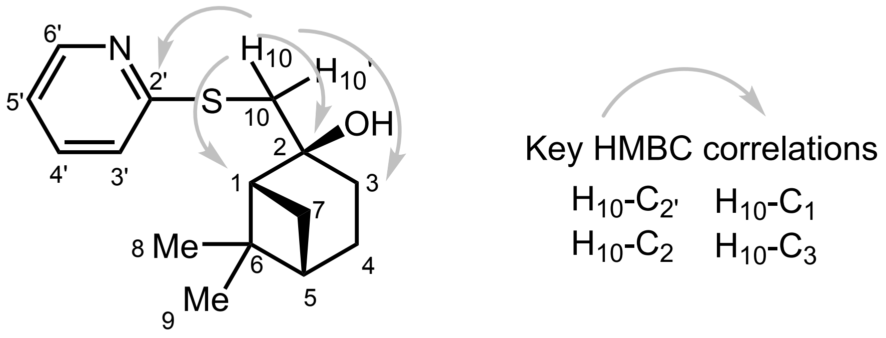

Figure S8. Key HMBC correlations for product **3l** elucidation.

2D NOESY (500 MHz, $CDCl_3$) of **3l**

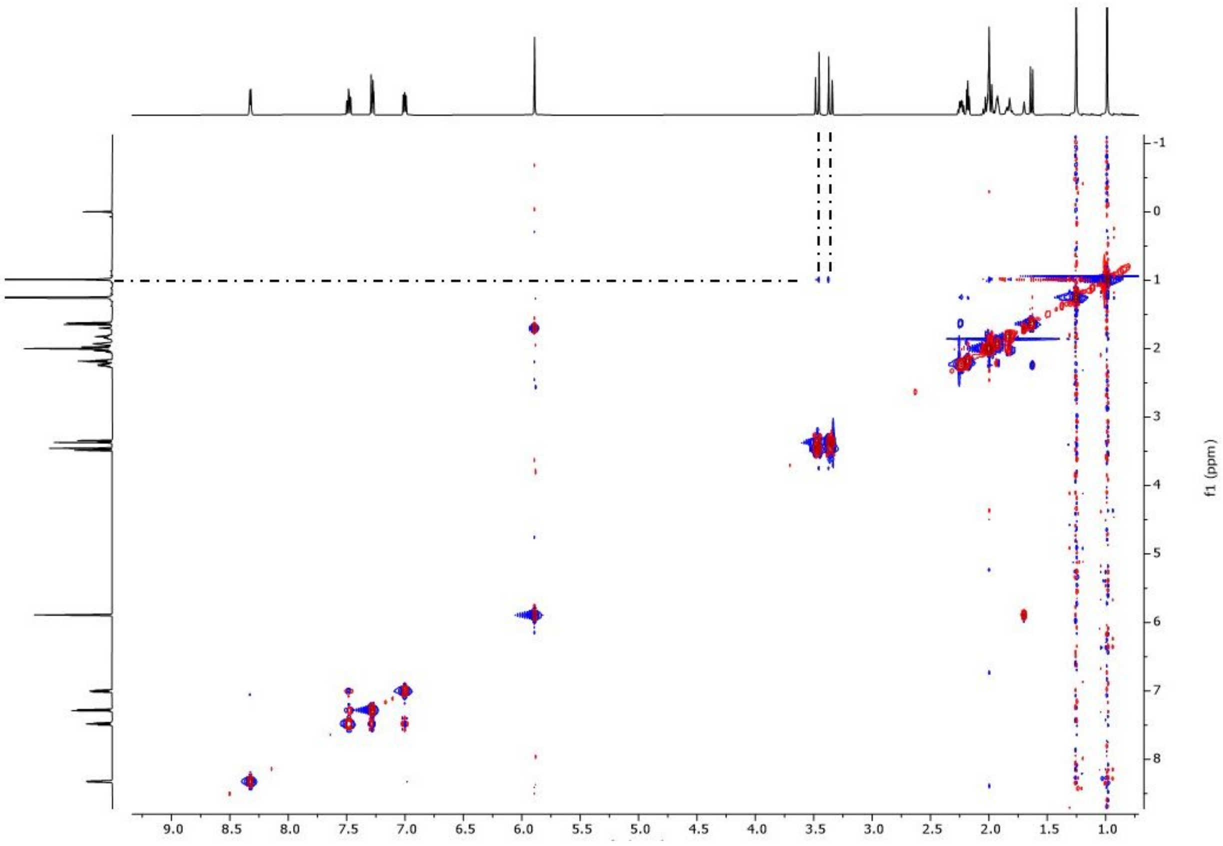

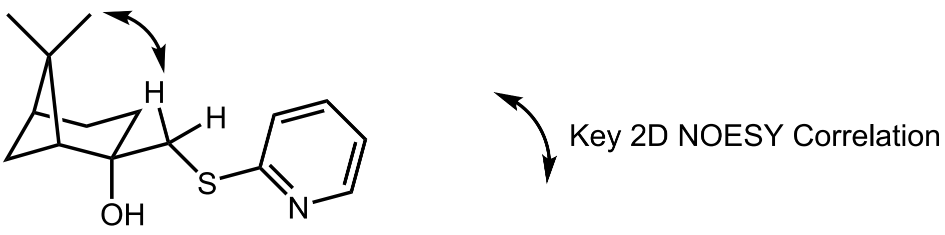

Figure S9. Key NOESY correlations for **3l** elucidation.

^{1}H NMR (500 MHz, $CDCl_3$) of **3m**

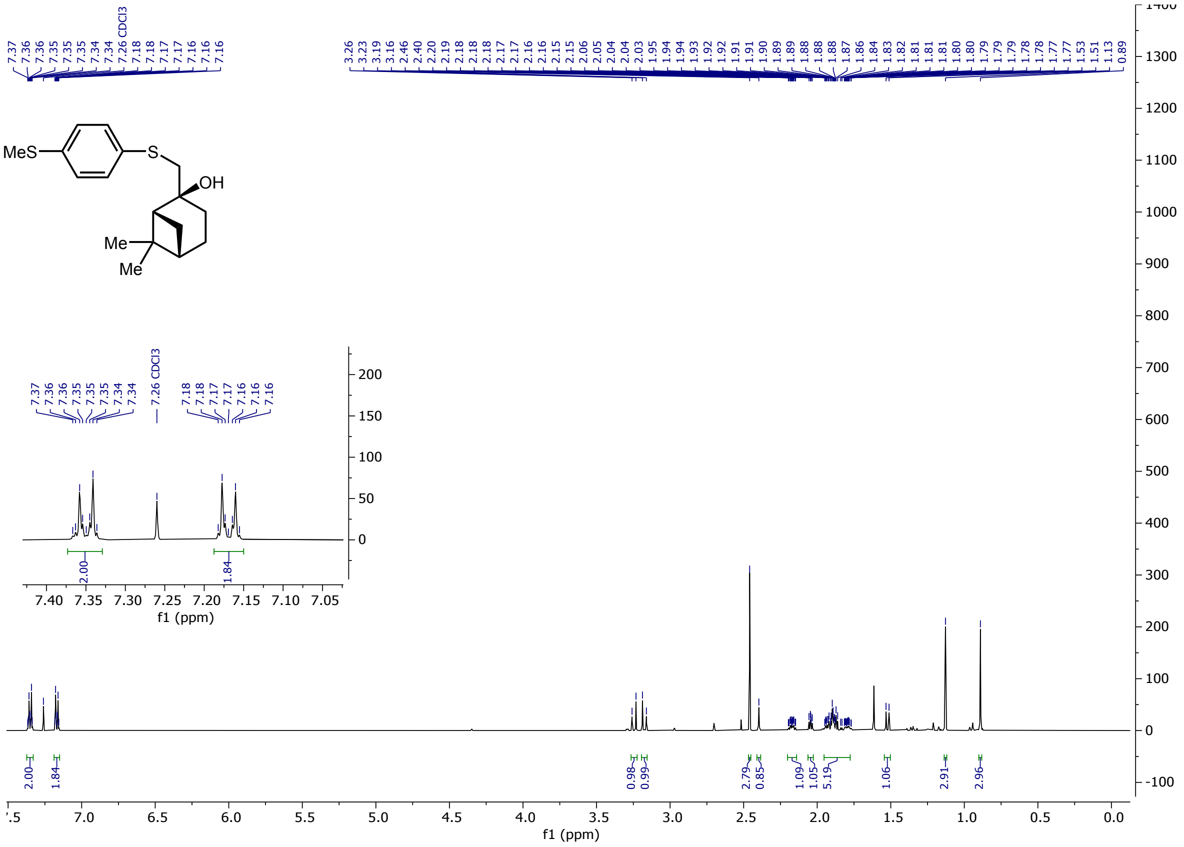

^{13}C NMR (126 MHz, $CDCl_3$) of **3m**

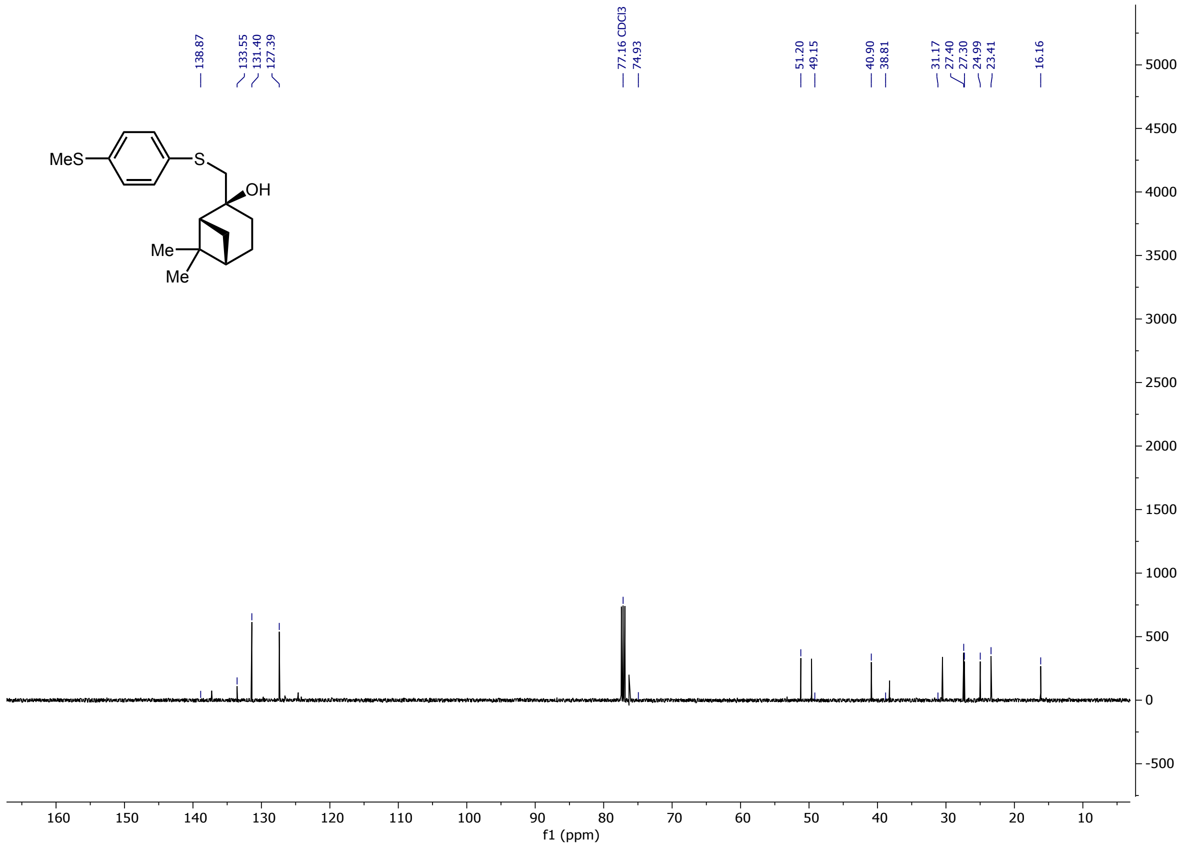

^{1}H NMR (500 MHz, $CDCl_3$) of **3n**

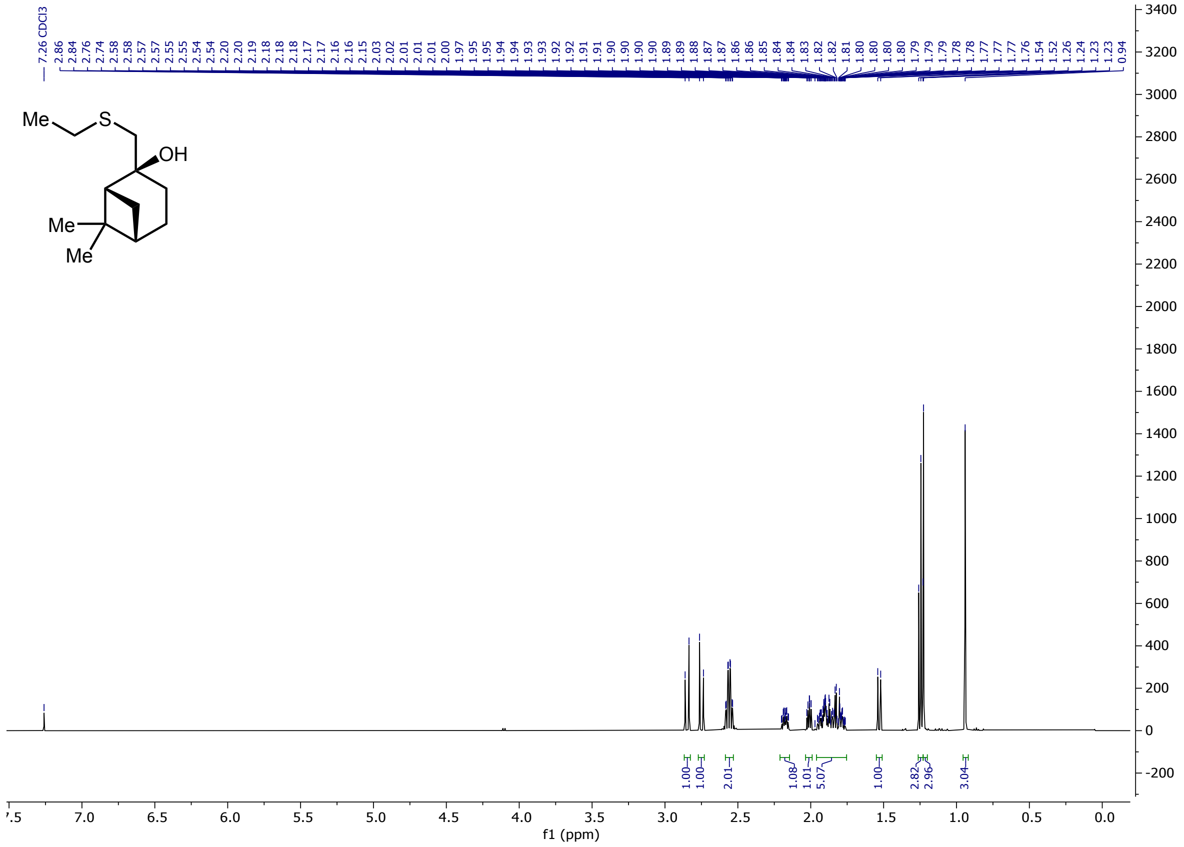

^{13}C NMR (126 MHz, $CDCl_3$) of **3n**

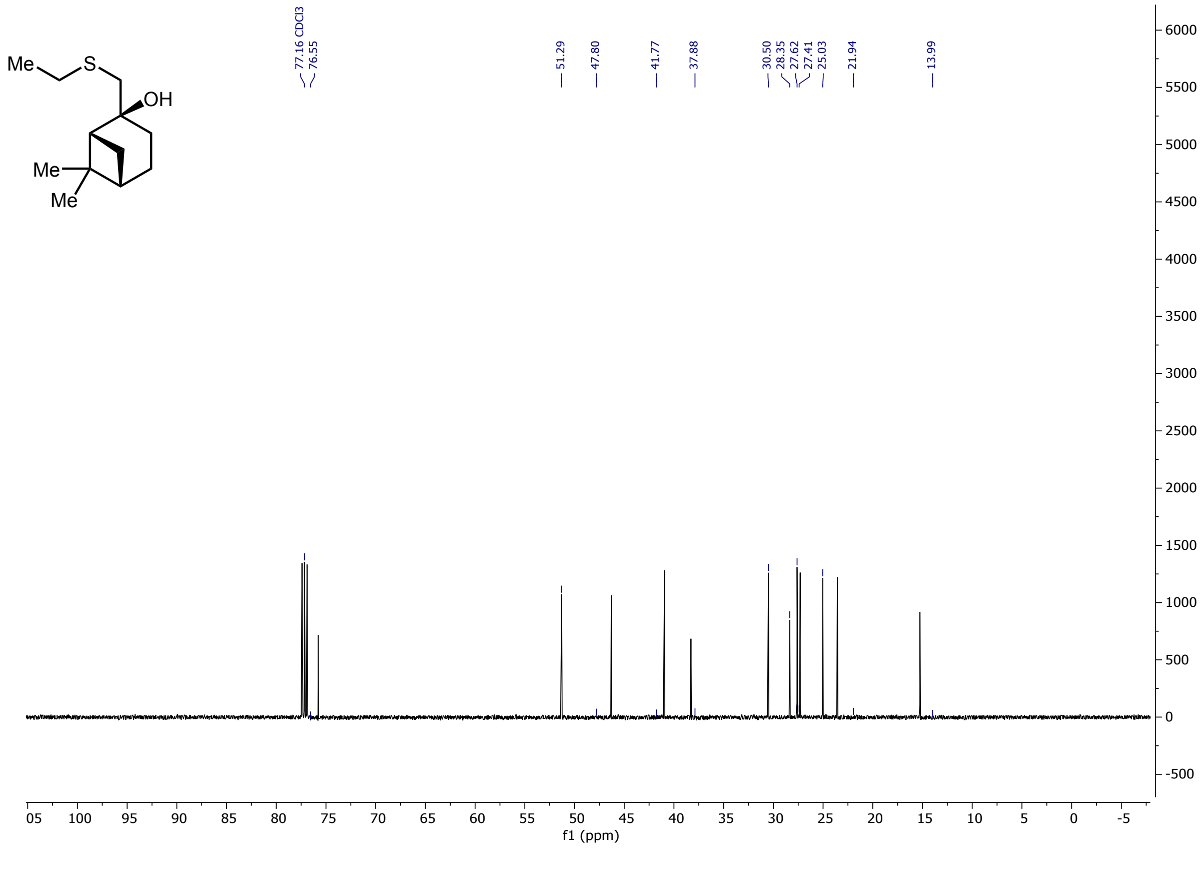

^{1}H NMR (500 MHz, CD_3OD) of **3o**

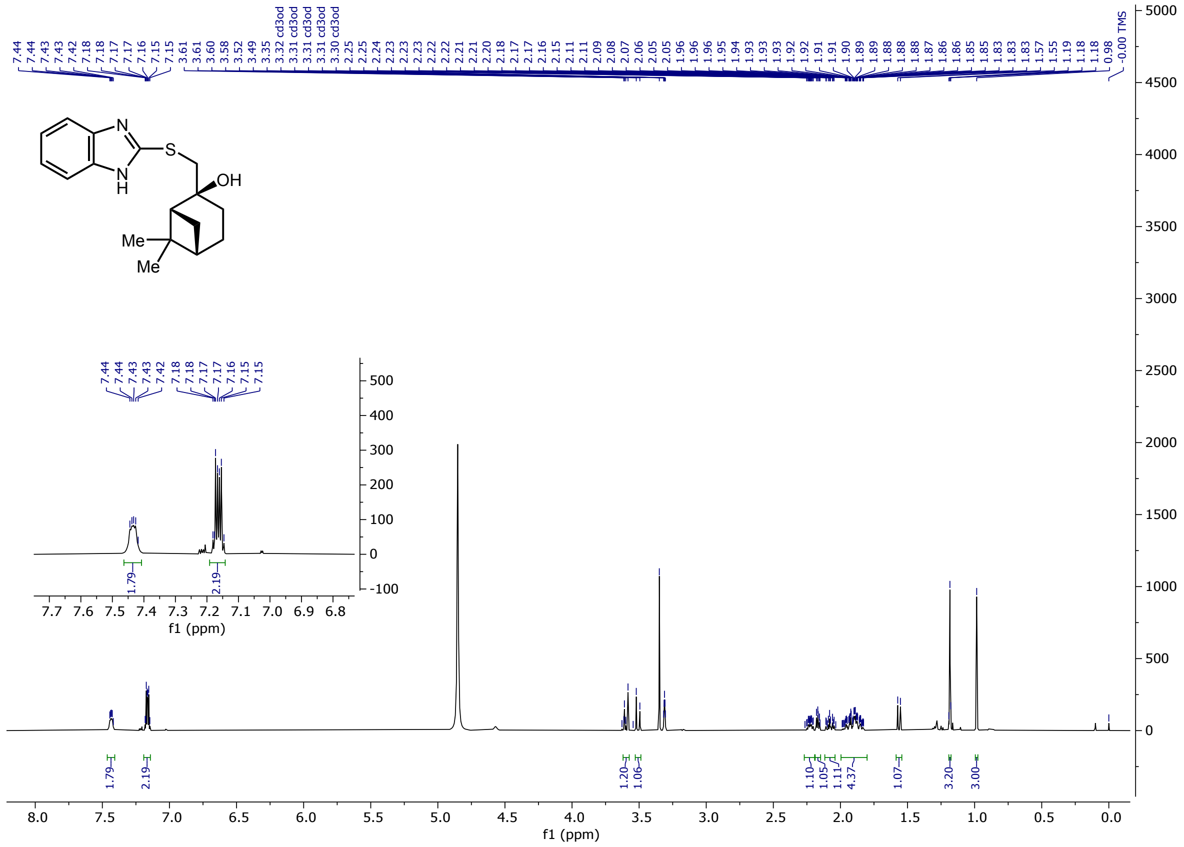

^{13}C NMR (126 MHz, CD_3OD) of **3o**

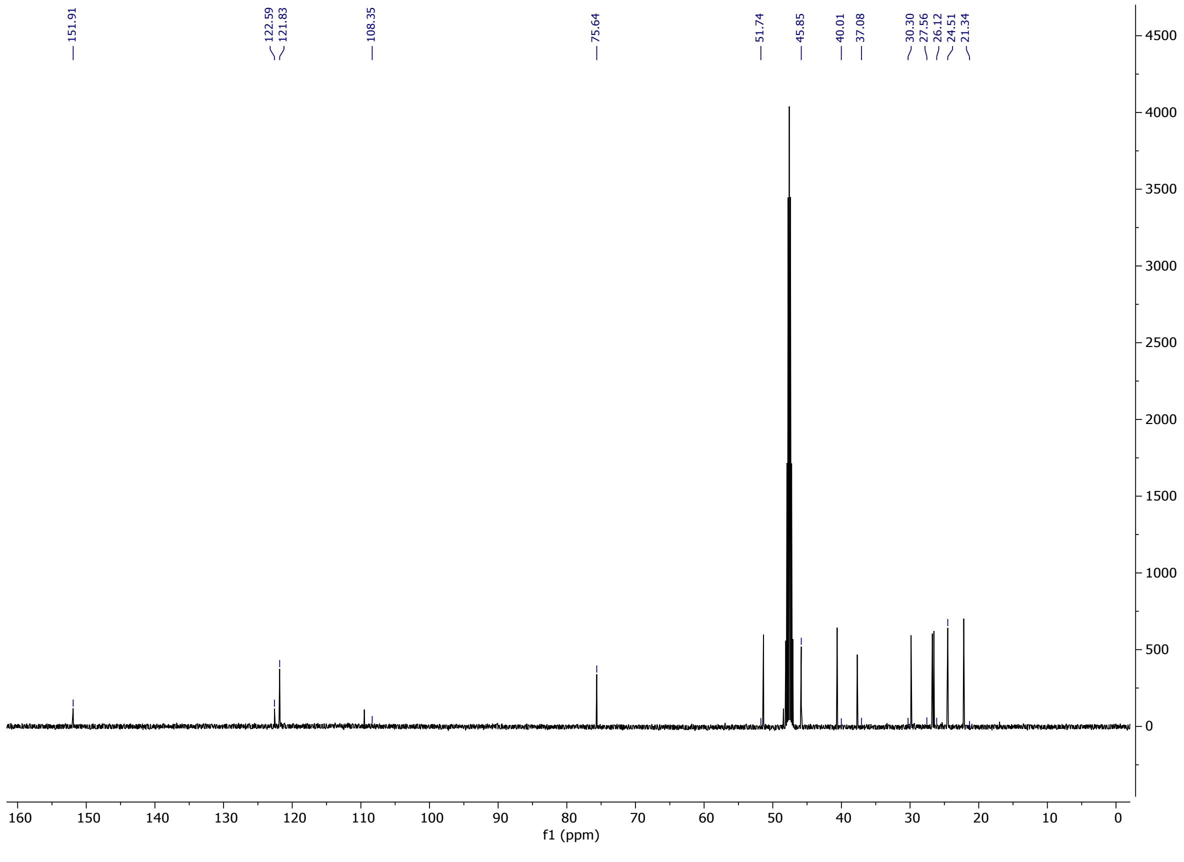

^{1}H NMR (500 MHz, $CDCl_3$) of **3p**

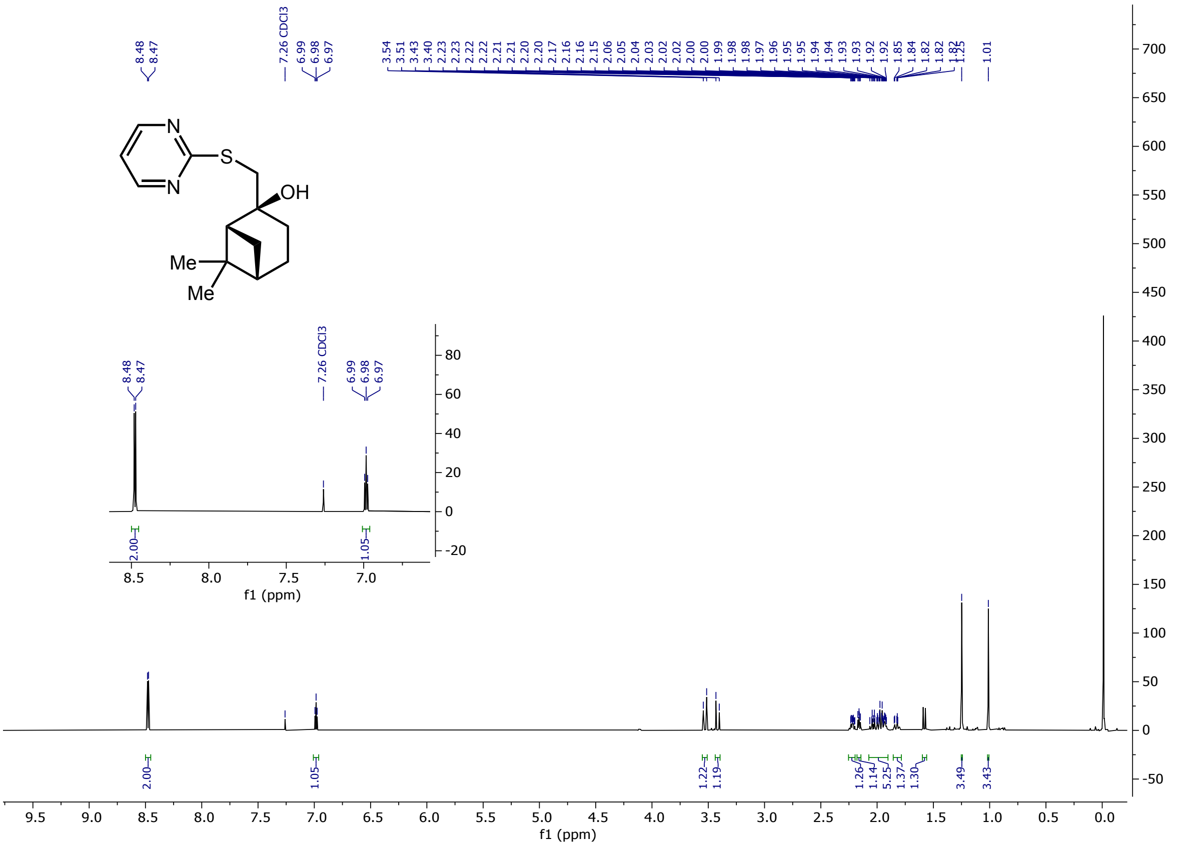

^{13}C NMR (126 MHz, CD_3OD) of **3p**

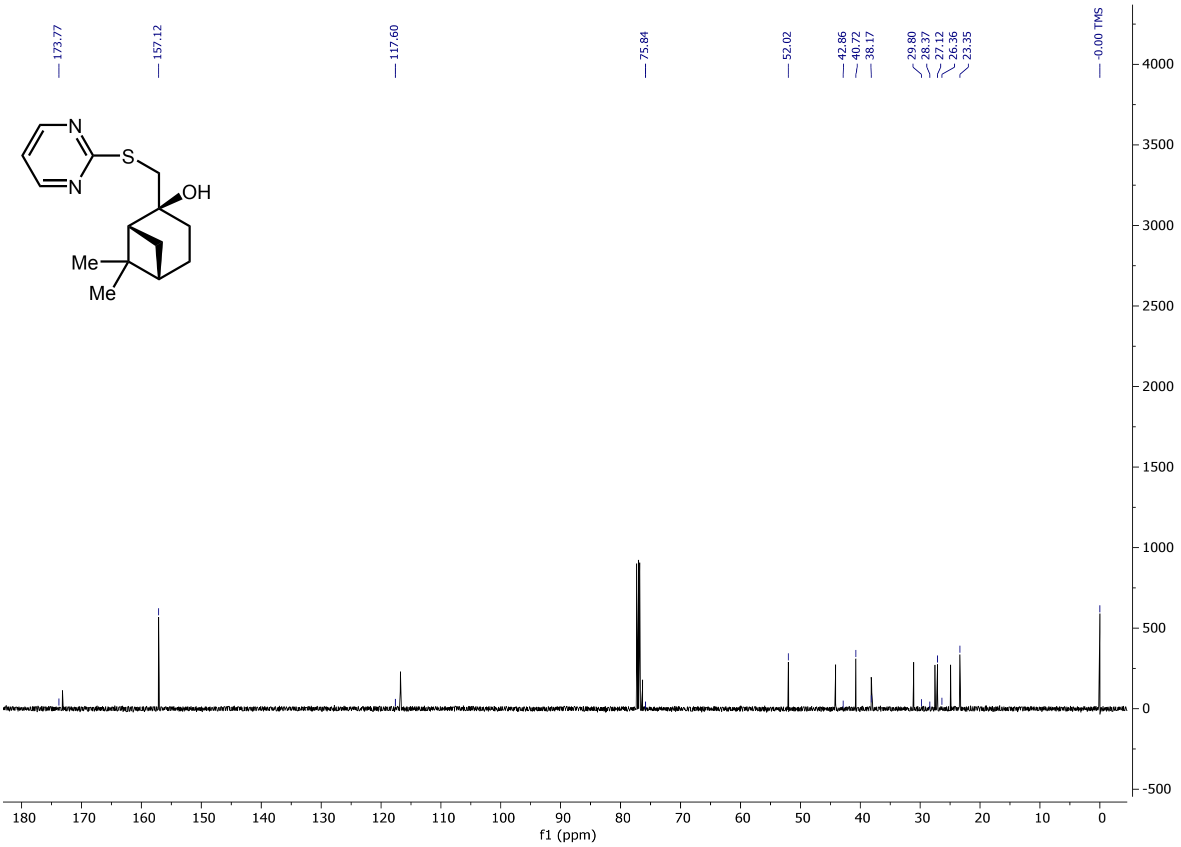

6. Biological evaluation: determination of IC_{50} against promastigote and intracellular amastigote of *Leishmania amazonensis*

6.1. Antipromastigote activity of **3a-p** at fixed concentration of 100 μM

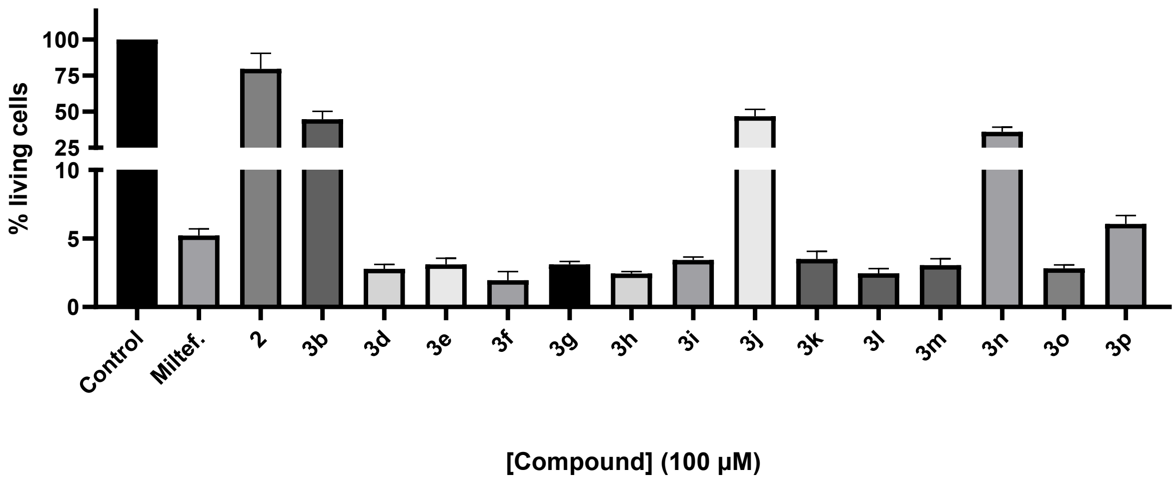

Figure S10. Cell viability of *L. amazonensis* promastigotes after 72 h of incubation with derivatives **3a–3p** at 100 μM. Assays were performed in triplicate.

6.2. Determination of IC_{50} against intracellular amastigote of *L. amazonensis*

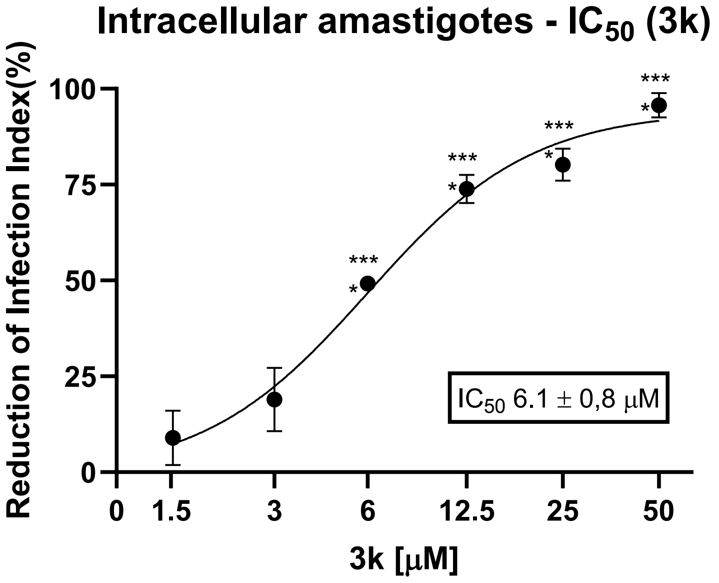

Figure S11. Determination of IC_{50} of **3k** against intracellular amastigote of *L. amazonensis*. The values represent the mean ± standard error of three independent experiments (n=3). The IC_{50} was calculated by nonlinear regression using GraphPad Prism version 10.0. [b]A statistically significant difference compared to the **3k** with the control was observed (**** $p < 0.0001$).

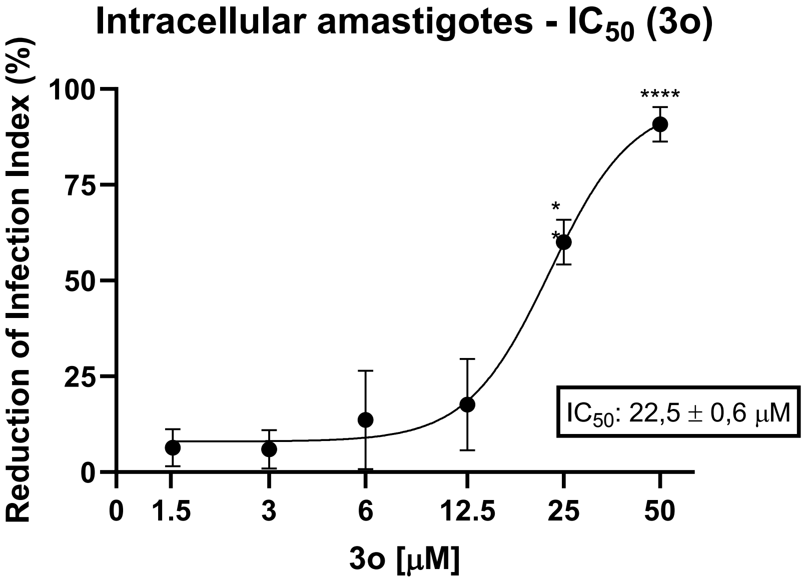

Figure S12. Determination of IC_{50} of **3o** against intracellular amastigote of *L. amazonensis.*The values represent the mean ± standard error of three independent experiments (n=3). The IC_{50} was calculated by nonlinear regression using GraphPad Prism version 10.0.

References

(1) Ali Mcknight, E.; P. Kretekos, N.; Owusu, D.; Lyn Lalonde, R. Technical Note: Preparation and Purification of Atmospherically Relevant Hydroxynitrate Esters of Monoterpenes. *Atmos. Chem. Phys.***2020**, *20* (7), 4241–4254.
